# Mapping the Proteomic Landscape of Pancreatic Cancer: Prognostic Insights and Subtype Stratification

**DOI:** 10.1158/2767-9764.CRC-25-0229

**Published:** 2025-10-23

**Authors:** Adel T. Aref, Jason Grealey, Mohashin Pathan, Zainab Noor, Asim Anees, A.K.M. Azad, Daniela Lee Smith, Erin M. Humphries, Daniel Bucio-Noble, Jennifer M.S. Koh, Erin K. Sykes, Steven G. Williams, Ruth J. Lyons, Natasha Lucas, Dylan Xavier, Sumit Sahni, Anubhav Mittal, Jaswinder S. Samra, John V. Pearson, Nicola Waddell, Olga Kondrashova, Angela Chou, Loretta Sioson, Amy Sheen, Australian Pancreatic Cancer Genome Initiative (APGI), Venkateswar Addala, Lesley Andrews, Jennifer Arena, Ray Asghari, Mo Ballal, Andrew P. Barbour, Claudio Bassi, Maria Beilin, Andrew V. Biankin, Nicola Blackburn, Mark E. Brooke-Smith, Diego Chacon Fajardo, Cecilia R. Chambers, David K. Chang, Lorraine A. Chantrill, John Chen, Angela Chou, Andrew D. Clouston, Vincenzo Corbo, Peter H. Cosman, Thomas R. Cox, Amitabha Das, Stephan B. Dreyer, Tanya Dwarte, Krishna Epari, James R. Eshleman, Jonathan W. Fawcett, Kynan Feeney, David Fletcher, Cindy Forrest, Anthony J. Gill, Annabel Goodwin, Peter Grimison, Sean M. Grimmond, Michael Hatzifotis, David Herrmann, Hilda A. High, Peter Hodgkinson, Oliver Hofmann, Oliver Holmes, Ralph H. Hruban, Kasim Ismail, Nigel B. Jamieson, Gloria Jeong, Amber L. Johns, James G. Kench, Judy Kirk, Rita T. Lawlor, Conrad Leonard, Ruth J. Lyons, Duncan McLeod, R. Scott Mead, Neil D. Merrett, Anubhav Mittal, Sanjay Mukhedkar, Adnan Nagrial, Felicity Newell, Nan Q. Nguyen, Mehrdad Nikfarjam, Max Nobis, Katia Nones, Thomas J. O’Rourke, Marina Pajic, Virginia Papangelis, Nick Pavlakis, John V. Pearson, Brooke Pereira, Sean Porazinski, Daniel A. Reed, Shona Ritchie, Alice Russo, Andrew R. Ruszkiewicz, Jaswinder S. Samra, Charbel Sandroussi, Aldo Scarpa, Kellee Slater, Allan Spigellman, Alina Stoita, Michael Texler, Paul Timpson, Katherine Tucker, Claire Vennin, Nicola Waddell, David Williams, Christopher L. Wolfgang, Scott Wood, Chris Worthley, Nikolajs Zeps, Peter G. Hains, Phillip J. Robinson, Qing Zhong, Roger R. Reddel, Anthony J. Gill

**Affiliations:** 1 ProCan, Children’s Medical Research Institute, Faculty of Medicine and Health, The University of Sydney, Westmead, Australia.; 2 Cancer Division, The Kinghorn Cancer Centre, Garvan Institute of Medical Research, University of New South Wales, Darlinghurst, Australia.; 3 University of Sydney, Sydney, Australia.; 4 The University of Notre Dame Australia, Sydney, Australia.; 5 QIMR Berghofer Medical Research Institute, Brisbane, Australia.; 6 NSW Health Pathology, Department of Anatomical Pathology, Royal North Shore Hospital, St Leonards, Australia.; 7 Cancer Diagnosis and Pathology Research Group, Kolling Institute of Medical Research, St Leonards, Australia.

## Abstract

Pancreatic ductal adenocarcinoma (PDA) is an aggressive malignancy that lacks reliable biomarkers to guide treatment decisions. Effective prognostic tools are needed to improve its clinical management. We conducted a comprehensive proteomic analysis on 115 PDA patient samples with matched adjacent normal tissue. A 20-protein diagnostic panel was identified (LGALS1, ANXA2, LGALS3BP, CTSD, S100P, COL12A1, SFN, THBS2, CTHRC1, THBS1, SERPINB5, LAMC2, POSTN, CEACAM6, CTSE, PLEC, PKM, S100A11, TAGLN2, ALDOA). Consensus clustering analysis identified four prognostic proteomic subtypes. Subtypes with poorer prognoses exhibited upregulation of neutrophil degranulation, extracellular matrix remodeling, focal adhesion, Mesenchymal Epithelial Transition, collagen formation, and PI3K-Akt-mTOR-related pathways, indicating a predominance of basal-like and activated stromal features. In tumors with homologous recombination deficiency or Catalogue of Somatic Mutations in Cancer Signature-3, several immune-related proteins were enriched. An 18-protein (PURB, SDCBP2, CD2BP2, GALM, SERPINA3, OAS3, FAN1, ZPR1, KRT2, NUDT2, SMNDC1, SERPINA4, CUTA, WDR36, POSTN, CLEC11A, PEX14, and PI4KA) risk score was developed and validated using multicox regression analyses with LASSO regularization. The risk score demonstrated independent prognostic significance for overall survival and recurrence, and was validated in an independent proteomic dataset generated using a different proteomic technology. This study thus introduces four novel prognostic PDA subtypes, and an 18-protein risk score validated in an independent dataset, which shows promise for improving survival prediction and could serve as a valuable tool for personalized treatment guidance.

**Significance:**

The findings from this study have significant implications for the future of pancreatic cancer management. By identifying a 20-protein panel with diagnostic and screening potential, this research provides a foundation for developing early detection tools for PDA, an aggressive cancer with limited treatment options. The classification of PDA into four proteomic subtypes with distinct prognostic outcomes paves the way for subtype-specific therapeutic approaches, allowing clinicians to better stratify patients based on their risk profiles. Additionally, the validated 18-protein risk score, which enhances survival prediction and operates independently of existing clinical variables, represents a promising tool for personalized prognostic assessments. Incorporating these proteomic-based biomarkers into clinical practice could improve diagnostic accuracy, guide individualized treatment decisions, and ultimately enhance patient outcomes in PDA. This study underscores the potential of proteomic profiling to improve cancer treatment by providing targeted, actionable insights into tumor biology.

## Introduction

Pancreatic ductal adenocarcinoma (PDA) is a highly aggressive type of cancer (1), with more than 50% of patients with PDA being diagnosed at a metastatic stage (with 5-year survival of 3%; ref. 2). Even for patients diagnosed with early-stage PDA, the recurrence rate is around 75% after surgical resection, with a 5-year survival less than 20% (3). Currently, the first line of treatment for PDA is chemotherapy combinations, including 5-fluorouracil, oxaliplatin, and irinotecan or a combination of gemcitabine and nab-paclitaxel (3). However, these regimens cause considerable toxicity and lack relevant biomarkers to guide treatment decisions (4–6). To date, the use of targeted therapy in PDA has been rather limited. Despite the high prevalence of *KRAS* mutations (more than 90% of all PDA cases), attempts to target KRAS or its downstream pathways (RAS–RAF–MEK and PI3K–AKT) have thus far yielded disappointing results overall (6, 7). Although targeting less prevalent alterations, such as *KRAS*^G12C^, *BRCA1/2*, *NTRK*, and *BRAF *mutations, and mismatch repair deficiency, has shown some promising results, their incidence is low, with less than 7% of patients with PDA being potentially eligible for these treatments (6, 8).

Another major challenge that contributes to PDA’s poor prognosis is the tumor microenvironment that promotes tumor progression and resistance to chemotherapy. PDA is characterized by dense stroma, which is abundant in cancer-associated fibroblasts and extracellular matrix (ECM)–related proteins (including TGFβ, laminins, fibronectin, S100, annexins, and collagen types I, III, IV, V, VI, and XV) and has low microvascular density and minimal immune cell infiltration (9–11). This complex and challenging microenvironment has contributed to the limited efficacy of immunotherapy in PDA (12).

Analyzing the molecular profile of pancreatic cancer remains a priority to increase the understanding of this disease and identify more robust markers that can guide treatment. PDA is characterized by four highly prevalent mutations (in *KRAS*, *SMAD4*, *TP53*, and *CDKN2A*), yet none of them were found to be associated with long-term survival (13). Additionally, gene-based classifications have been previously explored; however, the results were not consistent and seemed to be confounded by the stromal component of PDA (14–17).

In this study, we performed a proteomic analysis of PDA tumors and their matched normal samples from 115 patients in the Australian Pancreatic Cancer Genome Initiative (APGI; ref. 18). This cohort had histopathologic, clinical, follow-up, and molecular data, including *KRAS* status, homologous recombination deficiency (HRD) status, and Catalogue of Somatic Mutations in Cancer (COSMIC) signatures. Through these proteomic analyses, we were able to identify a diagnostic panel, identify a novel prognostic classification, and define several potential therapeutic targets within the different subgroups. We also developed a proteomic-based prognostic risk score that was validated in an independent Clinical Proteomic Tumor Analysis Consortium (CPTAC) dataset.

## Materials and Methods

### Sample collection and data generation

Fresh-frozen samples were obtained from 125 patients, and a 30-μm section was taken from each sample for proteomic analyses, plus a directly adjacent 4-μm section for hematoxylin and eosin staining. The APGI study and the related analyses, including the proteomic analyses performed at Children’s Medical Research Institute, were conducted in accordance with the Declaration of Helsinki and approved by the relevant ethics committees, 2019/ETH08915, 2023/ETH02130, and 2019/ETH02039 (HREC/17/WMEAD/63) and X16-0293 (HREC/11/RPAH/329). Sample sectioning and preparation were all done onsite at the ProCan laboratory, Children’s Medical Research Institute, Sydney. Samples were prepared for mass spectrometry (MS) using the Heat ‘n Beat method (19) in batches designed using a Markov chain Monte Carlo algorithm with 3-year survival status as a blocking variable, and the resultant peptide content was quantified via UV spectrophotometry (Implen NP80). The resulting quantities were then used to adjust the peptide mixture to 0.5 μg/μL to load 2 μg total onto the MS instrument.

Data-independent acquisition MS (DIA-MS) data were acquired (19) from a total of 596 DIA-MS runs of biological samples in technical triplicate and 87 HEK293 control samples. The three replicates for each sample were run on different MS instruments. Samples were analyzed by LC/MS, with an Eksigent nanoLC 425 HPLC (SCIEX) operating in microflow mode, coupled online to a TripleTOF 6600 (SCIEX) mass spectrometer. Peptide spectra were acquired using 100 variable windows in DIA mode. The acquisition parameters were a lower m/z limit of 350, upper m/z limit of 1,250, and window overlap (Da) of 1.0; CES was set at 5 for the smaller windows, then 8 for larger windows, and 10 for the largest windows. MS2 spectra were collected in the range of m/z 100 to 2,000 for 30  milliseconds in high-resolution mode, and the resulting total cycle time was 3.2 seconds.

### Quality control and protein matrix generation

DIA data were searched using DIA-NN (20) as described (19). Peptide quantification was performed using DIA-NN with a global filter *Q* value ≤ 0.01, and only proteotypic peptides were retained. The peptide matrix was generated by summing precursor intensities for 47,519 peptides across 596 runs. Proteins were then quantified using MaxLFQ implemented using the DIA-NN R package (https://github.com/vdemichev/diann-rpackage).

The quality control (QC) process resulted in the exclusion of 14 runs in total. Three runs (from one sample) were removed because of low protein detection (<400 proteins for three runs). Three samples (nine runs) were also removed because histopathology review of the adjacent hematoxylin and eosin–stained 4-μm section revealed the absence of cancer tissue. Finally, two runs (from two different samples) were removed based on low Pearson replicate correlation (*R* < 0.70; cohort mean *R* = 0.89). The final protein matrix was therefore composed of 582 runs and 5,611 proteins.

Before performing downstream analyses, the protein matrix was transformed to include, at most, one normal sample (if available) and one tumor sample per patient. This required averaging across replicates, and tumor content was taken into account to increase tumor-relevant biology when consolidating patients that had multiple tumor samples. Formally, the samples were graded on tumor content by a pathology review into the following categories: 0%, <20%, 20% to 50%, 50% to 80%, and >80% tumor content. To consolidate the samples into one tumor sample per patient, the samples were ranked according to their tumor content. If two samples from the same patient differed by two or more cancer categories ordinally (e.g., <20% and >80% cancer content), then only the sample with the larger tumor content was included in the analyses. After this, nonhuman proteins were excluded, which resulted in a final working protein matrix of 176 (61 adjacent normal and 115 tumor) samples with 5,599 proteins.

### Differential abundance and pathway enrichment analyses

Differential abundance analyses (DAA) were performed using Limma (21) and eBayes (v3.56.2). A given protein was determined to be differentially abundant if the nominal *P* value $P_{nominal}<\;0.05$ and the protein had a |log (fold change)| > 1 between the two given comparison groups. The DAA compared tumors (*n* = 115) against normal samples (*n* = 61) across 5,599 proteins. All further downstream analyses were conducted using tumor samples only (*n* = 115). For each set of DAA results, enrichment analyses were performed using ClusterProfiler (v4.8.2; ref. 22) and ReactomePA (v1.44.0; ref. 23). The pathway enrichment analyses (PEA) were executed using enrichPathway, enrichWP, and enrichGO functions (across all three ontologies of molecular function, biological process, and cellular component) all with a *P* value threshold of 0.05 and Benjamini–Hochberg multiple testing correction.

### Imputation

Missing values in the proteomic data were handled through imputation using the random forest–based method missForest (v1.5) using default settings (maximum of 10 iterations, 100 trees; ref. 24). Protein intensities were centered and scaled after imputation for consensus clustering and survival analyses.

### Consensus clustering analyses

Using the set of imputed and scaled proteins only from tumor samples (*n* = 115), consensus clustering was performed using the top 25% (*n* = 1,399) of proteins with the highest median absolute deviation (MAD) within this cohort. The software package ConsensusClusterPlus (v1.64.0; ref. 25) was used to perform the consensus clustering using Spearman correlation and partitioning around medoids as a clustering algorithm with 5,000 repetitions, 80% item resampling, and 100% protein resampling. The final number of clusters of four was chosen based on the silhouette width, cluster consensus, and cumulative distribution function curves.

### Survival analyses and building the risk score

Cox regression modeling was used for survival analyses. Time to event (overall survival, 3-year survival, and recurrence) in months was calculated as the time from diagnosis to the time of death, death at or before 3 years (3-year survival), or recurrence, respectively. To build the proteomic signature and the risk score, survival analysis was performed on only a set of scaled and imputed proteins with top 25% MAD. Initially, a univariate Cox regression analysis was performed for each of the MAD-filtered proteins to reduce the number of proteins for multivariate Cox regression. From this list, 235 proteins were statistically significant with *P* < 0.05. These 235 proteins were then used as input for 200 runs of multivariate LASSO-regularized Cox regression with 20-fold cross-validation using *glmnet* (v4.1.8). Each run returned a list of proteins with nonzero coefficients, and proteins were sorted in descending order according to the ratio of runs (relative frequency) in which they have nonzero coefficients. The top 30 proteins were selected to be included in a multivariate Cox model with recursive feature selection (stepwise Akaike information criterion in both directions), which resulted in a panel of 18 proteins. These proteins were then used to calculate the proteomic risk score ($S_{j}$) for the *j*th patient (26) using the formula

$$\begin{array}{c} S_{j}Risk\;Score\;=\;\overset{n}{\underset{i\;=\;1}{\sum }}\beta_{i}X_{ji} \end{array}$$ A

in which n is the number of proteins, ${IX}_{ji}$ is the log-transformed, imputed, and scaled intensity of *i*th protein from the *j*th patient, and $\beta_{i}$ is the natural logged HR for the *i*th protein from the Cox proportional hazards model. This score was then dichotomized using the midpoint of the range of risk score values into high- and low-risk values, which were utilized in Kaplan–Meier analyses in which statistical significance of survival was tested using the log-rank test, and *P* values were reported.

### Validation of the risk score using CPTAC data

Proteomic data from the Commons study PDC000341 were downloaded, including isobaric tandem mass tag labeling–based proteomics of treatment-naïve pancreatic tumors. The proteomics data contained median-normalized intensity proteomes at the gene level. Only tumor samples were selected for this analysis, which, after merging with the required survival information, included 92 samples. A total of 11,631 proteins were present in this CPTAC dataset. The risk score was recalculated within the CPTAC cohort utilizing (Eq. A), and the coefficients were estimated from our cohort. This risk score was used to dichotomize the patients into high (*n* = 23) and low (*n* = 69) risk based on the 75th percentile of the proteomic signature within the CPTAC data, and then a Kaplan–Meier survival curve was plotted for the two groups, and statistical significance of survival was estimated using the log-rank test.

### Sanger cell line drug response data

Proteomic and drug response data from the SANGER cell lines are publicly available (27). The proteomic data were generated on the same platform at ProCan. This database includes target proteins for each drug and their related target pathways. Filters were applied to include only targets with skew < −1 and *r*^2^ > 0.3 to ensure robustness. The resulting database was used to identify drugs and their targets by mapping it with the corresponding differentially abundant proteins (DAP) within each group of interest in our differentially abundant analyses.

## Results

### Cohort description

This cohort included 128 resected fresh-frozen PDA samples and 76 matched adjacent normal samples from 125 patients who underwent surgical resection without neoadjuvant treatment. After QC (“Materials and Methods”), 115 patients (with 61 matched normal samples) were included in this analysis. The majority of patients had adenocarcinoma histology (84%) and stage I/II disease (94%) that was well or moderately differentiated (60%). *KRAS* status was available for 102 patients, of whom the majority had a *KRAS* mutation. Sixty-one patients had HRD status available, of whom 3% were HRD positive. After a median of 19 months of follow-up, there were 92 and 99 events of recurrence and death (overall survival), respectively (Table 1; Supplementary Table S1).

**Table 1.** Clinical characteristics of the study cohort.

Clinical variable	Total	Alive	Dead	*P* value
	*N* = 115	*N* = 16 (14%)	*N* = 99 (86%)	
Age category	​	​	​	​
Less than or equal to 65 years	48 (42%)	12 (75%)	36 (36%)	**0.01**
More than 65 years	67 (58%)	4 (25%)	63 (64%)	
Gender	​	​	​	​
Female	55 (48%)	8 (50%)	47 (47%)	1
Male	60 (52%)	8 (50%)	52 (53%)	
Stage^a^	​	​	​	​
Stage I/II	108 (94%)	16 (100%)	92 (93%)	0.59
Stage III/IV	6 (5%)	0 (0%)	6 (6%)	
Missing	1 (<1%)	0 (0%)	1 (1%)	
Grade	​	​	​	​
Poorly differentiated	42 (37%)	6 (38%)	36 (36%)	1
Undifferentiated	4 (3%)	0 (0%)	4 (4%)	
Well/moderately differentiated	69 (60%)	10 (62%)	59 (60%)	
Tumor location	​	​	​	​
Body	8 (7%)	0 (0%)	8 (8%)	0.63
Head	85 (74%)	14 (88%)	71 (72%)	
Tail/other	21 (18%)	2 (12%)	19 (19%)	
Unknown	1 (<1%)	0 (0%)	1 (1%)	
Histology	​	​	​	​
Mucinous	9 (8%)	3 (19%)	6 (6%)	0.21
Others	9 (8%)	1 (6%)	8 (8%)	
PDA	97 (84%)	12 (75%)	85 (86%)	
Margin	​	​	​	​
Positive	37 (32%)	2 (12%)	35 (35%)	0.09
Negative	78 (68%)	14 (88%)	64 (65%)	
Recurrence	​	​	​	​
No recurrence	23 (20%)	12 (75%)	11 (11%)	**<0.001**
Recurrence	92 (80%)	4 (25%)	88 (89%)	
*KRAS* status	​	​	​	​
*KRAS* mutation	88 (77%)	9 (56%)	79 (80%)	**0.02**
No *KRAS* mutation	14 (12%)	5 (31%)	9 (9%)	
Unknown	13 (11%)	2 (12%)	11 (11%)	
HRD status	​	​	​	​
Negative	55 (48%)	8 (50%)	47 (47%)	0.98
Positive	4 (3%)	0 (0%)	4 (4%)	
Unknown	56 (49%)	8 (50%)	48 (48%)	

Bold values are those with a statistically significant *P* value < 0.05.

a Staging according to the eighth Edition of the American Joint Committee on Cancer.

### MS runs and proteomic analyses

**Figure 1.**
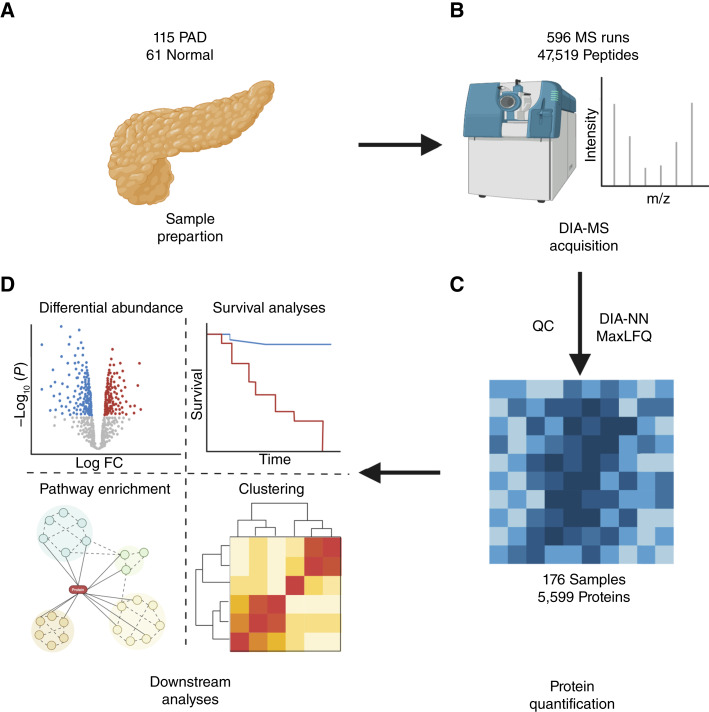
A schematic representing (**A**) data collection from 30-μm sections of 115 PDA and 61 adjacent normal fresh-frozen tissues that were prepared (19) for (**B**) DIA-MS. **C**, Protein quantification from DIA-MS data files using DIA-NN and MaxLFQ software for data normalization, QC, and peptide-to-protein inference and (**D**) the downstream analyses of the protein data. FC, fold change.

Proteomic data were generated from the 176 samples using DIA-MS in a total of 596 MS runs with 47,519 detected peptides. After performing QC, protein rollup, and tumor content consolidation, 5,599 proteins were detected (Fig. 1), with a mean missingness of 25% per sample.

### A diagnostic proteomic panel for pancreatic adenocarcinoma

**Figure 2.**
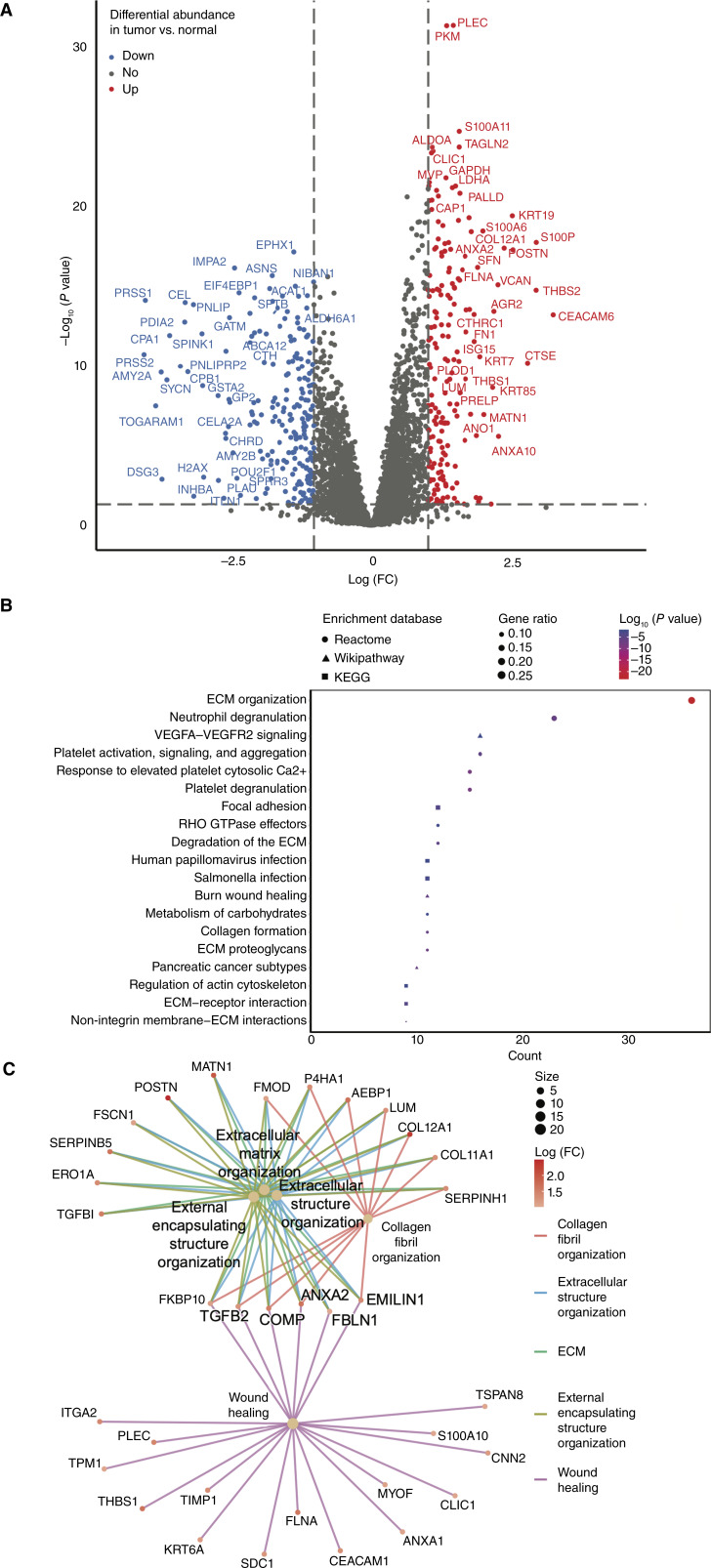
DAPs and dysregulated pathways in PDA tumors compared with adjacent normal tissue. **A,** DAPs (*n* = 395; *P* < 0.05) between PDA and adjacent normal tissue. **B,** Top enriched pathway proteins upregulated in PDA tissue. KEGG, Kyoto Encyclopedia of Genes and Genomes. **C,** Gene linkage network of enriched pathways from Gene Ontology biological processes database for upregulated proteins in PDA tumors. FC, fold change.

To understand the biological pathways associated with PDA development, DAA and PEA were performed, comparing tumor with adjacent normal samples (“Materials and Methods”). A total of 395 DAPs were identified, of which 176 were upregulated in tumor samples (Fig. 2A; Supplementary Data S1). The most prominent pathways associated with PDA were ECM, neutrophil degranulation, VEGF-related pathways (VEGFA–VEGFR2 signaling), platelet aggregation, and focal adhesion (Fig. 2B; Supplementary Data S2). Network analyses (Fig. 2C) showed an interaction between ECM, collagen fibril organization, and wound healing–related pathways. Five proteins were involved in all of these pathways (EMILIN1, ANXA2, TGFB2, COMP, and FKBP10), of which ANXA2 and FKBP10 may represent potential therapeutic targets as listed in the Human Protein Atlas (HPA) database (28).

Several proteins previously identified as potentially diagnostic for PDA (reviewed in ref. 29) were among the upregulated proteins in tumor samples in our cohort (LGALS1, ANXA2, LGALS3BP, and CTSD). In addition, among the top DAPs identified by Cao and colleagues (17), the following were also upregulated in our tumor samples: S100P, COL12A1, SFN, THBS2, CTHRC1, THBS1, SERPINB5, and LAMC2. Through combining these lists of common proteins with the top DAP in our analyses based on the lowest adjusted *P* value and highest fold change (Supplementary Data S1), a panel of 20 proteins (LGALS1, ANXA2, LGALS3BP, CTSD, S100P, COL12A1, SFN, THBS2, CTHRC1, THBS1, SERPINB5, LAMC2, POSTN, CEACAM6, CTSE, PLEC, PKM, S100A11, TAGLN2, and ALDOA) was shown to be diagnostic for pancreatic neoplasm according to an enrichment analysis in Metascape/DisGeNET pathways for disease diagnosis (Supplementary Fig. S1A; ref. 30). Of these 20 proteins, 19 (all except CTSE) can be detected in blood samples using MS as shown in the HPA database (28), thus highlighting their potential clinical utility, including for screening.

Through mapping the top DAP in PDA in our analyses with proteomic and drug response data from the SANGER cell lines (“Materials and Methods”; ref. 27), the following drugs and their corresponding target proteins were identified as potential therapeutic options: afatinib or PF-06747775 (targeting EGFR and ERBB2 pathways through CTSD and LGALS3BP), dabrafenib or PLX-4720 (targeting BRAF pathway through CTSD), and GSK429286A (targeting ROCK1 and ROCK2 pathways through LGALS3BP).

To assess whether tumor cellularity influenced the identification of the 20-protein diagnostic panel, we repeated the DAA using only samples with ≥50% tumor content. This analysis identified 285 proteins (Supplementary Data S1), of which 138 overlapped with the original, unadjusted list of 176 DAPs. Nineteen of the 20 diagnostic proteins (excluding THBS1) remained in the tumor content–adjusted DAP. Notably, 147 proteins were uniquely identified in the tumor content–adjusted DAP and were primarily associated with neutrophil degranulation, NOD-like receptor signaling, and angiogenesis signaling, based on Metascape enrichment analysis (Supplementary Fig. S1B; ref. 30).

Given that PDA characteristically has a strong desmoplastic reaction, we considered the consequences of excluding proteins associated with fibroblasts and collagen deposition, as annotated in the HPA (28), from the list of upregulated tumor DAPs. This subtraction yielded 112 proteins (of 176) likely specific to tumor cells rather than the stromal compartment (Supplementary Data S1). These proteins were enriched in pathways related to cytoskeletal organization, neutrophil degranulation, and metabolism (Metascape; Supplementary Fig. S1C; ref. 30). Of these, eight proteins overlapped with the 20-protein diagnostic panel (S100P, SFN, SERPINB5, CEACAM6, CTSE, S100A11, TAGLN2, and ALDOA). Although these eight proteins retained diagnostic relevance for PDA when analyzed in Metascape (30), their statistical significance was reduced, highlighting the substantial contribution of the desmoplastic reaction to the proteome of PDA.

### Proteomic-based classification of PDA

**Figure 3.**
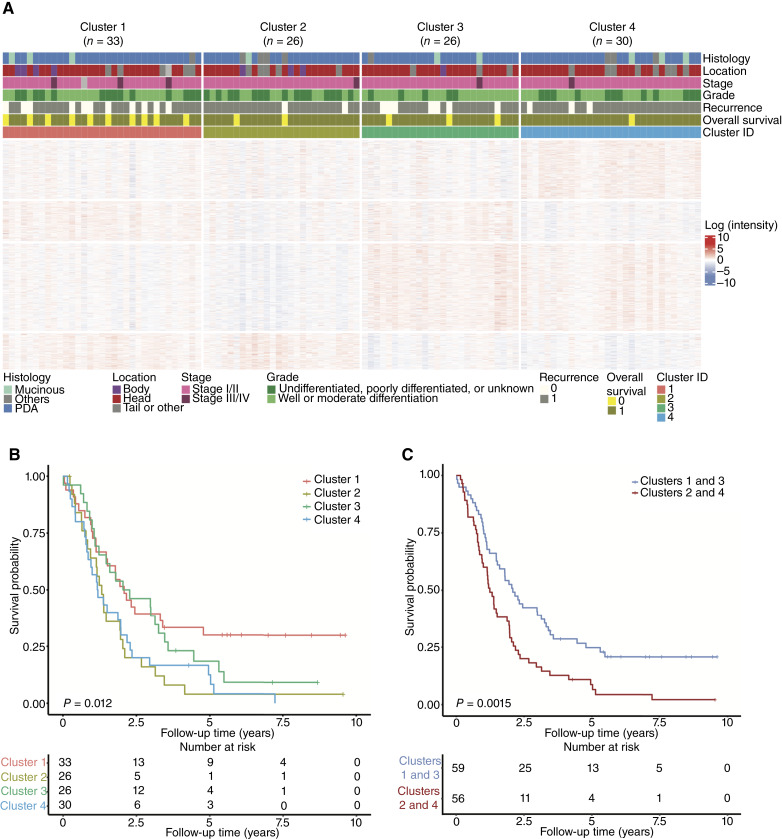
Proteomic subtypes of PDA. **A,** A clustered heatmap displaying the highly variable protein intensities across the four clusters detected within this cohort. **B,** Kaplan–Meier plots of the survival rates of the four clusters. **C,** Kaplan–Meier plot of the survival rates of the clusters combined according to prognosis (cluster 1 with 3 and cluster 2 with 4).

To explore proteomic-based subtypes of PDA, we performed unsupervised consensus clustering (“Materials and Methods”) on the tumor samples (*n* = 115) using the top 25% (*n* = 1,399) proteins with the highest MAD. Based on the visual inspection of the cumulative distribution function and silhouette width plots (Supplementary Fig. S2A and S2B), four clusters were identified (Fig. 3A). The four clusters showed highly variable protein intensities (Fig. 3A).

The clusters were associated with overall survival (*P* = 0.012; Fig. 3B). Clusters 2 and 4 had the worst prognosis in terms of overall survival (*P* = 0.0015; Fig. 3C) and recurrence-free survival (*P* < 0.001; Supplementary Fig. S2C). Clinical variables did not show significant differences among the four clusters, suggesting that the prognostic information in the proteomic clustering is independent of the clinical variables (Table 2; Supplementary Table S2).

**Table 2.** The distribution of clinical variables across the four clusters.

Clinical variable	Cluster 1	Cluster 2	Cluster 3	Cluster 4	*P* value
	*N* = 33	*N* = 26	*N* = 26	*N* = 30	
Age category	​	​	​	​	​
Less than 65 years	16 (48%)	12 (46%)	12 (46%)	8 (27%)	0.28
More than 65 years	17 (52%)	14 (54%)	14 (54%)	22 (73%)	
Gender	​	​	​	​	​
Female	16 (48%)	7 (27%)	14 (54%)	18 (60%)	0.09
Male	17 (52%)	19 (73%)	12 (46%)	12 (40%)	
Stage	​	​	​	​	​
Stage I/II	29 (88%)	25 (96%)	25 (96%)	29 (97%)	0.74
Stage III/IV	3 (9%)	1 (4%)	1 (4%)	1 (3%)	
Missing	1 (3%)	0 (0%)	0 (0%)	0 (0%)	
Grade	​	​	​	​	​
Poorly differentiated	12 (36%)	14 (54%)	4 (15%)	12 (40%)	**0.05**
Undifferentiated	2 (6%)	1 (4%)	1 (4%)	0 (0%)	
Well/moderately differentiated	19 (58%)	11 (42%)	21 (81%)	18 (60%)	
Histology	​	​	​	​	​
Mucinous	3 (9%)	1 (4%)	2 (8%)	3 (10%)	0.65
Others	1 (3%)	4 (15%)	1 (4%)	3 (10%)	
PDA	29 (88%)	21 (81%)	23 (88%)	24 (80%)	
Margin	​	​	​	​	​
Positive	12 (36%)	9 (35%)	9 (35%)	7 (23%)	0.7
Negative	21 (64%)	17 (65%)	17 (65%)	23 (77%)	
PNI	​	​	​	​	​
Absent	8 (24%)	3 (12%)	4 (15%)	6 (20%)	0.51
Present	23 (70%)	21 (81%)	22 (85%)	24 (80%)	
Unknown	2 (6%)	2 (8%)	0 (0%)	0 (0%)	
LVI	​	​	​	​	​
Absent	12 (36%)	7 (27%)	12 (46%)	14 (47%)	0.44
Present	19 (58%)	17 (65%)	14 (54%)	16 (53%)	
Unknown	2 (6%)	2 (8%)	0 (0%)	0 (0%)	
Recurrence	​	​	​	​	​
No recurrence	11 (33%)	2 (8%)	7 (27%)	3 (10%)	**0.04**
Recurrence	22 (67%)	24 (92%)	19 (73%)	27 (90%)	
Vital status	​	​	​	​	​
Alive - with disease	0 (0%)	0 (0%)	0 (0%)	1 (3%)	**0.004**
Alive - without disease	10 (30%)	2 (8%)	3 (12%)	0 (0%)	
Deceased - of disease	22 (67%)	24 (92%)	20 (77%)	27 (90%)	
Deceased - of other cause	1 (3%)	0 (0%)	3 (12%)	2 (7%)	
Overall survival (years)	​	​	​	​	​
Mean	3.18	1.75	2.73	1.87	0.06
SD	2.8	1.9	2.1	1.8	

Bold values are those with a statistically significant *P* value < 0.05.

Abbreviations: LVI, lymphovascular invasion; PNI, perineural invasion.

DAA and PEA were performed for each cluster versus the others (Supplementary Table S3; Supplementary Data S1 and S2). As patients in clusters 2 and 4 had similar prognoses (Fig. 3B), DAA and PEA were performed to identify the common upregulated biological pathways in these two clusters compared with the others. In this analysis, 153 DAPs were identified, of which 42 were upregulated in clusters 2 and 4 (Supplementary Fig. S3A; Supplementary Data S1), and the top DAPs included PFKP, ERO1A, ANXA3, S100P, COL12A1, SLC2A1, MATN1, HTRA3, THBS2, and AEBP1. The upregulated proteins among clusters 2 and 4 were associated with neutrophil degranulation, ECM, mesenchymal–epithelial transition, focal adhesion, and PI3K–Akt–mTOR signaling pathway (Supplementary Fig. S3B; Supplementary Data S2).

Among the top DAPs in cluster 2 and 4 (Supplementary Data S1), the following were identified as potential therapeutic targets (28): ANXA3, ELANE, FN1, MB, PRKC1, SLC2A1, AEBP1, HTRA1, PRTN3, and FGR. Moreover, SLC2A1 was also identified from the SANGER cell drug response data (27), being a target for AZD5582, SB590885, TGX221, and TL-2-105 molecules. SLC2A1 is a member of the SLC superfamily that is associated with PDA poor prognosis (31). This is consistent with our results, which show that SLC2A1 is associated with poorer survival in a univariate Cox regression model [HR, 1.28; 95% confidence interval (CI), 1.18–1.47].

### Protein-based risk score

**Figure 4.**
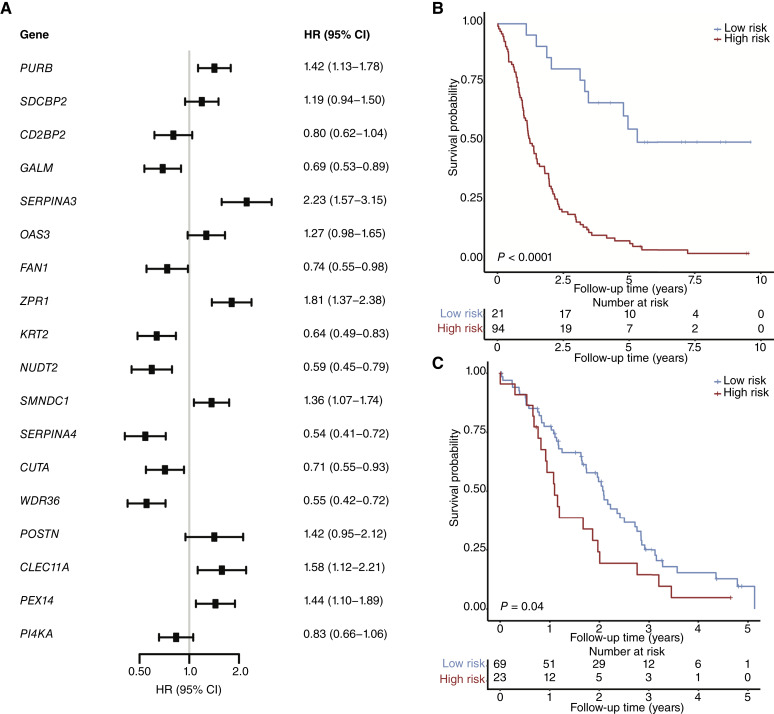
Proteomic risk score for mortality. **A,** Forest plot detailing the multivariable HRs for the overall survival of each of the proteins used to build the proteomic risk score. **B,** Kaplan–Meier curve displaying the overall survival probability of the low- and high-risk groups within our cohort. **C,** Kaplan–Meier curve displaying the survival probability of the low- and high-risk groups within the CPTAC validation dataset.

A protein-based risk score, developed by Cox regression analyses (“Materials and Methods”), was based on the expression levels of 18 proteins: PURB, SDCBP2, CD2BP2, GALM, SERPINA3, OAS3, FAN1, ZPR1, KRT2, NUDT2, SMNDC1, SERPINA4, CUTA, WDR36, POSTN, CLEC11A, PEX14, and PI4KA (Fig. 4A; Supplementary Table S4). The association of each of these proteins with overall survival is shown in Fig. 4A and Supplementary Fig. S4. These proteins had a mean of 10 peptides detected per protein and had a mean missingness of 6% (Supplementary Fig. S5).

Using the midpoint of the proteomic risk score as a threshold, we were able to significantly (*P* < 0.0001) dichotomize patients into low- and high-risk groups for mortality (Fig. 4B). The inclusion of the risk score as a covariate in the multivariable Cox regression model, alongside all prognostic clinical variables, significantly enhanced the concordance index (from 0.66 to 0.85). Furthermore, the statistical significance of the risk score was maintained even after adjusting for potential confounding clinical variables (Supplementary Fig. S6). Most of the clinical variables were well distributed across the low- and high-risk groups, consistent with the conclusion that the risk score is independent of the clinical variables (Table 3; Supplementary Table S5). Interestingly, there were more *KRAS* mutation cases in the high-risk group, yet no statistical interaction between the proteomic risk score and the *KRAS* status was observed (*P*_*interaction*_ = 0.063). The time-dependent ROC analyses showed that the proteomic risk score has the best area under the ROC (0.94; Supplementary Fig. S7A) that is stable over time compared with other prognostic clinical variables (Supplementary Fig. S7B). The 18-protein signature was also prognostic for 3-year overall survival (*P* < 0.001) and recurrence-free survival (*P* < 0.001; Supplementary Fig. S8).

**Table 3.** Clinical variables across the proteomic-based risk groups.

Clinical variable	Proteomic risk score		*P* value
	High risk	Low risk	
	*N* = 94	*N* = 21	
Age category	​	​	​
Less than 65 years	35 (37%)	13 (62%)	**0.05**
More than 65 years	59 (63%)	8 (38%)	
Gender	​	​	​
Female	48 (51%)	7 (33%)	0.16
Male	46 (49%)	14 (67%)	
Stage	​	​	​
Stage I/II	87 (93%)	21 (100%)	0.59
Stage III/IV	6 (6%)	0 (0%)	
Missing	1 (1%)	0 (0%)	
Grade	​	​	​
Poorly differentiated	37 (39%)	5 (24%)	0.32
Undifferentiated/unknown	3 (3%)	1 (5%)	
Well/moderately differentiated	54 (57%)	15 (71%)	
Histology	​	​	​
Mucinous	5 (5%)	4 (19%)	0.11
Others	8 (9%)	1 (5%)	
PDA	81 (86%)	16 (76%)	
Margin	​	​	​
Positive	33 (35%)	4 (19%)	0.2
Negative	61 (65%)	17 (81%)	
PNI	​	​	​
Absent	15 (16%)	6 (29%)	0.36
Present	75 (80%)	15 (71%)	
Unknown	4 (4%)	0 (0%)	
LVI	​	​	​
Absent	32 (34%)	13 (62%)	0.07
Present	58 (62%)	8 (38%)	
Unknown	4 (4%)	0 (0%)	
Recurrence	​	​	​
No recurrence	14 (15%)	9 (43%)	**0.01**
Recurrence	80 (85%)	12 (57%)	
Vital status	​	​	​
Alive - with disease	0 (0%)	1 (5%)	**<0.001**
Alive - without disease	5 (5%)	10 (48%)	
Deceased - of disease	83 (88%)	10 (48%)	
Deceased - of other cause	6 (6%)	0 (0%)	

Bold values are those with a statistically significant *P* value < 0.05.

Abbreviations: LVI, lymphovascular invasion; PNI, perineural invasion.

The association of the risk score proteins with prognosis was consistent with data extracted from HPA (28) for 12 of the 18 proteins: CUTA, NUDT2, PI4K4, CD2BP2, FAN1, OAS3, POSTN, PURB, SDCBP2, SERPINA3, SMNDC1, and ZPR1 (Supplementary Table S4). Moreover, 10 of the 18 proteins have been detected in blood (28) and, as this may increase their utility as biomarkers, we checked whether a risk score built from these 10 proteins is prognostic. As shown in Supplementary Fig. S9, the risk score constructed from these 10 proteins was also prognostic for survival.

### Validating the proteomic risk score

We validated the proteomic risk score using an independent publicly available CPTAC PDA dataset generated using a different proteomic technology, tandem mass tag labeling (17). All 18 of the risk score proteins were identified in the CPTAC cohort, with a mean missingness of 4%. The risk score was recalculated from these 18 proteins (“Materials and Methods”) and showed a statistically significant association with overall survival (*P* = 0.04; Fig. 4C).

### The proteomic profiling of PDA based on molecular features

#### KRAS mutation

In our cohort, 88 patients had a *KRAS* mutation identified. The three most common mutations were *KRAS*^G12N^ (*n* = 39), *KRAS*^G12V^ (*n* = 24), and *KRAS*^G12R^ (*n* = 16). Only two patients had a *KRAS*^G12C^ mutation. *KRAS* mutation was not prognostic for survival or recurrence in our cohort. DAA revealed 72 DAPs (Supplementary Fig. S10A; Supplementary Data S1), of which 16 were upregulated in *KRAS*-mutant samples. These proteins were related to ECM structural, glycosaminoglycan, and sulfur- and carbohydrate-binding pathways (Supplementary Fig. S10B; Supplementary Data S2). The top DAPs included VCAN, SFRP4, MAN1C1, KRT19, CLEC11A, POSTN, PREX1, GJA1, FN1, and CEACAM7.

#### KRAS-mutant subgroups

The DAPs in each of the *KRAS*-mutant subgroups are listed in Supplementary Data S1. In our analyses, 292 proteins were differentially abundant in samples with *KRAS*^G12C^ (Supplementary Fig. S11A; Supplementary Data S1), of which 109 were upregulated. The top upregulated proteins were KRT14, CRAT, GAMT, KRT16, KRT5, DIAPH2, P4HB, MMAB, KRT15, PABPC3, HBG1, and MCCC1. The upregulated pathways were related to keratinization pathways, as well as other metabolic-related pathways, including cobalamin metabolism, urea cycle metabolism, pancreatic secretion, and protein digestion (Supplementary Fig. S11B; Supplementary Data S2). The following proteins were identified from the top DAPs as potential drug targets (28) in patients with *KRAS*^G12C^: EGFR, EPCAM, GAMT, PNLIP, CRAT, P4HB, MMAB, MCCC1, SARDH, HSPA5, and CIT. When mapping the DAPs in patients with *KRAS*^G12C^ with the SANGER drug response data (27), the following were identified as potential drug targets (with their corresponding target proteins): afatinib (KRT6A, KRT5, and KRT16), PF-06747775 (KRT5 and SA100A2), AZD5582 (P4HB), venetoclax (P4HB), and WEHI-539 (NUCB2). Recent data showed some promising results of targeting *KRAS*^G12C^ in lung and PDA; however, intrinsic and acquired resistance were common, mostly through PI3K skip signaling, among other mechanisms (32, 33). Our results should be interpreted with caution, considering the small number of patients with *KRAS*^G12C^ mutations; further validation of our findings is required.

*KRAS* ^G12N^ was the most frequently identified *KRAS* mutation in our cohort. DAA revealed only 27 DAPs (Supplementary Data S1; Supplementary Fig. S12), of which 11 were upregulated in *KRAS*^G12N^ (PKLR, APOC4, DMKN, KLDHC7B, SMPX, CKM, CAPNS2, ANXA8L1, TNN, HSPB7, and ACTG2). Of these, CKM and PKLR were identified as potential drug targets (28).

#### HRD status

HRD is a predictive biomarker for response to platinum-based chemotherapy and PARP inhibitors in several types of cancers, including PDA (34). In our cohort, HRD-positive tumors were associated with poor survival (HR, 7.4; 95% CI, 2.5–22). Despite the small sample size (*n* = 4 HRD positive; *n* = 55 HRD negative), 205 and 95 proteins were upregulated and downregulated, respectively, based on the HRD-positive status (Supplementary Fig. S13A; Supplementary Data S1). The top 10 upregulated proteins in HRD-positive cases were FCGR3A, MOB3A, GCA, STOM, NCF2, ITGAM, CD14, PYGL, MNDA, and STOML3. The upregulated proteins were mainly associated with immune-related pathways (neutrophil degranulation, complement system, immune response, inflammatory response, and neutrophil extracellular trap formation), hemostasis, and cell–cell adhesion (Supplementary Fig. S13B; Supplementary Data S2). This is consistent with the known relationship between HRD and inflammation (reviewed in ref. 35).

Among these top DAPs, GCA, NCF2, ITGAM, PYGL, and MNDA were associated with poorer survival (HR, 1.3; *P* < 0.05), with ITGAM having the strongest effect (HR, 1.4; *P* < 0.001). Data extracted from the HPA (28) showed that PYGL and NCF2 are associated with poor survival in PDA. ITGAM is an immune-related gene related to an increased risk of tumor metastasis and poorer survival (36–38). However, its role in PDA is not fully explored.

Several potential therapeutic targets (28) were identified among the top upregulated proteins in patients with HRD-positive tumors, which included FCGR3A, F10, IMPDH1, ELANE, BST1, ALOX5, MMP9, FGB, PYGL, GYS1, MOCS2, G6PD, SOD2, GYG1, HABP2, PLOD1, PRTN3, RAPB27A, DYSF, CYBB, STK10, and PTPRC. When mapping these results with the SANGER drug response data (27), several proteins were identified which were therapeutic targets for afatinib (KRT16, KRT5, KRT6A, and S100A2), AZD4547 (DSG3), PF-06747775 (KRT5), and savolitinib (NDRG1).

#### COSMIC mutational signatures

Sixty-one patients in our cohort had information available on the COSMIC mutational signatures (39). All 61 patient samples showed somatic mutations consistent with COSMIC signature 1. In a univariate Cox regression analysis, and among all the COSMIC signatures, only COSMIC Sig3 (HR, 3.34; 95% CI, 1.56–7.15) was associated with poorer survival. In a multivariate Cox regression model, including all of the COSMIC signatures with Step-Akaike information criterion feature selection, five COSMIC signatures were identified, of which only two were significantly associated with survival (COSMIC Sig2 and Sig3; Supplementary Table S6).

We performed DAA for each of the COSMIC signatures (Supplementary Data S2) and highlighted the related upregulated pathways (Supplementary Table S7). COSMIC Sig3 (9% prevalence in our cohort), which is mostly associated with *BRCA* mutations, was of interest to this analysis because of its occurrence in PDA (40). Eighty-five proteins were upregulated in patients with COSMIC Sig3 mutation (Supplementary Fig. S14A; Supplementary Data S1). These proteins were associated with ECM and immune response–related pathways (Supplementary Fig. S14B; Supplementary Data S2), consistent with the finding for HRD. Mapping the list of upregulated proteins in Sig3 against SANGER pan-cancer drug response data (27) revealed possible therapeutic targets for afatinib (KRT16, KRT17, KRT5, KRT6A, and S100A2), AZD5582 (SLC2A1), crizotinib (KRT17), PF-06747775 (KRT17, KRT5, and S100A2), SB590885 (SLC2A1), and TGX221 (SLC2A1).

### S100 family proteins

Seventeen S100 proteins were identified in our protein matrix (Supplementary Table S8), of which S100P, S100A4, S100A6, S100A10, and S100A11 were upregulated in tumor samples. This is consistent with other studies (41), which showed the upregulation of S100A4, S100A6, S100A10, and S100A11 in tumor samples. Interestingly, S100A2, S100A8, and S100A9 were upregulated in high-grade tumors in patients with HRD and patients with COSMIC Sig3. Univariate Cox regression models, each including one of the S100 family proteins, showed that S100A2, S100A11, S100A16, and S100A12 were associated with poor prognosis, whereas S100B was associated with good prognosis, suggesting possible prognostic value (Supplementary Fig. S15).

### KRT family proteins

Of the 54 known KRT proteins identified in human tissue, we were able to identify 29 in our PDA protein matrix (Supplementary Table S9). KRT6A, KRT7, KRT17, KRT19, KRT72, and KRT85 were upregulated in tumor samples (vs. matched normal samples), suggesting the diagnostic utility of these proteins. Data extracted from a study by Jiang and colleagues (41) showed that KRT7, KRT19, and KRT85 were among the upregulated KRT family proteins in tumor samples. In addition, KRT6A, KRT7, KRT17, KRT18, KRT19, and KRT85 were associated with poorer survival in our data, whereas KRT2, KRT77, and KRT9 were associated with better prognosis (Supplementary Fig. S16). This is consistent with previous publications, which showed the prognostic value of KRT6A and KRT17 (42–44). KRT7 was found to be also upregulated in patients who developed recurrence or died from pancreatic cancer in our data (Supplementary Data S1), denoting association with a poorer prognosis, consistent with previous findings (45). Similar to the findings observed in the S100 family, high grade, HRD, and COSMIC Sig3 showed similar upregulation of some of the KRT proteins, including KRT6A, KRT16, and KRT17 (Supplementary Table S9).

## Discussion

Pancreatic cancer is an aggressive disease with a heterogeneous tumor microenvironment and lacks robust biomarkers for guiding treatment. This limitation has hindered the effectiveness of targeted therapy and immunotherapies in PDA management. Previous studies have employed multiomic approaches to unravel the biological pathways and develop novel classifications associated with PDA (17, 18, 29, 41, 46). Our work extends this existing knowledge by providing new prognostic associations for PDA.

We conducted a proteomic analysis on tumors and matched normal samples from 115 patients in the APGI cohort, identifying a panel of 20 potentially diagnostic proteins. Notably, 19 of these proteins are detectable in blood, suggesting their utility in screening. Further validation is required to confirm their diagnostic and clinical utility. Our findings also align with those of Jiang and colleagues (41) regarding the biological pathways associated with PDA, including ECM organization, focal adhesion, and immune response. Moreover, our list of top DAPs partially overlaps with previous studies, which reported positive immunostaining, reflecting upregulation of S100P, KRT7, and KRT19, and negative staining, consistent with downregulation of trypsin (PRSS1), chymotrypsin (CTRC), and lipase (PNLIPRP1; ref. 47).

Among the top five DAPs we identified in tumor tissue (PLEC, PKM, S100A11, TAGLN2, and ALDOA), PLEC is a cancer-related protein that is involved in cancer cell proliferation, migration, and invasion and has been found to be prognostic in PDA (48). PKM is a cancer-related protein that is also prognostic in PDA (49) and is associated with hypoxia-related pathways (HIFA; refs. 46, 50), as well as with resistance to chemotherapy (51). S100A11 is a member of the S100 family involved in several cellular processes (52) and is associated with poor prognosis in PDA through its interaction with SMAD2/3 phosphorylation and regulation of transketolase expression and the pentose phosphate pathway, which supports rapid cellular proliferation (53, 54). TAGLN2 is known to be overexpressed in PDA (55), with a possible association with *KRAS* mutation through a complex pathway involving the ERK2 protein (55). Finally, ALDOA is a cancer-related protein and a potential drug target associated with poor prognosis in PDA through its interaction with E-cadherin, TGF-β1, and the HIFA pathways (56, 57).

We identified four proteomic-based subtypes of PDA with prognostic value and different biological profiles. The poorer prognostic subtypes showed upregulation in the immune-related, keratinization, and collagen formation pathways, suggesting a higher percentage of basal-like and activated stromal subtypes (15) within these clusters. These results suggest that the proteomic subtype classification could help stratify patients for immunotherapy, addressing a critical unmet need in this field (12).

Additionally, we constructed a proteomic-based risk score comprised of 18 proteins, many of which are recognized for their prognostic value in PDA, thus augmenting their clinical relevance. Importantly, based on this risk score, patients categorized in the low-risk group exhibit more promising prognoses, with a median survival exceeding 5 years. In addition, 10 of these proteins can be detected in blood using MS, which may enable their future clinical use. Our risk score underwent external *in silico* validation using a publicly available database, affirming the robustness of our findings and highlighting the necessity for further clinical validation.

Unsurprisingly, there is very little overlap between the proteins that distinguish PDA from normal tissue and those that predict which tumors are more aggressive than others; only one protein, POSTN (periostin), was shared between the diagnostic panel and the proteomic risk score. Periostin is a secreted ECM protein that has been shown to enhance the diagnostic accuracy of CA19-9 for detecting PDA (58). It has also been reported to be involved in PDA progression by promoting tumor cell motility, inducing epithelial-to-mesenchymal transition and activating the AKT signaling pathway (59).

Our results identified VCAN as the top DAP among patients with *KRAS* mutation, and others have identified it as the top DAP in PDA tumor samples (41). VCAN is a member of the proteoglycan family and is known to be overexpressed in *KRAS*-mutant cancer cells and to act as a mediator for KRAS-dependent activation of macrophages via IKKβ (60). It is associated with poor prognosis in PDA (61), as well as resistance to immunotherapy and chemotherapy, possibly through promoting tissue stiffness at the microenvironment level and alteration of immune cell phenotype (through activation of TLR2, TLR6, and CD14) and immune cell trafficking, making it a potential therapeutic target to overcome immune resistance (62, 63).

The S100 family proteins are highly expressed in several tissues and are involved in several cellular functions, including calcium homeostasis, inflammation, differentiation, immune response, apoptosis, and drug resistance (reviewed in ref. 64). Several S100 proteins were known to be of prognostic or diagnostic value in PDA, including S100A2, S100A4, S100A6, and S100A11 (reviewed in ref. 64). Similarly, several keratins have been identified as diagnostic markers for various types of cancers (reviewed in refs. 65, 66). We have shown a pattern of similar protein abundance among some of the KRT and S100 family proteins in HRD-positive, COSMIC Sig3, and high-grade tumors. Mapping the S100 and KRT family proteins in our data has revealed several prognostic proteins, including S100A2, S100A12, KRT6A, KRT7, KRT17, and KRT18. In addition, cluster 4 (the worst prognostic group) showed upregulation of S100P, S100A8, S100A9, KRT8, and KRT19 proteins. These findings are consistent with previous findings with regard to the association of these proteins with poor prognosis in PDA (17, 67, 68), making them good candidates as prognostic and potentially therapeutic biomarkers.

Through this work, we were able to map the proteomic profile of several COSMIC signatures within PDA and demonstrate that COSMIC Sig3 (associated with BRCA1 and BRCA2 mutations, important causes of HRD) and HRD overlap in their association with immune-related pathways and specifically the upregulation of FPR1 and SA100A12. Although the overactivation of immune-related pathways is associated with poorer prognosis in PDA, these subgroups may be good candidates for immunotherapy. COSMIC signatures 2, 8, and 18 also showed an association with poorer prognosis and, of these, signatures 8 and 18 might also be linked to DNA repair deficiencies (69).

Our study has some limitations. First, in the validation cohort, the survival difference between the high- and low-risk groups was smaller compared with the discovery cohort. This discrepancy may be partially explained by the use of a different proteomic technology for protein quantification. Second, despite the overall size of the cohort, the subgroups, mainly the HRD subgroup, *KRAS*^G12C^, and those with the different COSMIC signatures, were small. Third, six of the proteins in our risk score did not show a similar prognostic direction as indicated in the HPA (28). However, this may be partially explained by the fact that HPA data are based on gene and transcriptomic expression, which, on average, show low-to-moderate correlation with proteomic data (27). Finally, the cohort was restricted to patients who underwent surgical resection and may not be representative of all patients with PDA who predominantly present with metastatic disease.

Strengths include the availability of adjacent normal samples, which allowed identification of the top DAPs and pathways associated with PDA and of a diagnostic panel that may potentially be useful for PDA screening. Second, we have used the SANGER proteomic and drug response data to identify several potential therapeutic targets in PDA subgroups. Third, we have been able to identify proteomic-based subgroups that have differential prognoses. Finally, we have developed a proteomic-based risk score from 18 proteins that is predictive for overall survival, 3-year survival, and recurrence, which was validated in an independent PDA proteomic dataset generated with a different proteomic technology.

## Supplementary Material

List of APGI Researchers List of APGI researchers

Supplementary Table 1 Supplementary Table 1 shows additional clinical characteristics of the study cohort. Gln61Arg: Glutamine to Arginine at position 61, Gln61His: Glutamine to Histidine at position 61, Gly12Ala: Glycine to Alanine at position 12, Gly12Arg: Glycine to Arginine at position 12, Gly12Asp: Glycine to Aspartic Acid at position 12, Gly12Cys: Glycine to Cysteine at position 12, Gly12Leu: Glycine to Leucine at position 12, Gly12Val: Glycine to Valine at position 12

Supplementary Table 2 Supplementary Table 2 shows the distribution of the clinical variables across the four proteomic-based clusters. Gln61Arg: Glutamine to Arginine at position 61, Gln61His: Glutamine to Histidine at position 61, Gly12Ala: Glycine to Alanine at position 12, Gly12Arg: Glycine to Arginine at position 12, Gly12Asp: Glycine to Aspartic Acid at position 12, Gly12Cys: Glycine to Cysteine at position 12, Gly12Leu: Glycine to Leucine at position 12, Gly12Val: Glycine to Valine at position 12. Note that all patients showed positivity for COSMIC signature 1, while no patients showed positivity for COSMIC signatures 6, 20, 25, and 26. For COSMIC signatures 13, 18, 28, and 30, only one patient was in the positive group.

Supplementary Table 3 Supplementary Table 3 shows the differentially abundant proteins, their associated pathways, and the potential drug targets identified across the four proteomic-based clusters.

Supplementary Table 4 Supplementary Table 4 shows previous evidence regarding each protein included in the risk score in terms of their association with different types of cancer diagnosis and prognosis. *Based on data extracted from the Human Protein Atlas (https://www.proteinatlas.org/)

Supplementary Table 5 Supplementary Table 5 shows the distribution of clinical variables in the study cohort across the two proteomic-based risk score groups. Gln61Arg: Glutamine to Arginine at position 61, Gln61His: Glutamine to Histidine at position 61, Gly12Ala: Glycine to Alanine at position 12, Gly12Arg: Glycine to Arginine at position 12, Gly12Asp: Glycine to Aspartic Acid at position 12, Gly12Cys: Glycine to Cysteine at position 12, Gly12Leu: Glycine to Leucine at position 12, Gly12Val: Glycine to Valine at position 12. Note that all patients showed positivity for COSMIC signature 1, while no patients showed positivity for COSMIC signatures 6, 20, 25, and 26. For COSMIC signatures 13, 18, 28, and 30, only one patient was in the positive group.

Supplementary Table 6 Supplementary Table 6 shows the results of the univariate and multivariate Cox regression model with Stepwise-AIC, showing the association of each of the COSMIC signatures with overall survival in the study cohort. Note that all patients showed positivity for COSMIC signature 1, while no patients showed positivity for COSMIC signatures 6, 20, 25, and 26. For COSMIC signatures 13, 18, 28, and 30, only one patient was in the positive group.

Supplementary Table 7 Supplementary Table 7 shows the list of differentially abundant proteins (DAP) and their associated biological pathways, as well as the potential drug targets among each COSMIC signature of interest. Note that no patients showed positivity for COSMIC signatures 1,6,20,25,26, while COSMIC signatures 13, 18, 28, and 30 had only one patient in the positive group.

Supplementary Table 8 Supplementary Table 8 shows the differential abundance of the S100 protein family across the different clinical variables of interest in the study cohort, where “Up” means upregulated and “Down” means downregulated in the group of interest versus the other group. *Note that S100 A1, S100A13, and S100A3 were not differentially abundant among any subgroup.

Supplementary Table 9 Supplementary Table 9 shows the differential abundance of the KRT protein family across the different clinical variables of interest, where “Up” means upregulated and “Down” means downregulated in the group of interest versus others. *Note that KRT9, KRT10, KRT20, KRT23, KRT77, KRT74, KRT79 and KRT80 were not differentially abundant among any subgroup

Supplementary Data 1 List of the different differentially abundant proteins for the different groups of interest

Supplementary Data 2 List of the enriched pathways among the different groups of interest

Supplementary Figure 1 Supplementary Figure 1 shows the main pathways associated with lists of proteins of interest. (A) Summary of Enrichment analysis in DisGeNET for the 20-protein panel, showing a highly significant association with pancreatic neoplasm. (B) Summary of the main pathways associated with proteins uniquely identified when restricting the differential abundance analysis to samples with ≥ 50% cancer content. (C) Summary of the main pathways associated with proteins uniquely identified to be related to pancreatic tumor cells after subtracting the list of proteins associated with fibroblasts and collagen deposition. All the figure was generated from the Metascape website: (https://metascape.org/gp/index.html#/main/step1).

Supplementary Figure 2 Supplementary Figure 2 shows figures related to the Consensus clustering analyses. (A) A delta area plot displays the relative change in the cumulative distribution function (CDF) curve comparing k and k-1 clusters from our cohort. (B) The silhouette plot displays the silhouette width for the four clusters determined by consensus clustering within this cohort. (C) Kaplan-Meier plot of the recurrence-free survival rates of the combined clusters (Clusters 1 with 3 and Clusters 2 with 4).

Supplementary Figure 3 Supplementary Figure 3 shows differential abundance and pathway enrichment analyses of clusters. (A) Volcano plot displaying the differentially expressed proteins between PDA samples classified between groups 1 and 3 against groups 2 and 4. (B) Pathways enriched in KEGG, Reactome, and Wikipathways for proteins upregulated in samples from clusters 2 and 4 compared to clusters 1 and 3.

Supplementary Figure 4 Supplementary Figure 4 shows the Kaplan-Meier survival curves for each of the 18 proteins within the risk score. Median cut-off was used to dichotomize patients into two groups.

Supplementary Figure 5 Supplementary Figure 5 shows the proteomic information for the 18 risk-score proteins. (A) Peptide detection rate within the dataset. (B) Protein Missingness in our tumor data only. (C) Protein Missingness in the CPTAC Pancreatic Cancer Cohort.

Supplementary Figure 6 Supplementary Figure 6 shows a forest plot detailing the hazard ratio of the proteomic risk score and clinically relevant variables for PDA within our study cohort using multivariable Cox regression modeling.

Supplementary Figure 7 Supplementary Figure 7 shows the proteomic signature performance based on the Receiver operator characteristic curve analysis. (A) Receiver operator characteristic curve (ROC) at 1 year of follow-up for the proteomic signature (purple) and other clinically relevant variables for PDA within our cohort. (B) An Area Under the Curve (AUC) plot displays the AUC as a function of time for the proteomic risk score (purple) and other clinically relevant variables for PDA within our cohort.

Supplementary Figure 8 Supplementary Figure 8 shows Kaplan-Meier curves displaying (A) the three-year survival for groups dichotomised by proteomic risk score in ProCan data. (B) The recurrence-free survival for groups dichotomized by proteomic risk score in ProCan data.

Supplementary Figure 9 Supplementary Figure 9: Kaplan-Meier plot for patients dichotomized by a proteomic risk score that uses only the ten proteins detected in blood by mass spectrometry (PURB, GALM, SERPINA3, OAS3, KRT2, NUDT2, SERPINA4, CUTA, POSTN, CLEC11A).

Supplementary Figure 10 Supplementary Figure 10 shows the differential abundance and pathway enrichment analyses based on KRAS mutations. (A) Volcano plot displaying the differentially abundant proteins between KRAS mutant PDA and KRAS wild-type PDA. (B) Pathways enriched in Gene Ontology molecular function database from upregulated proteins in tumors that harbored KRAS mutations.

Supplementary Figure 11 Supplementary Figure 11 shows the differential abundance and pathway enrichment analyses based on the KRAS-G12C status. (A) Volcano plot displaying the differentially abundant proteins between tumors harboring the KRAS-G12C mutation versus any other KRAS-G12 mutation. (B) Pathways enriched in the KEGG, Reactome, and WikiPathways databases based on the upregulated proteins from tumors that harbored KRAS-G12C mutations when compared to tumors with other KRAS12 mutations.

Supplementary Figure 12 Supplementary Figure 12 shows a volcano plot displaying the differentially abundant proteins between tumors harboring a KRAS-G12D mutation versus those with any other KRAS-G12 mutation

Supplementary Figure 13 Supplementary Figure 13 shows the differential abundance and pathway enrichment analyses based on HRD status. (A) Volcano plot displaying the differentially abundant proteins between tumor samples with and without HRD. (B) Pathways enriched in KEGG, Reactome, and Wikipathways databases based on the upregulated proteins in tumors from patients with HRD-positive status.

Supplementary Figure 14 Supplementary Figure 14 shows the differential abundance and pathway enrichment analyses based on COSMIC Signature-3 status. (A) Volcano plot displaying the differentially abundant proteins between tumors with and without somatic mutations consistent with COSMIC Signature-3. (B) Pathways enriched in Reactome from upregulated proteins in tumors that displayed mutations corresponding to COSMIC Signature-3.

Supplementary Figure 15 Supplementary Figure 15 shows a Forest plot presentation of the hazard ratios for each S100 protein detected in the tumor samples from the study cohort, derived from a univariate Cox regression analysis with overall survival as the outcome of interest.

Supplementary Figure 16 Supplementary Figure 16 shows a Forest plot presentation of the hazard ratios for each KRT protein detected in the tumor samples from the study cohort, derived from a univariate Cox regression analysis with overall survival as the outcome of interest. Note that this figure excludes KRT75, which was detected in only two samples.

## Data Availability

The proteomic data in this study are publicly available in the Proteomics Identifications Database (https://www.ebi.ac.uk/pride/) with identifier PXD059074. Other data generated in this study are available upon request from the corresponding author.
