# Supplementary material for: Mapping the Proteomic Landscape of Pancreatic Cancer: Prognostic Insights and Subtype Stratification
Source: Cancer Res Commun. 2025 Oct 23;5(10):1879–93. doi: 10.1158/2767-9764.CRC-25-0229 (PMC12548992; doi:10.1158/2767-9764.CRC-25-0229)
Supplement: List of APGI Researchers [file crc-25-0229_list_of_apgi_researchers_suppapgi-1.docx]

# Supplementary Tables and Figures

# **Australian Pancreatic Cancer Genome Initiative (APGI)**

# **Contact APGI:** [anthony.james.gill@sydney.edu.au](mailto:anthony.james.gill@sydney.edu.au); [research@pancreaticcancer.net.au](mailto:research@pancreaticcancer.net.au)

# **List of APGI researchers**

**Garvan Institute of Medical Research** Amber L. Johns^1^, Anthony J Gill^1,5^, Lorraine A. Chantrill^1,22^, Paul Timpson^1,^ Angela Chou^1,5^, Marina Pajic^1^, Tanya Dwarte^1^, David Herrmann^1^, Claire Vennin^1^, Thomas R Cox^1^, Brooke Pereira^1^, Shona Ritchie^1^, Daniel A Reed^1^, Cecilia R Chambers^1^, Max Nobis^1^, Gloria Jeong^1^, Ruth J. Lyons^1^, Nicola Blackburn^1^, Adnan Nagrial^1^, Sean Porazinski^1^, Diego Chacon Fajardo^1^, Alice Russo^1^. **QIMR Berghofer Medical Research Institute** Nicola Waddell^2^, John V. Pearson^2^, Katia Nones^2^, Felicity Newell^2^, Venkateswar Addala^2^, Oliver Holmes^2^, Conrad Leonard^2^, Scott Wood^2^. **University of Melbourne, Centre for Cancer Research** Sean M. Grimmond^3^, Oliver Hofmann^3^. **Royal North Shore Hospital** Jaswinder S. Samra^5^, Nick Pavlakis^5,^ Jennifer Arena^5^, Hilda A. High^5^, Anubhav Mittal^5^. **Bankstown Hospital** Ray Asghari^6^, Neil D. Merrett^6^, Amitabha Das^6^. **Liverpool Hospital** Peter H. Cosman^7^, Kasim Ismail^7^. **St Vincent’s Hospital** Alina Stoita^8^, David Williams^8^, Allan Spigellman^8^**. Westmead Hospital** Duncan McLeod^9^, Judy Kirk^9^. **Royal Prince Alfred Hospital, Chris O’Brien Lifehouse** James G. Kench^10^, Peter Grimison^10^, Charbel Sandroussi^10^, Annabel Goodwin^7,10^. **Prince of Wales Hospital** R. Scott Mead^1,11^, Katherine Tucker^11^, Lesley Andrews^11^. **Fiona Stanley Hospital** Michael Texler^12^, Cindy Forrest^12^, Mo Ballal^12,13^, David Fletcher^12^**. St John of God Healthcare** Maria Beilin^13^, Kynan Feeney^13^ Krishna Epari^13^ Sanjay Mukhedkar^13^. **Epworth HealthCare** Nikolajs Zeps^23^. **Royal Adelaide Hospital** Nan Q Nguyen^14^, Andrew R. Ruszkiewicz^14^, Chris Worthley^14^. **Flinders Medical Centre** John Chen^15^, Mark E. Brooke-Smith^15^, Virginia Papangelis^15^. **Envoi Pathology** Andrew D. Clouston^16^. **Princess Alexandra Hospital** Andrew P. Barbour^17^, Thomas J. O’Rourke^17^, Jonathan W. Fawcett^17^, Kellee Slater^17^, Michael Hatzifotis^17^, Peter Hodgkinson^17^. **Austin Hospital** Mehrdad Nikfarjam^18^. **Johns Hopkins Medical Institutes** James R. Eshleman^19^, Ralph H. Hruban^19^, Christopher L. Wolfgang^19^. **ARC-Net Centre for Applied Research on Cancer** Aldo Scarpa^20^, Rita T. Lawlor^20^, Vincenzo Corbo^20^, Claudio Bassi^20^. **University of Glasgow** Andrew V Biankin ^21^, Nigel B. Jamieson^21^ David K. Chang^1, 21,^ Stephan B. Dreyer^21^.

^1^The Kinghorn Cancer Centre, Garvan Institute of Medical Research, 370 Victoria Street, Darlinghurst, Sydney, New South Wales 2010, Australia.

^2^QIMR Berghofer Medical Research Institute, 300 Herston Rd, Herston, Queensland 4006, Australia.

^3^University of Melbourne, Centre for Cancer Research, Victorian Comprehensive Cancer Centre, 305 Grattan Street, Melbourne, Victoria 3000, Australia.

^4^ Institute for Molecular Bioscience, University of QLD, St Lucia, Queensland 4072, Australia.

^5^Royal North Shore Hospital, Westbourne Street, St Leonards, New South Wales 2065, Australia.

^6^Bankstown Hospital, Eldridge Road, Bankstown, New South Wales 2200, Australia.

^7^Liverpool Hospital, Elizabeth Street, Liverpool, New South Wales 2170, Australia.

^8^ St Vincent’s Hospital, 390 Victoria Street, Darlinghurst, New South Wales, 2010 Australia.

^9^Westmead Hospital, Hawkesbury and Darcy Roads, Westmead, New South Wales 2145, Australia.

^10^Royal Prince Alfred Hospital, Missenden Road, Camperdown, New South Wales 2050, Australia.

^11^Prince of Wales Hospital, Barker Street, Randwick, New South Wales 2031, Australia.

^12^Fiona Stanley Hospital, 11 Robin Warren Dr, Murdoch WA 6150

^13^ St John of God Healthcare, 12 Salvado Road, Subiaco, Western Australia 6008, Australia

^14^ Royal Adelaide Hospital, North Terrace, Adelaide, South Australia 5000, Australia.

^15^ Flinders Medical Centre, Flinders Drive, Bedford Park, South Australia 5042, Australia.

^16^ Envoi Pathology, 1/49 Butterfield Street, Herston, Queensland 4006, Australia.

^17^ Princess Alexandra Hospital, 199 Ipswich Rd, Woolloongabba QLD 4102

^18^ Austin Hospital, 145 Studley Road, Heidelberg, Victoria 3084, Australia.

^19^ Johns Hopkins Medical Institute, 600 North Wolfe Street, Baltimore, Maryland 21287, USA.

^20^ ARC-NET Center for Applied Research on Cancer, University of Verona, Via dell’Artigliere, 19 37129 Verona, Province of Verona, Italy.

^21^ Wolfson Wohl Cancer Research Centre, Institute of Cancer Sciences, University of Glasgow, Garscube Estate, Switchback Road, Bearsden, Glasgow, Scotland G61 1BD, United Kingdom.

^22^ Wollongong Hospital, Illawarra and Shoalhaven Local Health District, Loftus Street, Wollongong NSW 2500.

^23^ Epworth HealthCare, 89 Bridge Rd, Richmond VIC 3121, Australia

**Supplementary Table 1: Additional clinical characteristics of the study cohort**

| Clinical variables | | Alive | Dead | Total | p-value |
| --- | --- | --- | --- | --- | --- |
|  |  | N = 16 | N = 99 | N = 115 |  |
| Perineural invasion (PNI) | Absent | 6 (38%) | 15 (15%) | 21 (18%) | 0.13 |
|  | Present | 10 (62%) | 80 (81%) | 90 (78%) |  |
|  | Unknown | 0 (0%) | 4 (4%) | 4 (4%) |  |
| Lymphovascular invasion (LVI) | Absent | 11 (69%) | 34 (34%) | 45 (39%) | **0.04** |
|  | Present | 5 (31%) | 61 (62%) | 66 (57%) |  |
|  | Unknown | 0 (0%) | 4 (4%) | 1 (4%) |  |
| COSMIC Signature 2 | Negative | 8 (50%) | 45 (45%) | 53 (46%) | 0.58 |
|  | Positive | 0 (0%) | 6 (6%) | 6 (5%) |  |
|  | Unknown | 8 (50%) | 48 (48%) | 56 (49%) |  |
| COSMIC Signature 3 | Negative | 7 (44%) | 42 (42%) | 49 (43%) | 1 |
|  | Positive | 1 (6%) | 9 (9%) | 10 (9%) |  |
|  | Unknown | 8 (50%) | 48 (48%) | 56 (49%) |  |
| COSMIC Signature 5 | Negative | 4 (25%) | 33 (33%) | 37 (32%) | 0.46 |
|  | Positive | 4 (25%) | 18 (18%) | 22 (19%) |  |
|  | Unknown | 8 (50%) | 48 (48%) | 56 (49%) |  |
| COSMIC Signature 8 | Negative | 4 (25%) | 15 (15%) | 19 (17%) | 0.42 |
|  | Positive | 4 (25%) | 36 (36%) | 40 (35%) |  |
|  | Unknown | 8 (50%) | 48 (48%) | 56 (49%) |  |
| COSMIC Signature 9 | Negative | 8 (50%) | 46 (46%) | 54 (47%) | 1 |
|  | Positive | 0 (0%) | 5 (5%) | 5 (4%) |  |
|  | Unknown | 8 (50%) | 48 (48%) | 56 (49%) |  |
| COSMIC Signature 16 | Negative | 6 (38%) | 40 (40%) | 46 (40%) | 1 |
|  | Positive | 2 (12%) | 11 (11%) | 13 (11%) |  |
|  | Unknown | 8 (50%) | 48 (48%) | 56 (49%) |  |
| COSMIC Signature 17 | Negative | 6 (38%) | 45 (45%) | 51 (44%) | 0.3 |
|  | Positive | 2 (12%) | 6 (6%) | 8 (7%) |  |
|  | Unknown | 8 (50%) | 48 (48%) | 56 (49%) |  |
| KRAS Subtypes | No Mutation | 7 (44%) | 20 (20%) | 27 (23%) | **0.03** |
|  | Gln61Arg | 1 (6%) | 0 (0%) | 1 (<1%) |  |
|  | Gln61His | 1 (6%) | 3 (3%) | 4 (3%) |  |
|  | Gly12Ala | 0 (0%) | 1 (1%) | 1 (<1%) |  |
|  | Gly12Arg | 0 (0%) | 16 (16%) | 16 (14%) |  |
|  | Gly12Asp | 3 (19%) | 36 (36%) | 39 (34%) |  |
|  | Gly12Cys | 1 (6%) | 1 (1%) | 2 (2%) |  |
|  | Gly12Leu | 0 (0%) | 1 (1%) | 1 (<1%) |  |
|  | Gly12Val | 3 (19%) | 21 (21%) | 24 (21%) |  |

Gln61Arg: Glutamine to Arginine at position 61, Gln61His: Glutamine to Histidine at position 61, Gly12Ala: Glycine to Alanine at position 12, Gly12Arg: Glycine to Arginine at position 12, Gly12Asp: Glycine to Aspartic Acid at position 12, Gly12Cys: Glycine to Cysteine at position 12, Gly12Leu: Glycine to Leucine at position 12, Gly12Val: Glycine to Valine at position 12

**Supplementary Table 2: The distribution of clinical variables across the 4 clusters**

| Clinical Variables | | Cluster 1 | Cluster 2 | Cluster 3 | Cluster 4 | p-value |
| --- | --- | --- | --- | --- | --- | --- |
|  |  | N = 33 | N = 26 | N = 26 | N = 30 |  |
| KRAS Status | KRAS Mutation Found | 22 (67%) | 19 (73%) | 21 (81%) | 26 (87%) | 0.8 |
|  | No KRAS Mutation Found | 5 (15%) | 3 (12%) | 2 (8%) | 4 (13%) |  |
|  | Unknown | 6 (18%) | 4 (15%) | 3 (12%) | 0 (0%) |  |
| HRD status | Negative | 9 (27%) | 15 (58%) | 12 (46%) | 19 (63%) | 0.68 |
|  | Positive | 0 (0%) | 2 (8%) | 0 (0%) | 2 (7%) |  |
|  | Unknown | 24 (73%) | 9 (35%) | 14 (54%) | 9 (30%) |  |
| COSMIC Signature 2 | Negative | 9 (27%) | 15 (58%) | 10 (38%) | 19 (63%) | 0.78 |
|  | Positive | 0 (0%) | 2 (8%) | 2 (8%) | 2 (7%) |  |
|  | Unknown | 24 (73%) | 9 (35%) | 14 (54%) | 9 (30%) |  |
| COSMIC Signature 3 | Negative | 9 (27%) | 14 (54%) | 12 (46%) | 14 (47%) | 0.04 |
|  | Positive | 0 (0%) | 3 (12%) | 0 (0%) | 7 (23%) |  |
|  | Unknown | 24 (73%) | 9 (35%) | 14 (54%) | 9 (30%) |  |
| COSMIC Signature 5 | Negative | 5 (15%) | 12 (46%) | 7 (27%) | 13 (43%) | 0.87 |
|  | Positive | 4 (12%) | 5 (19%) | 5 (19%) | 8 (27%) |  |
|  | Unknown | 24 (73%) | 9 (35%) | 14 (54%) | 9 (30%) |  |
| COSMIC Signature 8 | Negative | 1 (3%) | 2 (8%) | 8 (31%) | 8 (27%) | 0.01 |
|  | Positive | 8 (24%) | 15 (58%) | 4 (15%) | 13 (43%) |  |
|  | Unknown | 24 (73%) | 9 (35%) | 14 (54%) | 9 (30%) |  |
| COSMIC Signature 9 | Negative | 8 (24%) | 17 (65%) | 11 (42%) | 18 (60%) | 0.42 |
|  | Positive | 1 (3%) | 0 (0%) | 1 (4%) | 3 (10%) |  |
|  | Unknown | 24 (73%) | 9 (35%) | 14 (54%) | 9 (30%) |  |
| COSMIC Signature 16 | Negative | 7 (21%) | 13 (50%) | 8 (31%) | 18 (60%) | 0.63 |
|  | Positive | 2 (6%) | 4 (15%) | 4 (15%) | 3 (10%) |  |
|  | Unknown | 24 (73%) | 9 (35%) | 14 (54%) | 9 (30%) |  |
| COSMIC Signature 17 | Negative | 8 (24%) | 15 (58%) | 10 (38%) | 18 (60%) | 1 |
|  | Positive | 1 (3%) | 2 (8%) | 2 (8%) | 3 (10%) |  |
|  | Unknown | 24 (73%) | 9 (35%) | 14 (54%) | 9 (30%) |  |
| KRAS Subtypes | No Mutation | 11 (33%) | 7 (27%) | 5 (19%) | 4 (13%) | 0.7 |
|  | Gln61Arg | 1 (3%) | 0 (0%) | 0 (0%) | 0 (0%) |  |
|  | Gln61His | 2 (6%) | 1 (4%) | 1 (4%) | 0 (0%) |  |
|  | Gly12Ala | 0 (0%) | 1 (4%) | 0 (0%) | 0 (0%) |  |
|  | Gly12Arg | 3 (9%) | 3 (12%) | 6 (23%) | 4 (13%) |  |
|  | Gly12Asp | 10 (30%) | 9 (35%) | 7 (27%) | 13 (43%) |  |
|  | Gly12Cys | 0 (0%) | 1 (4%) | 1 (4%) | 0 (0%) |  |
|  | Gly12Leu | 0 (0%) | 0 (0%) | 0 (0%) | 1 (3%) |  |
|  | Gly12Val | 6 (18%) | 4 (15%) | 6 (23%) | 8 (27%) |  |

Gln61Arg: Glutamine to Arginine at position 61, Gln61His: Glutamine to Histidine at position 61, Gly12Ala: Glycine to Alanine at position 12, Gly12Arg: Glycine to Arginine at position 12, Gly12Asp: Glycine to Aspartic Acid at position 12, Gly12Cys: Glycine to Cysteine at position 12, Gly12Leu: Glycine to Leucine at position 12, Gly12Val: Glycine to Valine at position 12.

Note that all patients showed positivity for COSMIC signature 1, while no patients showed positivity for COSMIC signatures 6, 20, 25, and 26. For COSMIC signatures 13, 18, 28, and 30, only one patient was in the positive group.

**Supplementary Table 3: Differentially abundant proteins, pathways and potential therapeutic targets within each cluster.**

|  | Cluster 1 | Cluster 2 | Cluster 3 | Cluster 4 |
| --- | --- | --- | --- | --- |
| Upregulated differentially abundant proteins | ADH1B, FABP4, GNG2, S100B, F13A1, APOC3, SPON1, HSPA12A, APOB, PROCR, CRYAB, TMEM119, IGHG2, HBB, APOC2, FGA, HSPB6, AOC3, HBA1, FLT1, GZMK, COL14A1, OLFML1, LAMB2, HBD, PDE4D, CPA3, CA1, FGG, MAN1C1, OGN, FHL1, CD36, GPD1, SLC4A1, PAM, CMA1, IGHV2-70D, CPE, APCS, ZFPM2, TMEM41B, ELN, LPA, SOD3, TFR2, BCR, SCG5, SCGN, MTUS1, PCSK1N, THAP1, CACNA2D2, PTPRN, CADPS, DMRT2, C1RL, PLIN1, CDHR5, CHGA, CD38, TAGLN3, GCG, AFP, ANAPC7, ANK2, GUF1, ALOX15, DES, TMEM40 | SRPX2, AEBP1, VPS11, ANGPTL2, COL12A1, MFGE8, TGFBI, POSTN, THBS1, FN1, THBS2, FBLN2, FMOD, SERPINF1, EFEMP2, MXRA5, COL11A1, ECM1, EDIL3, COL8A1, CPB2, CLEC11A, MATN1, COMP, HTRA1, EFEMP1, LOX, BGN, LTBP1, FBLN1, CCDC80, HTRA3, ISLR, BRCA2, VTN, GREM1, COL24A1, C1QTNF5, C8G, OAF, CTHRC1, CDH13, MMP28, PRELP, MMP19, ASPN, LOXL3, GPX3, VCAN, C4orf54, CILP, APCS, FBN1, TNC, MAMDC2, INHBA, PLIN2, CDK12, MFAP4, LAMB3, COL11A2, LECT2, OLFML2B, SLC2A1, HBG1, CRP, KRT6B, HAX1, MRPS2, AGFG2, ANGPTL4, CEACAM6, LOXL2, PLXNB1, A2ML1 | ACAT1, PDCD4, ABAT, MCEE, GATM, PNLIP, PDIA2, RRBP1, CEL, CPA1, SYCN, NUCB2, PRSS2, CELA3A, MCCC1, IMPA2, FBXO44, SCLY, ECI2, PNLIPRP2, LRRC59, PSAT1, CPB1, CRAT, FAHD2A, ACSS1, CHDH, PRSS1, ALDH1L2, SPINK1, EIF4EBP1, ELAPOR1, ERP27, PBLD, CTRC, TOGARAM1, RNASEH2A, GP2, PIK3C3, AMY2A, FKBP11, RGN, PHGDH, SARDH, DAPK1, EPHX2, AMY2B, GSTA2, NOD1, HDAC7, LGALS2, CELA2A, CLPS, KLK1, ZFP69B, PLA2G4D, ASRGL1, CELA3B, GAMT, MYL4, KIF21B, CIT, EIF2D, TRAM1, SEC11C, GRB10, FAM174B, NME2P1, RGS22, CHGB, DPEP1, NRCAM, CTRL, CKB, TCEA3, CELA2B, HOGA1, HOOK1, TPST2, PKIG, CPE, PAIP2B, TMSB10, TMEM97, SERPINI2, PRR14L, CHGA, ACADL, LRATD2, MYO5B, ARSL, CPA5, UGT2B4, HMGCS2, GIP, PNLIPRP1, TIMM23, RTL1, GPT2, XIRP2, SLC43A1, PCF11, CKMT2, ZG16, KRT35, L1RE1, KPNA7, IGF2BP1, HSPB7, MAT1A, AK5, REG1A, C2orf49, CALML5, KMT2A, COCH | ANXA3, S100P, ERO1A, JUP, MUC1, MAL2, CDA, KRT19, AGR2, SFN, CAPS, NQO1, LCN2, LTF, PRTN3, CTSE, KRT8, S100A8, MPO, KRT75, AZU1, SERPINB5, S100A9, CAMP, VSIG1, KRT7, ANXA10, PRKCI, AAAS, KLHDC7B, DMKN, CLDN18, CAPNS2, OLFM4, KLC3, FGR, ABHD5, KHK, PSCA, LY6D, IFIH1 |
| Related pathways | Regulation of hormone levels, insulin regulation, regulation of TLR, PPAR signaling pathway | ECM, Collagen formation, MET related pathways, IGF regulation pathways, TGF-beta signaling | Metabolic related pathways | Neutrophil degranulation, keratinization, IRAK4 deficiency |
| Potential drug targets | ADH1B, F13A1, APOC3, PROCR, IGHG2, HBB, FGA, AOC3, HBA1, FLT1, PDE4D, CA1, FGG, TFR2, BCR, CACNA2D2, CD36, GPD1, SLC4A1, APCS, ANK2, ALOX15 | FN1, CPB2, AEBP1, HTRA1, LOX, MMP19, LOXL3, APCS, CDK12, SLC2A1 | ABAT, PNLIP, AMY2A, HDAC7, GAMT, DPEP1, CKB, ACAT1, MCEE, GATM, CEL, MCCC1, PSAT1, CRAT, PRSS1, CTRC, RNASEH2A, PHGDH, SARDH, CELA2A, GPT2 | ANXA3, CDA, PRKCI, PRTN3, MPO, AAAS, FGR, ABHD5, KHK, IFIH1 |

**Supplementary Table 4: Previous evidence regarding each of the proteins in the risk score**

| **Protein** | **Detected in Blood by MS*** | **Evidence in pancreatic cancer** | **Known Prognostic*** |
| --- | --- | --- | --- |
| **PURB** | Yes | No direct evidence in PDA, yet PURB was found to be associated with breast cancer, [^1^](#_ENREF_1)gastric cancer and leukemia[^2^](#_ENREF_2) | Yes (same direction) |
| **SDCBP2** | No | No direct evidence in PDA yet SDCBP2 has a role in AML, thyroid cancer and ovarian cancer [^3-5^](#_ENREF_3) | Yes (same direction) |
| **CD2BP2** | No | No direct evidence in PDA, yet CD2BP2 has a role in oesophageal SCC, and bladder cancer[^6^](#_ENREF_6)^,^[^7^](#_ENREF_7) | Yes (same direction) |
| **GALM** | Yes | NA | No |
| **SERPINA3** | Yes | SERPINA3 is of diagnostic and prognostic value in PDA[^8^](#_ENREF_8)^,^[^9^](#_ENREF_9) | Yes (same direction) |
| **OAS3** | Yes | OAS3 is of diagnostic value in PDA and is also associated with immune cell infiltration [^10^](#_ENREF_10) | Yes (same direction) |
| **FAN1** | No | FAN1 is known to be interacting with DNA repair proteins and may be associated with hereditary PDA[^11^](#_ENREF_11)^,^[^12^](#_ENREF_12) | Yes (same direction) |
| **ZPR1** | No | The association between ZPR1 and PDA is observed in several dataset [^13^](#_ENREF_13) | Yes (same direction) |
| **KRT2** | Yes | The relation of KRT2 with PDA is not very clear. However, a 9-gene based risk score that include KRT2 was prognostic in PDA[^14^](#_ENREF_14) | No |
| **NUDT2** | Yes | The role of NUDT2 in PDA is not clear. However, NUDT2 is associated with breast cancer poorer prognosis and response to immunotherapy [^15^](#_ENREF_15)^,^[^16^](#_ENREF_16) | Yes (same direction) |
| **SMNDC1** | No | The role of SMNDC1 in PDA is unknown. SMNDC1 may have a prognostic value in ovarian and lung cancer[^17^](#_ENREF_17)^,^[^18^](#_ENREF_18) | Yes (same direction) |
| **SERPINA4** | Yes | SERPINA4 is a cancer promoting gene, that seems to be overexpressed in PDA[^19^](#_ENREF_19)^,^[^20^](#_ENREF_20) | Yes (opposite direction) |
| **CUTA** | Yes | CUTA is overexpressed in some types of cancer including colorectal, liver and breast, however its role in PDA is unknown[^21^](#_ENREF_21) | Yes (same direction) |
| **WDR36** | No | NA | No |
| **POSTN** | Yes | POSTN is overexpressed in PDA and is associated with poorer prognosis[^22^](#_ENREF_22)^,^[^23^](#_ENREF_23) | Yes (same direction) |
| **CLEC11A** | Yes | CLEC11A is overexpressed in PDA and may be of diagnostic value [^24-26^](#_ENREF_24) | Yes (opposite direction) |
| **PEX14** | No | The role of PEX14 in PDA is not clear, however, PEX14 may be associated with breast cancer and of prognostic value in neuroblastoma [^27^](#_ENREF_27)^,^[^28^](#_ENREF_28) | No |
| **PI4KA** | No | PI4KA is upregulated in PDA and is associated with promoting progression and KRAS signaling [^29-31^](#_ENREF_29) | Yes (same direction) |

*Based on data extracted from the Human Protein Atlas (<https://www.proteinatlas.org/>)

**Supplementary Table 5: Clinical variables across the proteomic-based risk groups**

| Clinical Variables | | High Risk | Low Risk | p-value |
| --- | --- | --- | --- | --- |
|  |  | N = 94 | N = 21 |  |
| KRAS Status | KRAS Mutation Found | 76 (81%) | 12 (57%) | 0.02 |
|  | NO KRAS Mutation Found | 8 (9%) | 6 (29%) |  |
|  | Unknown | 10 (11%) | 3 (14%) |  |
| HRD status | Negative | 44 (47%) | 11 (52%) | 1 |
|  | Positive | 4 (4%) | 0 (0%) |  |
|  | Unknown | 46 (49%) | 10 (48%) |  |
| COSMIC Signature 2 | Negative | 42 (45%) | 11 (52%) | 0.58 |
|  | Positive | 6 (6%) | 0 (0%) |  |
|  | Unknown | 46 (49%) | 10 (48%) |  |
| COSMIC Signature 3 | Negative | 38 (40%) | 11 (52%) | 0.18 |
|  | Positive | 10 (11%) | 0 (0%) |  |
|  | Unknown | 46 (49%) | 10 (48%) |  |
| COSMIC Signature 5 | Negative | 33 (35%) | 4 (19%) | 0.08 |
|  | Positive | 15 (16%) | 7 (33%) |  |
|  | Unknown | 46 (49%) | 10 (48%) |  |
| COSMIC Signature 8 | Negative | 15 (16%) | 4 (19%) | 0.73 |
|  | Positive | 33 (35%) | 7 (33%) |  |
|  | Unknown | 46 (49%) | 10 (48%) |  |
| COSMIC Signature 9 | Negative | 44 (47%) | 10 (48%) | 1 |
|  | Positive | 4 (4%) | 1 (5%) |  |
|  | Unknown | 46 (49%) | 10 (48%) |  |
| COSMIC Signature 16 | Negative | 38 (40%) | 8 (38%) | 0.69 |
|  | Positive | 10 (11%) | 3 (14%) |  |
|  | Unknown | 46 (49%) | 10 (48%) |  |
| COSMIC Signature 17 | Negative | 42 (45%) | 9 (43%) | 0.63 |
|  | Positive | 6 (6%) | 2 (10%) |  |
|  | Unknown | 46 (49%) | 10 (48%) |  |
| KRAS Subtypes | No Mutation | 18 (19%) | 9 (43%) | 0.53 |
|  | Gln61Arg | 1 (1%) | 0 (0%) |  |
|  | Gln61His | 3 (3%) | 1 (5%) |  |
|  | Gly12Ala | 1 (1%) | 0 (0%) |  |
|  | Gly12Arg | 13 (14%) | 3 (14%) |  |
|  | Gly12Asp | 34 (36%) | 5 (24%) |  |
|  | Gly12Cys | 2 (2%) | 0 (0%) |  |
|  | Gly12Leu | 1 (1%) | 0 (0%) |  |
|  | Gly12Val | 21 (22%) | 3 (14%) |  |

Gln61Arg: Glutamine to Arginine at position 61, Gln61His: Glutamine to Histidine at position 61, Gly12Ala: Glycine to Alanine at position 12, Gly12Arg: Glycine to Arginine at position 12, Gly12Asp: Glycine to Aspartic Acid at position 12, Gly12Cys: Glycine to Cysteine at position 12, Gly12Leu: Glycine to Leucine at position 12, Gly12Val: Glycine to Valine at position 12.

Note that all patients showed positivity for COSMIC signature 1, while no patients showed positivity for COSMIC signatures 6, 20, 25, and 26. For COSMIC signatures 13, 18, 28, and 30, only one patient was in the positive group.

**Supplementary Table 6: Univariate and multivariate Cox regression model with Stepwise-AIC showing the association of each of the COSMIC signatures with overall survival.**

| **COSMIC Signatures** | **Univariate model** | | | | **Multivariate model with Step AIC** | | | |
| --- | --- | --- | --- | --- | --- | --- | --- | --- |
|  | **Hazard Ratio** | **Lower 95% CI** | **Upper 95% CI** | **P value** | **Hazard Ratio** | **Lower 95% CI** | **Upper 95% CI** | **P value** |
| **Sig2** | 1.31 | 0.56 | 3.09 | 0.532 | **3.47** | **1.23** | **9.77** | **0.019** |
| **Sig3** | **3.34** | **1.56** | **7.15** | **0.002** | **3.96** | **1.81** | **8.69** | **0.001** |
| **Sig5** | 0.71 | 0.41 | 1.25 | 0.241 | NA | NA | NA | NA |
| **Sig6** | NA | NA | NA | NA | NA | NA | NA | NA |
| **Sig8** | 1.29 | 0.71 | 2.36 | 0.401 | 1.65 | 0.83 | 3.29 | 0.152 |
| **Sig9** | 1.63 | 0.64 | 4.14 | 0.307 | NA | NA | NA | NA |
| **Sig13** | 0.43 | 0.06 | 3.18 | 0.411 | 0.21 | 0.02 | 1.85 | 0.158 |
| **Sig16** | 1.29 | 0.66 | 2.51 | 0.458 | NA | NA | NA | NA |
| **Sig17** | 0.67 | 0.28 | 1.58 | 0.362 | NA | NA | NA | NA |
| **Sig18** | 3.17 | 0.42 | 23.88 | 0.264 | 6.94 | 0.82 | 58.78 | 0.076 |
| **Sig20** | NA | NA | NA | NA | NA | NA | NA | NA |
| **Sig25** | NA | NA | NA | NA | NA | NA | NA | NA |
| **Sig26** | NA | NA | NA | NA | NA | NA | NA | NA |
| **Sig28** | 0.8 | 0.11 | 5.84 | 0.827 | NA | NA | NA | NA |
| **Sig30** | 0.8 | 0.11 | 5.84 | 0.827 | NA | NA | NA | NA |

Note that all patients showed positivity for COSMIC signature 1, while no patients showed positivity for COSMIC signatures 6, 20, 25, and 26. For COSMIC signatures 13, 18, 28, and 30, only one patient was in the positive group.

**Supplementary Table 7: List of DAP, pathways, and potential drug targets within COSMIC signatures of interest.**

| **COSMIC**  **Signature** | **List of top 10 up regulated** | **Top pathways** | **FDA/potential drug targets (among top 100 upregulated proteins)** |
| --- | --- | --- | --- |
| **Sig2 (n=6)** | IGHV3OR16-12, IFT20, CRLF3, IGLV3-9, CASP10, SPATA5, HLA-DQA1, ATF7IP, C19orf12, SAMD9L | Immunoglobulin production, RNA splicing pathways, epithelial cell differentiation and maintenance | AMACR, CASP10, COQ7, EPHB4, SPATA5 |
| **Sig3 (n=10)** | NAMPT, ITGAM, MNDA, GCA, FCGR3A, LCN2, SOD2, CSTA, LTF, NCF4 | Neutrophil degranulation, Extracellular matrix organization, immune response (defence response to bacterium, neutrophil extracellular trap formation, innate immune response, and complement system) | CDA, ELANE, FCGR3A, FN1, MMP9, SERPINE1, CASP14, GYS2, HTRA1, LOXL3, MPO, PDAI4, PLOD2, PRTN3, PXDN, SLC2A1, SOD2 |
| **Sig5 (n=23)** | RNASEH2A, CELA2A, CTRC, SLC17A5, ZG16, PRSS2, MRPL21, IGHV1-24, TOGARAM1, PNLIP | Protein digestion and pancreatic secretion | PNLIP, CELA2A, CLDN3, CTRC, PRSS1, RNASEH2A, SLC17A5 |
| **Sig8 (n= 42)** | IGHV1-69D, IGHV1-3, NEO1, MZT1, MUC5B, EDC3, TTC3, DSG1, SLC4A2, IGHV1-24 | Axonogenesis, adaptive immune response, and metabolic pathways (response to ketone, alcohol metabolism and lipid transport) | AKR1C2, F5, PNPLA8, SPAST |
| **Sig9 (n=5)** | DUOX2, LGALS8, RAB25, AIM2, CALML5, ZC3H14, TRAPPC9, PTRH1, RAB2B, CHKB | Response to virus, positive regulation of defense response, neutrophil degranulation, regulation of vesicle-mediated transport, and positive regulation of cell motility | CHKB, DUOX2, PRTN3 |
| **Sig16 (n=13)** | PHIP, WDR36, SLC9B2, SLC29A1, TMA16, WAS, LPGAT1, ZFAND1, THAP1, CD209 | Metabolic pathways (fatty acid and lipoprotein transport, and regulation of protein transport) | CDK12, LOXL3, OTUD6B |
| **Sig17 (n=8)** | IGHV3OR16-12, CNTRL, OTC, TIMMDC1, KDM4B, ANKRD27, EIF1AY, CDKN2C, ZC3H14, TGAL | Cell adhesion, and nuclear receptor meta-pathway | ITGAL, DUOX2, OTC, SLC20A2 |

Note that no patients showed positivity for COSMIC signatures 1,6,20,25,26, while COSMIC signatures 13, 18, 28, and 30 had only one patient in the positive group.

**Supplementary Table 8: Differential abundance of the S100 protein family across different clinical variables**

| **S100 Proteins** | **S100A10** | **S100A11** | **S100A12** | **S100A14** | **S100A16** | **S100A2** | **S100A4** | **S100A6** | **S100A7** | **S100A7L2** | **S100A8** | **S100A9** | **S100B** | **S100P** |
| --- | --- | --- | --- | --- | --- | --- | --- | --- | --- | --- | --- | --- | --- | --- |
| Tumor vs normal | up | up | ---- | ---- | ---- | ---- | up | up | ---- | ---- | ---- | ---- | Down | up |
| Dead vs alive | ---- | ---- | up | ---- | ---- | ---- | ---- | ---- | ---- | ---- | ---- | ---- | Down | up |
| Tumor location (Body/tail vs Head) | ---- | ---- | ---- | ---- | ---- | up | ---- | ---- | ---- | ---- | ---- | ---- | ---- | ---- |
| Recurrence Yes vs no | ---- | ---- | ---- | ---- | ---- | ---- | ---- | ---- | ---- | ---- | ---- | ---- | ---- | up |
| Grade high vs low | ---- | ---- | ---- | ---- | ---- | up | ---- | ---- | ---- | ---- | up | up | ---- | ---- |
| HRD yes vs no | ---- | ---- | up | ---- | ---- | up | ---- | ---- | ---- | ---- | up | up | ---- | ---- |
| Signature 3 Yes vs no | ---- | ---- | up | ---- | ---- | up | ---- | ---- | ---- | ---- | up | up | ---- | ---- |
| Signature 8 yes vs no | ---- | ---- | ---- | ---- | ---- | ---- | ---- | ---- | Down | ---- | ---- | ---- | ---- | ---- |
| Signature 9 yes vs no | ---- | ---- | ---- | up | ---- | ---- | ---- | ---- | ---- | ---- | ---- | ---- | ---- | ---- |
| Clusters 2,4 worst vs 1,3 best | ---- | ---- | ---- | ---- | ---- | ---- | ---- | ---- | ---- | ---- | up | ---- | Down | up |
| KRAS, Mut vs Wild | ---- | ---- | ---- | ---- | ---- | ---- | ---- | ---- | ---- | ---- | ---- | ---- | ---- | ---- |
| KRASG12A  Mut versus others G12 | ---- | ---- | ---- | up | up | ---- | ---- | ---- | ---- | ---- | ---- | ---- | ---- | ---- |
| KRASG12D  Mut versus others G12 | Down | ---- | ---- | ---- | ---- | ---- | ---- | ---- | ---- | ---- | ---- | ---- | ---- | ---- |
| KRASG12C  Mut versus others G12 | ---- | ---- | ---- | ---- | ---- | up | ---- | ---- | up | up | ---- | ---- | ---- | ---- |

*S100 A1, S100A13, and S100A3 were not differentially abundant among any subgroup

**Supplementary Table 9: Differential abundance of the KRT protein family across different clinical variables**

| **KRT protein*** | **KRT1** | **KRT2** | **KRT4** | **KRT5** | **KRT**  **6A** | **KRT6B** | **KRT7** | **KRT8** | **KRT**  **13** | **KRT**  **14** | **KRT**  **15** | **KRT**  **16** | **KRT**  **17** | **KRT**  **18** | **KRT**  **19** | **KRT**  **24** | **KRT**  **35** | **KRT**  **72** | **KRT**  **73** | **KRT**  **75** | **KRT**  **85** |
| --- | --- | --- | --- | --- | --- | --- | --- | --- | --- | --- | --- | --- | --- | --- | --- | --- | --- | --- | --- | --- | --- |
| Tumor vs normal | ---- | ---- | ---- | ---- | up | ---- | up | ---- | ---- | ---- | ---- | Down | up | ---- | up | ---- | Down | up | ---- | ---- | up |
| Dead vs alive | ---- | ---- | ---- | ---- |  | Down | up | up | ---- | ---- | ---- | ---- |  | up | up | ---- | ---- | ---- | ---- | ---- | up |
| Tumor location (Body/tail vs Head) | ---- | ---- | ---- | up | up | ---- | ---- | ---- | up | ---- | ---- | ---- | ---- | ---- | ---- | ---- | ---- | ---- | ---- | Down | ---- |
| Recurrence Y vs N | ---- | ---- | ---- | ---- | ---- | ---- | up | up | ---- | ---- | ---- | ---- | ---- | up | up | ---- | ---- | ---- | ---- | ---- | ---- |
| Grade High vs low | ---- | ---- | ---- |  | up | ---- | ---- | ---- | up | ---- | ---- | up | up | ---- | ---- | Down | Down | up | ---- | Down | ---- |
| HRD Y vs N | ---- | ---- | ---- | up | up | up | ---- | ---- | ---- | ---- | ---- | up | ---- | ---- | ---- | ---- | ---- | ---- | up | ---- | ---- |
| Sig 3 | ---- | ---- | ---- | up | up | up | ---- | ---- | up | ---- | up | up | up | ---- | ---- | ---- | ---- | ---- | ---- | ---- | ---- |
| Sig 2 | ---- | ---- | ---- | ---- | ---- | ---- | ---- | ---- | ---- | ---- | ---- | ---- | ---- | ---- | ---- | ---- | up | ---- | ---- | ---- | ---- |
| Sig5 | ---- | ---- | ---- | ---- | ---- | ---- | ---- | ---- | Down | ---- | ---- | ---- | ---- | ---- | ---- | ---- | Down | ---- | ---- | ---- | ---- |
| Sig9 | ---- | ---- | Down | ---- | ---- | ---- | ---- | ---- | ---- | ---- | ---- | ---- | ---- | ---- | ---- | ---- | ---- | ---- | ---- | ---- | ---- |
| Sig16 | ---- | ---- | ---- | ---- | ---- | ---- | ---- | Down | ---- | ---- | ---- | ---- | ---- | ---- | ---- | ---- | ---- | ---- | ---- | ---- | ---- |
| Sig17 | ---- | ---- | ---- | ---- | ---- | ---- | ---- | ---- | ---- | ---- | ---- | ---- | ---- | ---- | ---- | ---- | ---- | up | ---- | ---- | up |
| KRAS | ---- | ---- | ---- | ---- | ---- | ---- | ---- | ---- | ---- | ---- | ---- | ---- | ---- | ---- | up | ---- | ---- |  | ---- | ---- | ---- |
| KRASG12R | ---- | ---- | ---- | Down | ---- | ---- | ---- | ---- | ---- | ---- | ---- | Down | ---- | ---- | ---- | ---- | ---- | ---- | ---- | ---- | ---- |
| KRASG12C | Down | Down | ---- | up | up | ---- | Down | ---- | ---- | up | up | up | ---- | ---- | ---- | ---- | ---- | ---- | Down | ---- | Down |

*KRT9, KRT10, KRT20, KRT23, KRT77, KRT 74, KRT79 and KRT80 were not differentially abundant among any subgroup

# Supplementary Figures

**Supplementary Figure 1:** Shows the main pathways associated with lists of proteins of interest. **(A)** Summary of Enrichment analysis in DisGeNET for the 20-protein panel, showing a highly significant association with pancreatic neoplasm. **(B)** Summary of the main pathways associated with proteins uniquely identified when restricting the differential abundance analysis to samples with ≥ 50% cancer content. **(C)** Summary of the main pathways associated with proteins uniquely identified to be related to pancreatic tumor cells after subtracting the list of proteins associated with fibroblasts and collagen deposition. All the figure was generated from the Metascape website:

(<https://metascape.org/gp/index.html#/main/step1> ).

**Supplementary Figure 2:** Consensus clustering plots. **(A)** This delta area plot displays the relative change in the cumulative distribution function (CDF) curve comparing k and k-1 clusters from our cohort. **(B)** The silhouette plot displays the silhouette width for the four clusters determined by consensus clustering within this cohort. **(C)** Kaplan-Meier plot of the recurrence-free survival rates of the combined clusters (Clusters 1 with 3 and Clusters 2 with 4).

**Supplementary Figure 3**: Differential abundance and Pathway enrichment analyses of clusters. **(A)** Volcano plot displaying the differentially expressed proteins between PDA samples classified between groups 1 and 3 against groups 2 and 4. **(B)** Pathways enriched in KEGG, Reactome, and Wikipathways for proteins upregulated in samples from clusters 2 and 4 compared to clusters 1 and 3.

**Supplementary Figure 4**: Kaplan-Meier survival curves for each of the 18 proteins within the risk score. Median cut-off was used to dichotomize patients into two groups.

**Supplementary Figure 5:** Proteomic information for the 18 risk-score proteins. **(A)** Peptide detection rate within the dataset. **(B)** Protein Missingness in our tumor data only. **(C)** Protein Missingness in CPTAC data.

**Supplementary Figure 6:** A forest plot detailing hazard ratios of the proteomic risk score and clinically relevant variables for PDA within our cohort using multivariable Cox regression modeling. *Margin R+: Positive margin, R0: Negative margin.*

**Supplementary Figure 7**: Proteomic signature performance. **(A)** Receiver operator characteristic curve (ROC) at 1 year of follow-up for the proteomic signature (purple) and other clinically relevant variables for PDA within our cohort. **(B)** This plot displays the AUC as a function of time for the proteomic risk score (purple) and other clinically relevant variables for PDA within our cohort.

**Supplementary Figure 8: (A)** Kaplan-Meier curve displaying three-year survival for groups dichotomized by proteomic risk score in ProCan data. **(B)** Kaplan-Meier curve displaying recurrence-free survival for groups dichotomized by proteomic risk score in ProCan data.

**Supplementary Figure 9:** Kaplan-Meier plot for patients dichotomized by a proteomic risk score that uses only the ten proteins detected in blood by mass spectrometry (PURB, GALM, SERPINA3, OAS3, KRT2, NUDT2, SERPINA4, CUTA, POSTN, CLEC11A).

**Supplementary Figure 10**: Differential abundance and Pathway enrichment analyses of KRAS mutations. **(A)** Volcano plot displaying the differentially expressed proteins between KRAS mutant PDA and KRAS wild-type PDA. **(B)** Pathways enriched in Gene Ontology molecular function database from upregulated proteins in tumors that harbored KRAS mutations.

**Supplementary Figure 11:** Differential abundance and Pathway enrichment analyses of KRAS-G12C. **(A)** Volcano plot displaying the differentially expressed proteins between tumors harboring KRAS-G12C mutation and those with any other KRAS-G12 mutation. **(B)** Pathways enriched in the KEGG, Reactome, and WikiPathways databases from upregulated proteins from tumors that harbored KRAS-G12C mutations when compared to other tumors with other KRAS12 mutations.

**Supplementary Figure 12:** Volcano plot displaying the differentially expressed proteins between tumors harboring a KRAS-G12D mutation and those with any other KRAS-G12 mutation.

**Supplementary Figure 13:** Differential abundance and Pathway enrichment analyses of HRD. **(A)** Volcano plot displaying the differentially expressed proteins between tumor samples with and without HRD. **(B)** Pathways enriched in KEGG, Reactome, and Wikipathways databases from upregulated proteins in tumors from patients with HRD-positive status.

**Supplementary Figure 14**: Differential abundance and Pathway enrichment analyses of COSMIC Signature 3. **(A)** Volcano plot displaying the differentially expressed proteins between tumors with and without somatic mutations consistent with COSMIC Signature 3. **(B)** Pathways enriched in Reactome from upregulated proteins in tumors that displayed mutations corresponding to COSMIC Signature 3.

**Supplementary Figure 15:** Forest plot showing the hazard ratios of each of the S100 proteins detected within the tumor samples of this cohort, using a univariate Cox regression modeling with overall survival as the outcome of interest.

**Supplementary Figure 16:** Forest plot showing the hazard ratios of each of the KRT proteins detected within the tumor samples of this cohort using a univariate Cox regression modeling with overall survival as the outcome of interest. Note that this figure excludes KRT75, which was detected in only two samples.

**Additional References**

1. Chang K-C, Diermeier SD, Yu AT, et al: MaTAR25 lncRNA regulates the Tensin1 gene to impact breast cancer progression. Nature Communications 11:6438, 2020

2. Shi J, Cheng C, Ma J, et al: Gene expression signature for detection of gastric cancer in peripheral blood. Oncology Letters 15:9802-9810, 2018

3. Rao Y, Liu H, Yan X, et al: In silico analysis identifies differently expressed lncRNAs as novel biomarkers for the prognosis of thyroid cancer. Computational and Mathematical Methods in Medicine 2020, 2020

4. Du Y, Li L-L, Chen F: Targeting SDCBP2 in acute myeloid leukemia. Cellular Signalling 112:110889, 2023

5. Liu X, Liu C, Zhang A, et al: Long non-coding RNA SDCBP2-AS1 delays the progression of ovarian cancer via microRNA-100-5p-targeted EPDR1. World Journal of Surgical Oncology 19:1-9, 2021

6. Guo X, Li G, Zhao Y, et al: TGFB Induced Factor Homeobox 2 Induces Deterioration of Bladder Carcinoma via Activating CD2 Cytoplasmic Tail Binding Protein 2. Journal of Biomedical Nanotechnology 19:1670-1676, 2023

7. Li Y, Yang B, Ma Y, et al: Phosphoproteomics reveals therapeutic targets of esophageal squamous cell carcinoma. Signal Transduction and Targeted Therapy 6:381, 2021

8. Soman A, Nair SA: Unfolding the cascade of SERPINA3: Inflammation to cancer. Biochimica et Biophysica Acta (BBA)-Reviews on Cancer:188760, 2022

9. Mawaribuchi S, Shimomura O, Oda T, et al: rBC2LCN-reactive SERPINA3 is a glycobiomarker candidate for pancreatic ductal adenocarcinoma. Glycobiology 33:342-352, 2023

10. Gao L-J, Li J-L, Yang R-R, et al: Biological characterization and clinical value of OAS gene family in pancreatic cancer. Frontiers in Oncology 12:884334, 2022

11. Deshmukh AL, Porro A, Mohiuddin M, et al: FAN1, a DNA repair nuclease, as a modifier of repeat expansion disorders. Journal of Huntington's Disease 10:95-122, 2021

12. Smith AL, Alirezaie N, Connor A, et al: Candidate DNA repair susceptibility genes identified by exome sequencing in high-risk pancreatic cancer. Cancer letters 370:302-312, 2016

13. He L, Xie Y, Qiu Y, et al: Pan-Cancer Profiling and Digital Pathology Analysis Reveal Negative Prognostic Biomarker ZPR1 Associated with Immune Infiltration and Treatment Response in Hepatocellular Carcinoma. Journal of Hepatocellular Carcinoma:1309-1325, 2023

14. Yu X, Wang Y, Shi X, et al: Dysfunctional epigenetic protein-coding gene-related signature is associated with the prognosis of pancreatic cancer based on histone modification and transcriptome analysis. Scientific Reports 13:146, 2023

15. Marriott AS, Vasieva O, Fang Y, et al: NUDT2 disruption elevates diadenosine tetraphosphate (Ap4A) and down-regulates immune response and cancer promotion genes. PLoS One 11:e0154674, 2016

16. Wright RH, Beato M: Role of the NUDT enzymes in breast cancer. International Journal of Molecular Sciences 22:2267, 2021

17. Ye Y, Li L, Dai Q, et al: Comprehensive analysis of histone methylation modification regulators for predicting prognosis and drug sensitivity in lung adenocarcinoma. Frontiers in Cell and Developmental Biology 10:991980, 2022

18. Giri K, Shameer K, Zimmermann MT, et al: Understanding protein–nanoparticle interaction: a new gateway to disease therapeutics. Bioconjugate chemistry 25:1078-1090, 2014

19. Marin AM, Batista M, Korte de Azevedo AL, et al: Screening of Exosome-Derived Proteins and Their Potential as Biomarkers in Diagnostic and Prognostic for Pancreatic Cancer. International Journal of Molecular Sciences 24:12604, 2023

20. Zhu P, Ge N, Liu D, et al: Preliminary investigation of the function of hsa_circ_0006215 in pancreatic cancer. Oncology Letters 16:603-611, 2018

21. Blockhuys S, Celauro E, Hildesjö C, et al: Defining the human copper proteome and analysis of its expression variation in cancers. Metallomics 9:112-123, 2017

22. Neuzillet C, Tijeras-Raballand A, Ragulan C, et al: Inter- and intra-tumoural heterogeneity in cancer-associated fibroblasts of human pancreatic ductal adenocarcinoma. J Pathol 248:51-65, 2019

23. Dong D, Jia L, Zhang L, et al: Periostin and CA242 as potential diagnostic serum biomarkers complementing CA19.9 in detecting pancreatic cancer. Cancer Science 109:2841-2851, 2018

24. Natale F, Vivo M, Falco G, et al: Deciphering DNA methylation signatures of pancreatic cancer and pancreatitis. Clinical Epigenetics 11:1-12, 2019

25. Hasan S, Jacob R, Manne U, et al: Advances in pancreatic cancer biomarkers. Oncology reviews 13, 2019

26. Kisiel JB, Raimondo M, Taylor WR, et al: New DNA Methylation Markers for Pancreatic Cancer: Discovery, Tissue Validation, and Pilot Testing in Pancreatic Juice. Clinical Cancer Research 21:4473-4481, 2015

27. Fransson S, Martinsson T, Ejeskär K: Neuroblastoma tumors with favorable and unfavorable outcomes: Significant differences in mRNA expression of genes mapped at 1p36.2. Genes, Chromosomes and Cancer 46:45-52, 2007

28. Bodelon C, Oh H, Chatterjee N, et al: Association between breast cancer genetic susceptibility variants and terminal duct lobular unit involution of the breast. International Journal of Cancer 140:825-832, 2017

29. Kattan WE, Liu J, Montufar-Solis D, et al: Components of the phosphatidylserine endoplasmic reticulum to plasma membrane transport mechanism as targets for KRAS inhibition in pancreatic cancer. Proceedings of the National Academy of Sciences 118:e2114126118, 2021

30. Zhang Y, Ji S, Zhang X, et al: Human CPTP promotes growth and metastasis via sphingolipid metabolite ceramide and PI4KA/AKT signaling in pancreatic cancer cells. Int J Biol Sci 18:4963-4983, 2022

31. Adhikari H, Kattan WE, Kumar S, et al: Oncogenic KRAS is dependent upon an EFR3A-PI4KA signaling axis for potent tumorigenic activity. Nature Communications 12:5248, 2021
