# Supplementary material for: Mapping the Proteomic Landscape of Pancreatic Cancer: Prognostic Insights and Subtype Stratification
Source: Cancer Res Commun. 2025 Oct 23;5(10):1879–93. doi: 10.1158/2767-9764.CRC-25-0229 (PMC12548992; doi:10.1158/2767-9764.CRC-25-0229)
Supplement: Supplementary Table 1 — shows additional clinical characteristics of the study cohort. Gln61Arg: Glutamine to Arginine at position 61, Gln61His: Glutamine to Histidine at position 61, Gly12Ala: Glycine to Alanine at position 12, Gly12Arg: Glycine to Arginine at position 12, Gly12Asp: Glycine to Aspartic Acid at position 12, Gly12Cys: Glycine to Cysteine at position 12, Gly12Leu: Glycine to Leucine at position 12, Gly12Val: Glycine to Valine at position 12 [file crc-25-0229_supplementary_table_1_suppst1.docx]

**Supplementary Table 1: Additional clinical characteristics of the study cohort**

| Clinical variables | | Alive | Dead | Total | p-value |
| --- | --- | --- | --- | --- | --- |
|  |  | N = 16 | N = 99 | N = 115 |  |
| Perineural invasion (PNI) | Absent | 6 (38%) | 15 (15%) | 21 (18%) | 0.13 |
|  | Present | 10 (62%) | 80 (81%) | 90 (78%) |  |
|  | Unknown | 0 (0%) | 4 (4%) | 4 (4%) |  |
| Lymphovascular invasion (LVI) | Absent | 11 (69%) | 34 (34%) | 45 (39%) | **0.04** |
|  | Present | 5 (31%) | 61 (62%) | 66 (57%) |  |
|  | Unknown | 0 (0%) | 4 (4%) | 1 (4%) |  |
| COSMIC Signature 2 | Negative | 8 (50%) | 45 (45%) | 53 (46%) | 0.58 |
|  | Positive | 0 (0%) | 6 (6%) | 6 (5%) |  |
|  | Unknown | 8 (50%) | 48 (48%) | 56 (49%) |  |
| COSMIC Signature 3 | Negative | 7 (44%) | 42 (42%) | 49 (43%) | 1 |
|  | Positive | 1 (6%) | 9 (9%) | 10 (9%) |  |
|  | Unknown | 8 (50%) | 48 (48%) | 56 (49%) |  |
| COSMIC Signature 5 | Negative | 4 (25%) | 33 (33%) | 37 (32%) | 0.46 |
|  | Positive | 4 (25%) | 18 (18%) | 22 (19%) |  |
|  | Unknown | 8 (50%) | 48 (48%) | 56 (49%) |  |
| COSMIC Signature 8 | Negative | 4 (25%) | 15 (15%) | 19 (17%) | 0.42 |
|  | Positive | 4 (25%) | 36 (36%) | 40 (35%) |  |
|  | Unknown | 8 (50%) | 48 (48%) | 56 (49%) |  |
| COSMIC Signature 9 | Negative | 8 (50%) | 46 (46%) | 54 (47%) | 1 |
|  | Positive | 0 (0%) | 5 (5%) | 5 (4%) |  |
|  | Unknown | 8 (50%) | 48 (48%) | 56 (49%) |  |
| COSMIC Signature 16 | Negative | 6 (38%) | 40 (40%) | 46 (40%) | 1 |
|  | Positive | 2 (12%) | 11 (11%) | 13 (11%) |  |
|  | Unknown | 8 (50%) | 48 (48%) | 56 (49%) |  |
| COSMIC Signature 17 | Negative | 6 (38%) | 45 (45%) | 51 (44%) | 0.3 |
|  | Positive | 2 (12%) | 6 (6%) | 8 (7%) |  |
|  | Unknown | 8 (50%) | 48 (48%) | 56 (49%) |  |
| KRAS Subtypes | No Mutation | 7 (44%) | 20 (20%) | 27 (23%) | **0.03** |
|  | Gln61Arg | 1 (6%) | 0 (0%) | 1 (<1%) |  |
|  | Gln61His | 1 (6%) | 3 (3%) | 4 (3%) |  |
|  | Gly12Ala | 0 (0%) | 1 (1%) | 1 (<1%) |  |
|  | Gly12Arg | 0 (0%) | 16 (16%) | 16 (14%) |  |
|  | Gly12Asp | 3 (19%) | 36 (36%) | 39 (34%) |  |
|  | Gly12Cys | 1 (6%) | 1 (1%) | 2 (2%) |  |
|  | Gly12Leu | 0 (0%) | 1 (1%) | 1 (<1%) |  |
|  | Gly12Val | 3 (19%) | 21 (21%) | 24 (21%) |  |

Supplementary Table 1 shows additional clinical characteristics of the study cohort.
Gln61Arg: Glutamine to Arginine at position 61, Gln61His: Glutamine to Histidine at position 61, Gly12Ala: Glycine to Alanine at position 12, Gly12Arg: Glycine to Arginine at position 12, Gly12Asp: Glycine to Aspartic Acid at position 12, Gly12Cys: Glycine to Cysteine at position 12, Gly12Leu: Glycine to Leucine at position 12, Gly12Val: Glycine to Valine at position 12
