# Supplementary material for: Mapping the Proteomic Landscape of Pancreatic Cancer: Prognostic Insights and Subtype Stratification
Source: Cancer Res Commun. 2025 Oct 23;5(10):1879–93. doi: 10.1158/2767-9764.CRC-25-0229 (PMC12548992; doi:10.1158/2767-9764.CRC-25-0229)
Supplement: Supplementary Table 3 — shows the differentially abundant proteins, their associated pathways, and the potential drug targets identified across the four proteomic-based clusters. [file crc-25-0229_supplementary_table_3_suppst3.docx]

**Supplementary Table 3: Differentially abundant proteins, pathways and potential therapeutic targets within each cluster.**

|  | Cluster 1 | Cluster 2 | Cluster 3 | Cluster 4 |
| --- | --- | --- | --- | --- |
| Upregulated differentially abundant proteins | ADH1B, FABP4, GNG2, S100B, F13A1, APOC3, SPON1, HSPA12A, APOB, PROCR, CRYAB, TMEM119, IGHG2, HBB, APOC2, FGA, HSPB6, AOC3, HBA1, FLT1, GZMK, COL14A1, OLFML1, LAMB2, HBD, PDE4D, CPA3, CA1, FGG, MAN1C1, OGN, FHL1, CD36, GPD1, SLC4A1, PAM, CMA1, IGHV2-70D, CPE, APCS, ZFPM2, TMEM41B, ELN, LPA, SOD3, TFR2, BCR, SCG5, SCGN, MTUS1, PCSK1N, THAP1, CACNA2D2, PTPRN, CADPS, DMRT2, C1RL, PLIN1, CDHR5, CHGA, CD38, TAGLN3, GCG, AFP, ANAPC7, ANK2, GUF1, ALOX15, DES, TMEM40 | SRPX2, AEBP1, VPS11, ANGPTL2, COL12A1, MFGE8, TGFBI, POSTN, THBS1, FN1, THBS2, FBLN2, FMOD, SERPINF1, EFEMP2, MXRA5, COL11A1, ECM1, EDIL3, COL8A1, CPB2, CLEC11A, MATN1, COMP, HTRA1, EFEMP1, LOX, BGN, LTBP1, FBLN1, CCDC80, HTRA3, ISLR, BRCA2, VTN, GREM1, COL24A1, C1QTNF5, C8G, OAF, CTHRC1, CDH13, MMP28, PRELP, MMP19, ASPN, LOXL3, GPX3, VCAN, C4orf54, CILP, APCS, FBN1, TNC, MAMDC2, INHBA, PLIN2, CDK12, MFAP4, LAMB3, COL11A2, LECT2, OLFML2B, SLC2A1, HBG1, CRP, KRT6B, HAX1, MRPS2, AGFG2, ANGPTL4, CEACAM6, LOXL2, PLXNB1, A2ML1 | ACAT1, PDCD4, ABAT, MCEE, GATM, PNLIP, PDIA2, RRBP1, CEL, CPA1, SYCN, NUCB2, PRSS2, CELA3A, MCCC1, IMPA2, FBXO44, SCLY, ECI2, PNLIPRP2, LRRC59, PSAT1, CPB1, CRAT, FAHD2A, ACSS1, CHDH, PRSS1, ALDH1L2, SPINK1, EIF4EBP1, ELAPOR1, ERP27, PBLD, CTRC, TOGARAM1, RNASEH2A, GP2, PIK3C3, AMY2A, FKBP11, RGN, PHGDH, SARDH, DAPK1, EPHX2, AMY2B, GSTA2, NOD1, HDAC7, LGALS2, CELA2A, CLPS, KLK1, ZFP69B, PLA2G4D, ASRGL1, CELA3B, GAMT, MYL4, KIF21B, CIT, EIF2D, TRAM1, SEC11C, GRB10, FAM174B, NME2P1, RGS22, CHGB, DPEP1, NRCAM, CTRL, CKB, TCEA3, CELA2B, HOGA1, HOOK1, TPST2, PKIG, CPE, PAIP2B, TMSB10, TMEM97, SERPINI2, PRR14L, CHGA, ACADL, LRATD2, MYO5B, ARSL, CPA5, UGT2B4, HMGCS2, GIP, PNLIPRP1, TIMM23, RTL1, GPT2, XIRP2, SLC43A1, PCF11, CKMT2, ZG16, KRT35, L1RE1, KPNA7, IGF2BP1, HSPB7, MAT1A, AK5, REG1A, C2orf49, CALML5, KMT2A, COCH | ANXA3, S100P, ERO1A, JUP, MUC1, MAL2, CDA, KRT19, AGR2, SFN, CAPS, NQO1, LCN2, LTF, PRTN3, CTSE, KRT8, S100A8, MPO, KRT75, AZU1, SERPINB5, S100A9, CAMP, VSIG1, KRT7, ANXA10, PRKCI, AAAS, KLHDC7B, DMKN, CLDN18, CAPNS2, OLFM4, KLC3, FGR, ABHD5, KHK, PSCA, LY6D, IFIH1 |
| Related pathways | Regulation of hormone levels, insulin regulation, regulation of TLR, PPAR signaling pathway | ECM, Collagen formation, MET related pathways, IGF regulation pathways, TGF-beta signaling | Metabolic related pathways | Neutrophil degranulation, keratinization, IRAK4 deficiency |
| Potential drug targets | ADH1B, F13A1, APOC3, PROCR, IGHG2, HBB, FGA, AOC3, HBA1, FLT1, PDE4D, CA1, FGG, TFR2, BCR, CACNA2D2, CD36, GPD1, SLC4A1, APCS, ANK2, ALOX15 | FN1, CPB2, AEBP1, HTRA1, LOX, MMP19, LOXL3, APCS, CDK12, SLC2A1 | ABAT, PNLIP, AMY2A, HDAC7, GAMT, DPEP1, CKB, ACAT1, MCEE, GATM, CEL, MCCC1, PSAT1, CRAT, PRSS1, CTRC, RNASEH2A, PHGDH, SARDH, CELA2A, GPT2 | ANXA3, CDA, PRKCI, PRTN3, MPO, AAAS, FGR, ABHD5, KHK, IFIH1 |

Supplementary Table 3 shows the differentially abundant proteins, their associated pathways, and the potential drug targets identified across the four proteomic-based clusters.
