# Supplementary material for: Mapping the Proteomic Landscape of Pancreatic Cancer: Prognostic Insights and Subtype Stratification
Source: Cancer Res Commun. 2025 Oct 23;5(10):1879–93. doi: 10.1158/2767-9764.CRC-25-0229 (PMC12548992; doi:10.1158/2767-9764.CRC-25-0229)
Supplement: Supplementary Table 4 — shows previous evidence regarding each protein included in the risk score in terms of their association with different types of cancer diagnosis and prognosis. *Based on data extracted from the Human Protein Atlas (https://www.proteinatlas.org/) [file crc-25-0229_supplementary_table_4_suppst4.docx]

**Supplementary Table 4: Previous evidence regarding each of the proteins in the risk score**

| **Protein** | **Detected in Blood by MS*** | **Evidence in pancreatic cancer** | **Known Prognostic*** |
| --- | --- | --- | --- |
| **PURB** | Yes | No direct evidence in PDA, yet PURB was found to be associated with breast cancer, [^1^](#_ENREF_1)gastric cancer and leukemia[^2^](#_ENREF_2) | Yes (same direction) |
| **SDCBP2** | No | No direct evidence in PDA yet SDCBP2 has a role in AML, thyroid cancer and ovarian cancer [^3-5^](#_ENREF_3) | Yes (same direction) |
| **CD2BP2** | No | No direct evidence in PDA, yet CD2BP2 has a role in oesophageal SCC, and bladder cancer[^6^](#_ENREF_6)^,^[^7^](#_ENREF_7) | Yes (same direction) |
| **GALM** | Yes | NA | No |
| **SERPINA3** | Yes | SERPINA3 is of diagnostic and prognostic value in PDA[^8^](#_ENREF_8)^,^[^9^](#_ENREF_9) | Yes (same direction) |
| **OAS3** | Yes | OAS3 is of diagnostic value in PDA and is also associated with immune cell infiltration [^10^](#_ENREF_10) | Yes (same direction) |
| **FAN1** | No | FAN1 is known to be interacting with DNA repair proteins and may be associated with hereditary PDA[^11^](#_ENREF_11)^,^[^12^](#_ENREF_12) | Yes (same direction) |
| **ZPR1** | No | The association between ZPR1 and PDA is observed in several dataset [^13^](#_ENREF_13) | Yes (same direction) |
| **KRT2** | Yes | The relation of KRT2 with PDA is not very clear. However, a 9-gene based risk score that include KRT2 was prognostic in PDA[^14^](#_ENREF_14) | No |
| **NUDT2** | Yes | The role of NUDT2 in PDA is not clear. However, NUDT2 is associated with breast cancer poorer prognosis and response to immunotherapy [^15^](#_ENREF_15)^,^[^16^](#_ENREF_16) | Yes (same direction) |
| **SMNDC1** | No | The role of SMNDC1 in PDA is unknown. SMNDC1 may have a prognostic value in ovarian and lung cancer[^17^](#_ENREF_17)^,^[^18^](#_ENREF_18) | Yes (same direction) |
| **SERPINA4** | Yes | SERPINA4 is a cancer promoting gene, that seems to be overexpressed in PDA[^19^](#_ENREF_19)^,^[^20^](#_ENREF_20) | Yes (opposite direction) |
| **CUTA** | Yes | CUTA is overexpressed in some types of cancer including colorectal, liver and breast, however its role in PDA is unknown[^21^](#_ENREF_21) | Yes (same direction) |
| **WDR36** | No | NA | No |
| **POSTN** | Yes | POSTN is overexpressed in PDA and is associated with poorer prognosis[^22^](#_ENREF_22)^,^[^23^](#_ENREF_23) | Yes (same direction) |
| **CLEC11A** | Yes | CLEC11A is overexpressed in PDA and may be of diagnostic value [^24-26^](#_ENREF_24) | Yes (opposite direction) |
| **PEX14** | No | The role of PEX14 in PDA is not clear, however, PEX14 may be associated with breast cancer and of prognostic value in neuroblastoma [^27^](#_ENREF_27)^,^[^28^](#_ENREF_28) | No |
| **PI4KA** | No | PI4KA is upregulated in PDA and is associated with promoting progression and KRAS signaling [^29-31^](#_ENREF_29) | Yes (same direction) |

Supplementary Table 4 shows previous evidence regarding each protein included in the risk score in terms of their association with different types of cancer diagnosis and prognosis. *Based on data extracted from the Human Protein Atlas (<https://www.proteinatlas.org/>)

**Related References**

1. Chang K-C, Diermeier SD, Yu AT, et al: MaTAR25 lncRNA regulates the Tensin1 gene to impact breast cancer progression. Nature Communications 11:6438, 2020

2. Shi J, Cheng C, Ma J, et al: Gene expression signature for detection of gastric cancer in peripheral blood. Oncology Letters 15:9802-9810, 2018

3. Rao Y, Liu H, Yan X, et al: In silico analysis identifies differently expressed lncRNAs as novel biomarkers for the prognosis of thyroid cancer. Computational and Mathematical Methods in Medicine 2020, 2020

4. Du Y, Li L-L, Chen F: Targeting SDCBP2 in acute myeloid leukemia. Cellular Signalling 112:110889, 2023

5. Liu X, Liu C, Zhang A, et al: Long non-coding RNA SDCBP2-AS1 delays the progression of ovarian cancer via microRNA-100-5p-targeted EPDR1. World Journal of Surgical Oncology 19:1-9, 2021

6. Guo X, Li G, Zhao Y, et al: TGFB Induced Factor Homeobox 2 Induces Deterioration of Bladder Carcinoma via Activating CD2 Cytoplasmic Tail Binding Protein 2. Journal of Biomedical Nanotechnology 19:1670-1676, 2023

7. Li Y, Yang B, Ma Y, et al: Phosphoproteomics reveals therapeutic targets of esophageal squamous cell carcinoma. Signal Transduction and Targeted Therapy 6:381, 2021

8. Soman A, Nair SA: Unfolding the cascade of SERPINA3: Inflammation to cancer. Biochimica et Biophysica Acta (BBA)-Reviews on Cancer:188760, 2022

9. Mawaribuchi S, Shimomura O, Oda T, et al: rBC2LCN-reactive SERPINA3 is a glycobiomarker candidate for pancreatic ductal adenocarcinoma. Glycobiology 33:342-352, 2023

10. Gao L-J, Li J-L, Yang R-R, et al: Biological characterization and clinical value of OAS gene family in pancreatic cancer. Frontiers in Oncology 12:884334, 2022

11. Deshmukh AL, Porro A, Mohiuddin M, et al: FAN1, a DNA repair nuclease, as a modifier of repeat expansion disorders. Journal of Huntington's Disease 10:95-122, 2021

12. Smith AL, Alirezaie N, Connor A, et al: Candidate DNA repair susceptibility genes identified by exome sequencing in high-risk pancreatic cancer. Cancer letters 370:302-312, 2016

13. He L, Xie Y, Qiu Y, et al: Pan-Cancer Profiling and Digital Pathology Analysis Reveal Negative Prognostic Biomarker ZPR1 Associated with Immune Infiltration and Treatment Response in Hepatocellular Carcinoma. Journal of Hepatocellular Carcinoma:1309-1325, 2023

14. Yu X, Wang Y, Shi X, et al: Dysfunctional epigenetic protein-coding gene-related signature is associated with the prognosis of pancreatic cancer based on histone modification and transcriptome analysis. Scientific Reports 13:146, 2023

15. Marriott AS, Vasieva O, Fang Y, et al: NUDT2 disruption elevates diadenosine tetraphosphate (Ap4A) and down-regulates immune response and cancer promotion genes. PLoS One 11:e0154674, 2016

16. Wright RH, Beato M: Role of the NUDT enzymes in breast cancer. International Journal of Molecular Sciences 22:2267, 2021

17. Ye Y, Li L, Dai Q, et al: Comprehensive analysis of histone methylation modification regulators for predicting prognosis and drug sensitivity in lung adenocarcinoma. Frontiers in Cell and Developmental Biology 10:991980, 2022

18. Giri K, Shameer K, Zimmermann MT, et al: Understanding protein–nanoparticle interaction: a new gateway to disease therapeutics. Bioconjugate chemistry 25:1078-1090, 2014

19. Marin AM, Batista M, Korte de Azevedo AL, et al: Screening of Exosome-Derived Proteins and Their Potential as Biomarkers in Diagnostic and Prognostic for Pancreatic Cancer. International Journal of Molecular Sciences 24:12604, 2023

20. Zhu P, Ge N, Liu D, et al: Preliminary investigation of the function of hsa_circ_0006215 in pancreatic cancer. Oncology Letters 16:603-611, 2018

21. Blockhuys S, Celauro E, Hildesjö C, et al: Defining the human copper proteome and analysis of its expression variation in cancers. Metallomics 9:112-123, 2017

22. Neuzillet C, Tijeras-Raballand A, Ragulan C, et al: Inter- and intra-tumoural heterogeneity in cancer-associated fibroblasts of human pancreatic ductal adenocarcinoma. J Pathol 248:51-65, 2019

23. Dong D, Jia L, Zhang L, et al: Periostin and CA242 as potential diagnostic serum biomarkers complementing CA19.9 in detecting pancreatic cancer. Cancer Science 109:2841-2851, 2018

24. Natale F, Vivo M, Falco G, et al: Deciphering DNA methylation signatures of pancreatic cancer and pancreatitis. Clinical Epigenetics 11:1-12, 2019

25. Hasan S, Jacob R, Manne U, et al: Advances in pancreatic cancer biomarkers. Oncology reviews 13, 2019

26. Kisiel JB, Raimondo M, Taylor WR, et al: New DNA Methylation Markers for Pancreatic Cancer: Discovery, Tissue Validation, and Pilot Testing in Pancreatic Juice. Clinical Cancer Research 21:4473-4481, 2015

27. Fransson S, Martinsson T, Ejeskär K: Neuroblastoma tumors with favorable and unfavorable outcomes: Significant differences in mRNA expression of genes mapped at 1p36.2. Genes, Chromosomes and Cancer 46:45-52, 2007

28. Bodelon C, Oh H, Chatterjee N, et al: Association between breast cancer genetic susceptibility variants and terminal duct lobular unit involution of the breast. International Journal of Cancer 140:825-832, 2017

29. Kattan WE, Liu J, Montufar-Solis D, et al: Components of the phosphatidylserine endoplasmic reticulum to plasma membrane transport mechanism as targets for KRAS inhibition in pancreatic cancer. Proceedings of the National Academy of Sciences 118:e2114126118, 2021

30. Zhang Y, Ji S, Zhang X, et al: Human CPTP promotes growth and metastasis via sphingolipid metabolite ceramide and PI4KA/AKT signaling in pancreatic cancer cells. Int J Biol Sci 18:4963-4983, 2022

31. Adhikari H, Kattan WE, Kumar S, et al: Oncogenic KRAS is dependent upon an EFR3A-PI4KA signaling axis for potent tumorigenic activity. Nature Communications 12:5248, 2021
