# Supplementary material for: Mapping the Proteomic Landscape of Pancreatic Cancer: Prognostic Insights and Subtype Stratification
Source: Cancer Res Commun. 2025 Oct 23;5(10):1879–93. doi: 10.1158/2767-9764.CRC-25-0229 (PMC12548992; doi:10.1158/2767-9764.CRC-25-0229)
Supplement: Supplementary Table 5 — shows the distribution of clinical variables in the study cohort across the two proteomic-based risk score groups. Gln61Arg: Glutamine to Arginine at position 61, Gln61His: Glutamine to Histidine at position 61, Gly12Ala: Glycine to Alanine at position 12, Gly12Arg: Glycine to Arginine at position 12, Gly12Asp: Glycine to Aspartic Acid at position 12, Gly12Cys: Glycine to Cysteine at position 12, Gly12Leu: Glycine to Leucine at position 12, Gly12Val: Glycine to Valine at position 12. Note that all patients showed positivity for COSMIC signature 1, while no patients showed positivity for COSMIC signatures 6, 20, 25, and 26. For COSMIC signatures 13, 18, 28, and 30, only one patient was in the positive group. [file crc-25-0229_supplementary_table_5_suppst5.docx]

**Supplementary Table 5: Clinical variables across the proteomic-based risk groups**

| Clinical Variables | | High Risk | Low Risk | p-value |
| --- | --- | --- | --- | --- |
|  |  | N = 94 | N = 21 |  |
| KRAS Status | KRAS Mutation Found | 76 (81%) | 12 (57%) | 0.02 |
|  | No KRAS Mutation Found | 8 (9%) | 6 (29%) |  |
|  | Unknown | 10 (11%) | 3 (14%) |  |
| HRD status | Negative | 44 (47%) | 11 (52%) | 1 |
|  | Positive | 4 (4%) | 0 (0%) |  |
|  | Unknown | 46 (49%) | 10 (48%) |  |
| COSMIC Signature 2 | Negative | 42 (45%) | 11 (52%) | 0.58 |
|  | Positive | 6 (6%) | 0 (0%) |  |
|  | Unknown | 46 (49%) | 10 (48%) |  |
| COSMIC Signature 3 | Negative | 38 (40%) | 11 (52%) | 0.18 |
|  | Positive | 10 (11%) | 0 (0%) |  |
|  | Unknown | 46 (49%) | 10 (48%) |  |
| COSMIC Signature 5 | Negative | 33 (35%) | 4 (19%) | 0.08 |
|  | Positive | 15 (16%) | 7 (33%) |  |
|  | Unknown | 46 (49%) | 10 (48%) |  |
| COSMIC Signature 8 | Negative | 15 (16%) | 4 (19%) | 0.73 |
|  | Positive | 33 (35%) | 7 (33%) |  |
|  | Unknown | 46 (49%) | 10 (48%) |  |
| COSMIC Signature 9 | Negative | 44 (47%) | 10 (48%) | 1 |
|  | Positive | 4 (4%) | 1 (5%) |  |
|  | Unknown | 46 (49%) | 10 (48%) |  |
| COSMIC Signature 16 | Negative | 38 (40%) | 8 (38%) | 0.69 |
|  | Positive | 10 (11%) | 3 (14%) |  |
|  | Unknown | 46 (49%) | 10 (48%) |  |
| COSMIC Signature 17 | Negative | 42 (45%) | 9 (43%) | 0.63 |
|  | Positive | 6 (6%) | 2 (10%) |  |
|  | Unknown | 46 (49%) | 10 (48%) |  |
| KRAS Subtypes | No Mutation | 18 (19%) | 9 (43%) | 0.53 |
|  | Gln61Arg | 1 (1%) | 0 (0%) |  |
|  | Gln61His | 3 (3%) | 1 (5%) |  |
|  | Gly12Ala | 1 (1%) | 0 (0%) |  |
|  | Gly12Arg | 13 (14%) | 3 (14%) |  |
|  | Gly12Asp | 34 (36%) | 5 (24%) |  |
|  | Gly12Cys | 2 (2%) | 0 (0%) |  |
|  | Gly12Leu | 1 (1%) | 0 (0%) |  |
|  | Gly12Val | 21 (22%) | 3 (14%) |  |

Supplementary Table 5 shows the distribution of clinical variables in the study cohort across the two proteomic-based risk score groups.
Gln61Arg: Glutamine to Arginine at position 61, Gln61His: Glutamine to Histidine at position 61, Gly12Ala: Glycine to Alanine at position 12, Gly12Arg: Glycine to Arginine at position 12, Gly12Asp: Glycine to Aspartic Acid at position 12, Gly12Cys: Glycine to Cysteine at position 12, Gly12Leu: Glycine to Leucine at position 12, Gly12Val: Glycine to Valine at position 12.

Note that all patients showed positivity for COSMIC signature 1, while no patients showed positivity for COSMIC signatures 6, 20, 25, and 26. For COSMIC signatures 13, 18, 28, and 30, only one patient was in the positive group.
