# Supplementary material for: Mapping the Proteomic Landscape of Pancreatic Cancer: Prognostic Insights and Subtype Stratification
Source: Cancer Res Commun. 2025 Oct 23;5(10):1879–93. doi: 10.1158/2767-9764.CRC-25-0229 (PMC12548992; doi:10.1158/2767-9764.CRC-25-0229)
Supplement: Supplementary Table 6 — shows the results of the univariate and multivariate Cox regression model with Stepwise-AIC, showing the association of each of the COSMIC signatures with overall survival in the study cohort. Note that all patients showed positivity for COSMIC signature 1, while no patients showed positivity for COSMIC signatures 6, 20, 25, and 26. For COSMIC signatures 13, 18, 28, and 30, only one patient was in the positive group. [file crc-25-0229_supplementary_table_6_suppst6.docx]

**Supplementary Table 6: Univariate and multivariate Cox regression model with Stepwise-AIC showing the association of each of the COSMIC signatures with overall survival.**

| **COSMIC Signatures** | **Univariate model** | | | | **Multivariate model with Step AIC** | | | |
| --- | --- | --- | --- | --- | --- | --- | --- | --- |
|  | **Hazard Ratio** | **Lower 95% CI** | **Upper 95% CI** | **P value** | **Hazard Ratio** | **Lower 95% CI** | **Upper 95% CI** | **P value** |
| **Sig2** | 1.31 | 0.56 | 3.09 | 0.532 | **3.47** | **1.23** | **9.77** | **0.019** |
| **Sig3** | **3.34** | **1.56** | **7.15** | **0.002** | **3.96** | **1.81** | **8.69** | **0.001** |
| **Sig5** | 0.71 | 0.41 | 1.25 | 0.241 | NA | NA | NA | NA |
| **Sig6** | NA | NA | NA | NA | NA | NA | NA | NA |
| **Sig8** | 1.29 | 0.71 | 2.36 | 0.401 | 1.65 | 0.83 | 3.29 | 0.152 |
| **Sig9** | 1.63 | 0.64 | 4.14 | 0.307 | NA | NA | NA | NA |
| **Sig13** | 0.43 | 0.06 | 3.18 | 0.411 | 0.21 | 0.02 | 1.85 | 0.158 |
| **Sig16** | 1.29 | 0.66 | 2.51 | 0.458 | NA | NA | NA | NA |
| **Sig17** | 0.67 | 0.28 | 1.58 | 0.362 | NA | NA | NA | NA |
| **Sig18** | 3.17 | 0.42 | 23.88 | 0.264 | 6.94 | 0.82 | 58.78 | 0.076 |
| **Sig20** | NA | NA | NA | NA | NA | NA | NA | NA |
| **Sig25** | NA | NA | NA | NA | NA | NA | NA | NA |
| **Sig26** | NA | NA | NA | NA | NA | NA | NA | NA |
| **Sig28** | 0.8 | 0.11 | 5.84 | 0.827 | NA | NA | NA | NA |
| **Sig30** | 0.8 | 0.11 | 5.84 | 0.827 | NA | NA | NA | NA |

Supplementary Table 6 shows the results of the univariate and multivariate Cox regression model with Stepwise-AIC, showing the association of each of the COSMIC signatures with overall survival in the study cohort. Note that all patients showed positivity for COSMIC signature 1, while no patients showed positivity for COSMIC signatures 6, 20, 25, and 26. For COSMIC signatures 13, 18, 28, and 30, only one patient was in the positive group.
