# Supplementary material for: Mapping the Proteomic Landscape of Pancreatic Cancer: Prognostic Insights and Subtype Stratification
Source: Cancer Res Commun. 2025 Oct 23;5(10):1879–93. doi: 10.1158/2767-9764.CRC-25-0229 (PMC12548992; doi:10.1158/2767-9764.CRC-25-0229)
Supplement: Supplementary Table 7 — shows the list of differentially abundant proteins (DAP) and their associated biological pathways, as well as the potential drug targets among each COSMIC signature of interest. Note that no patients showed positivity for COSMIC signatures 1,6,20,25,26, while COSMIC signatures 13, 18, 28, and 30 had only one patient in the positive group. [file crc-25-0229_supplementary_table_7_suppst7.docx]

**Supplementary Table 7: List of DAP, pathways, and potential drug targets within COSMIC signatures of interest.**

| **COSMIC**  **Signature** | **List of top 10 up regulated** | **Top pathways** | **FDA/potential drug targets (among top 100 upregulated proteins)** |
| --- | --- | --- | --- |
| **Sig2 (n=6)** | IGHV3OR16-12, IFT20, CRLF3, IGLV3-9, CASP10, SPATA5, HLA-DQA1, ATF7IP, C19orf12, SAMD9L | Immunoglobulin production, RNA splicing pathways, epithelial cell differentiation and maintenance | AMACR, CASP10, COQ7, EPHB4, SPATA5 |
| **Sig3 (n=10)** | NAMPT, ITGAM, MNDA, GCA, FCGR3A, LCN2, SOD2, CSTA, LTF, NCF4 | Neutrophil degranulation, Extracellular matrix organization, immune response (defence response to bacterium, neutrophil extracellular trap formation, innate immune response, and complement system) | CDA, ELANE, FCGR3A, FN1, MMP9, SERPINE1, CASP14, GYS2, HTRA1, LOXL3, MPO, PDAI4, PLOD2, PRTN3, PXDN, SLC2A1, SOD2 |
| **Sig5 (n=23)** | RNASEH2A, CELA2A, CTRC, SLC17A5, ZG16, PRSS2, MRPL21, IGHV1-24, TOGARAM1, PNLIP | Protein digestion and pancreatic secretion | PNLIP, CELA2A, CLDN3, CTRC, PRSS1, RNASEH2A, SLC17A5 |
| **Sig8 (n= 42)** | IGHV1-69D, IGHV1-3, NEO1, MZT1, MUC5B, EDC3, TTC3, DSG1, SLC4A2, IGHV1-24 | Axonogenesis, adaptive immune response, and metabolic pathways (response to ketone, alcohol metabolism and lipid transport) | AKR1C2, F5, PNPLA8, SPAST |
| **Sig9 (n=5)** | DUOX2, LGALS8, RAB25, AIM2, CALML5, ZC3H14, TRAPPC9, PTRH1, RAB2B, CHKB | Response to virus, positive regulation of defense response, neutrophil degranulation, regulation of vesicle-mediated transport, and positive regulation of cell motility | CHKB, DUOX2, PRTN3 |
| **Sig16 (n=13)** | PHIP, WDR36, SLC9B2, SLC29A1, TMA16, WAS, LPGAT1, ZFAND1, THAP1, CD209 | Metabolic pathways (fatty acid and lipoprotein transport, and regulation of protein transport) | CDK12, LOXL3, OTUD6B |
| **Sig17 (n=8)** | IGHV3OR16-12, CNTRL, OTC, TIMMDC1, KDM4B, ANKRD27, EIF1AY, CDKN2C, ZC3H14, TGAL | Cell adhesion, and nuclear receptor meta-pathway | ITGAL, DUOX2, OTC, SLC20A2 |

Supplementary Table 7 shows the list of differentially abundant proteins (DAP) and their associated biological pathways, as well as the potential drug targets among each COSMIC signature of interest. Note that no patients showed positivity for COSMIC signatures 1,6,20,25,26, while COSMIC signatures 13, 18, 28, and 30 had only one patient in the positive group.
