# Supplementary material for: Mapping the Proteomic Landscape of Pancreatic Cancer: Prognostic Insights and Subtype Stratification
Source: Cancer Res Commun. 2025 Oct 23;5(10):1879–93. doi: 10.1158/2767-9764.CRC-25-0229 (PMC12548992; doi:10.1158/2767-9764.CRC-25-0229)
Supplement: Supplementary Table 8 — shows the differential abundance of the S100 protein family across the different clinical variables of interest in the study cohort, where “Up” means upregulated and “Down” means downregulated in the group of interest versus the other group. *Note that S100 A1, S100A13, and S100A3 were not differentially abundant among any subgroup. [file crc-25-0229_supplementary_table_8_suppst8.docx]

**Supplementary Table 8: Differential abundance of the S100 protein family across different clinical variables**

| **S100 Proteins*** | **S100A10** | **S100A11** | **S100A12** | **S100A14** | **S100A16** | **S100A2** | **S100A4** | **S100A6** | **S100A7** | **S100A7L2** | **S100A8** | **S100A9** | **S100B** | **S100P** |
| --- | --- | --- | --- | --- | --- | --- | --- | --- | --- | --- | --- | --- | --- | --- |
| Tumor vs normal | Up | Up | ---- | ---- | ---- | ---- | Up | Up | ---- | ---- | ---- | ---- | Down | Up |
| Dead vs alive | ---- | ---- | Up | ---- | ---- | ---- | ---- | ---- | ---- | ---- | ---- | ---- | Down | Up |
| Tumor location (Body/tail vs Head) | ---- | ---- | ---- | ---- | ---- | Up | ---- | ---- | ---- | ---- | ---- | ---- | ---- | ---- |
| Recurrence Yes vs no | ---- | ---- | ---- | ---- | ---- | ---- | ---- | ---- | ---- | ---- | ---- | ---- | ---- | Up |
| Grade high vs low | ---- | ---- | ---- | ---- | ---- | Up | ---- | ---- | ---- | ---- | Up | Up | ---- | ---- |
| HRD yes vs no | ---- | ---- | Up | ---- | ---- | Up | ---- | ---- | ---- | ---- | Up | Up | ---- | ---- |
| Signature 3 Yes vs no | ---- | ---- | Up | ---- | ---- | Up | ---- | ---- | ---- | ---- | Up | Up | ---- | ---- |
| Signature 8 yes vs no | ---- | ---- | ---- | ---- | ---- | ---- | ---- | ---- | Down | ---- | ---- | ---- | ---- | ---- |
| Signature 9 yes vs no | ---- | ---- | ---- | Up | ---- | ---- | ---- | ---- | ---- | ---- | ---- | ---- | ---- | ---- |
| Clusters 2,4 worst vs 1,3 best | ---- | ---- | ---- | ---- | ---- | ---- | ---- | ---- | ---- | ---- | Up | ---- | Down | Up |
| KRAS, Mut vs Wild | ---- | ---- | ---- | ---- | ---- | ---- | ---- | ---- | ---- | ---- | ---- | ---- | ---- | ---- |
| KRASG12A  Mut versus others G12 | ---- | ---- | ---- | Up | Up | ---- | ---- | ---- | ---- | ---- | ---- | ---- | ---- | ---- |
| KRASG12D  Mut versus others G12 | Down | ---- | ---- | ---- | ---- | ---- | ---- | ---- | ---- | ---- | ---- | ---- | ---- | ---- |
| KRASG12C  Mut versus others G12 | ---- | ---- | ---- | ---- | ---- | Up | ---- | ---- | Up | Up | ---- | ---- | ---- | ---- |

Supplementary Table 8 shows the differential abundance of the S100 protein family across the different clinical variables of interest in the study cohort, where “Up” means upregulated and “Down” means downregulated in the group of interest versus the other group. *Note that S100 A1, S100A13, and S100A3 were not differentially abundant among any subgroup.
