# Supplementary material for: Mapping the Proteomic Landscape of Pancreatic Cancer: Prognostic Insights and Subtype Stratification
Source: Cancer Res Commun. 2025 Oct 23;5(10):1879–93. doi: 10.1158/2767-9764.CRC-25-0229 (PMC12548992; doi:10.1158/2767-9764.CRC-25-0229)
Supplement: Supplementary Table 9 — shows the differential abundance of the KRT protein family across the different clinical variables of interest, where “Up” means upregulated and “Down” means downregulated in the group of interest versus others. *Note that KRT9, KRT10, KRT20, KRT23, KRT77, KRT74, KRT79 and KRT80 were not differentially abundant among any subgroup [file crc-25-0229_supplementary_table_9_suppst9.docx]

**Supplementary Table 9: Differential abundance of the KRT protein family across different clinical variables**

| **KRT protein*** | **KRT1** | **KRT2** | **KRT4** | **KRT5** | **KRT6A** | **KRT6B** | **KRT7** | **KRT8** | **KRT13** | **KRT14** | **KRT15** | **KRT16** | **KRT17** | **KRT18** | **KRT19** | **KRT24** | **KRT35** | **KRT72** | **KRT73** | **KRT75** | **KRT85** |
| --- | --- | --- | --- | --- | --- | --- | --- | --- | --- | --- | --- | --- | --- | --- | --- | --- | --- | --- | --- | --- | --- |
| Tumor vs normal | ---- | ---- | ---- | ---- | Up | ---- | Up | ---- | ---- | ---- | ---- | Down | Up | ---- | Up | ---- | Down | Up | ---- | ---- | Up |
| Dead vs alive | ---- | ---- | ---- | ---- |  | Down | Up | Up | ---- | ---- | ---- | ---- |  | Up | Up | ---- | ---- | ---- | ---- | ---- | Up |
| Tumor location (Body/tail vs Head) | ---- | ---- | ---- | Up | Up | ---- | ---- | ---- | Up | ---- | ---- | ---- | ---- | ---- | ---- | ---- | ---- | ---- | ---- | Down | ---- |
| Recurrence Y vs N | ---- | ---- | ---- | ---- | ---- | ---- | Up | Up | ---- | ---- | ---- | ---- | ---- | Up | Up | ---- | ---- | ---- | ---- | ---- | ---- |
| Grade High vs low | ---- | ---- | ---- |  | Up | ---- | ---- | ---- | Up | ---- | ---- | Up | Up | ---- | ---- | Down | Down | Up | ---- | Down | ---- |
| HRD Y vs N | ---- | ---- | ---- | Up | Up | Up | ---- | ---- | ---- | ---- | ---- | Up | ---- | ---- | ---- | ---- | ---- | ---- | Up | ---- | ---- |
| Sig 3 | ---- | ---- | ---- | Up | Up | Up | ---- | ---- | Up | ---- | Up | Up | Up | ---- | ---- | ---- | ---- | ---- | ---- | ---- | ---- |
| Sig 2 | ---- | ---- | ---- | ---- | ---- | ---- | ---- | ---- | ---- | ---- | ---- | ---- | ---- | ---- | ---- | ---- | Up | ---- | ---- | ---- | ---- |
| Sig5 | ---- | ---- | ---- | ---- | ---- | ---- | ---- | ---- | Down | ---- | ---- | ---- | ---- | ---- | ---- | ---- | Down | ---- | ---- | ---- | ---- |
| Sig9 | ---- | ---- | Down | ---- | ---- | ---- | ---- | ---- | ---- | ---- | ---- | ---- | ---- | ---- | ---- | ---- | ---- | ---- | ---- | ---- | ---- |
| Sig16 | ---- | ---- | ---- | ---- | ---- | ---- | ---- | Down | ---- | ---- | ---- | ---- | ---- | ---- | ---- | ---- | ---- | ---- | ---- | ---- | ---- |
| Sig17 | ---- | ---- | ---- | ---- | ---- | ---- | ---- | ---- | ---- | ---- | ---- | ---- | ---- | ---- | ---- | ---- | ---- | Up | ---- | ---- | Up |
| KRAS | ---- | ---- | ---- | ---- | ---- | ---- | ---- | ---- | ---- | ---- | ---- | ---- | ---- | ---- | Up | ---- | ---- |  | ---- | ---- | ---- |
| KRASG12R | ---- | ---- | ---- | Down | ---- | ---- | ---- | ---- | ---- | ---- | ---- | Down | ---- | ---- | ---- | ---- | ---- | ---- | ---- | ---- | ---- |
| KRASG12C | Down | Down | ---- | Up | Up | ---- | Down | ---- | ---- | Up | Up | Up | ---- | ---- | ---- | ---- | ---- | ---- | Down | ---- | Down |

Supplementary Table 9 shows the differential abundance of the KRT protein family across the different clinical variables of interest, where “Up” means upregulated and “Down” means downregulated in the group of interest versus others. *Note that KRT9, KRT10, KRT20, KRT23, KRT77, KRT74, KRT79 and KRT80 were not differentially abundant among any subgroup
