# Supplementary material for: Mapping the Proteomic Landscape of Pancreatic Cancer: Prognostic Insights and Subtype Stratification
Source: Cancer Res Commun. 2025 Oct 23;5(10):1879–93. doi: 10.1158/2767-9764.CRC-25-0229 (PMC12548992; doi:10.1158/2767-9764.CRC-25-0229)
Supplement: Supplementary Figure 1 — shows the main pathways associated with lists of proteins of interest. (A) Summary of Enrichment analysis in DisGeNET for the 20-protein panel, showing a highly significant association with pancreatic neoplasm. (B) Summary of the main pathways associated with proteins uniquely identified when restricting the differential abundance analysis to samples with ≥ 50% cancer content. (C) Summary of the main pathways associated with proteins uniquely identified to be related to pancreatic tumor cells after subtracting the list of proteins associated with fibroblasts and collagen deposition. All the figure was generated from the Metascape website: (https://metascape.org/gp/index.html#/main/step1). [file crc-25-0229_supplementary_figure_1_suppsf1.pptx]

## Slide 1
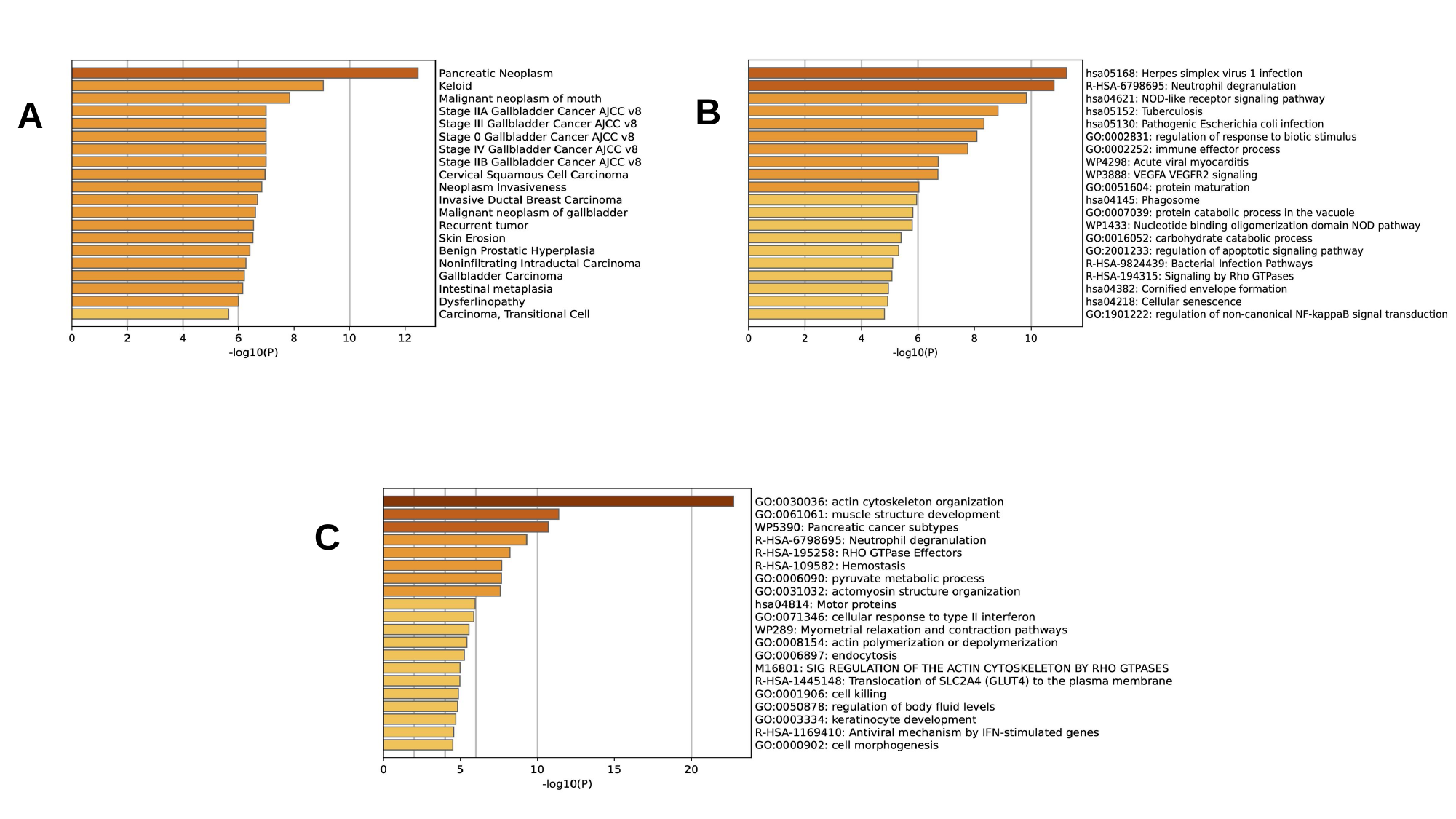

A
B
C

## Slide 2
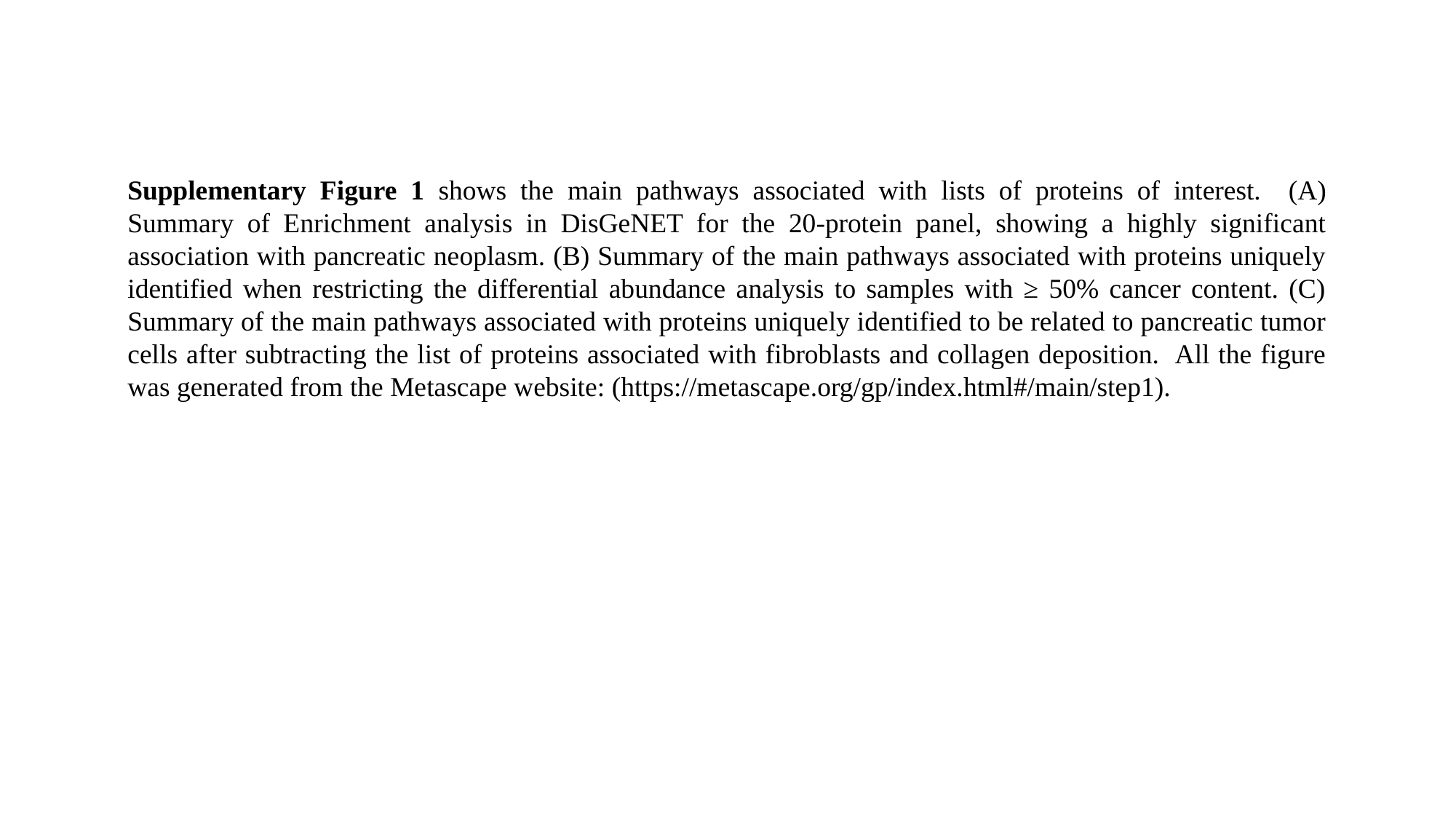

Supplementary Figure 1 shows the main pathways associated with lists of proteins of interest. (A) Summary of Enrichment analysis in DisGeNET for the 20-protein panel, showing a highly significant association with pancreatic neoplasm. (B) Summary of the main pathways associated with proteins uniquely identified when restricting the differential abundance analysis to samples with ≥ 50% cancer content. (C) Summary of the main pathways associated with proteins uniquely identified to be related to pancreatic tumor cells after subtracting the list of proteins associated with fibroblasts and collagen deposition. All the figure was generated from the Metascape website: (https://metascape.org/gp/index.html#/main/step1).
