# Supplementary material for: Mapping the Proteomic Landscape of Pancreatic Cancer: Prognostic Insights and Subtype Stratification
Source: Cancer Res Commun. 2025 Oct 23;5(10):1879–93. doi: 10.1158/2767-9764.CRC-25-0229 (PMC12548992; doi:10.1158/2767-9764.CRC-25-0229)
Supplement: Supplementary Figure 2 — shows figures related to the Consensus clustering analyses. (A) A delta area plot displays the relative change in the cumulative distribution function (CDF) curve comparing k and k-1 clusters from our cohort. (B) The silhouette plot displays the silhouette width for the four clusters determined by consensus clustering within this cohort. (C) Kaplan-Meier plot of the recurrence-free survival rates of the combined clusters (Clusters 1 with 3 and Clusters 2 with 4). [file crc-25-0229_supplementary_figure_2_suppsf2.pdf]

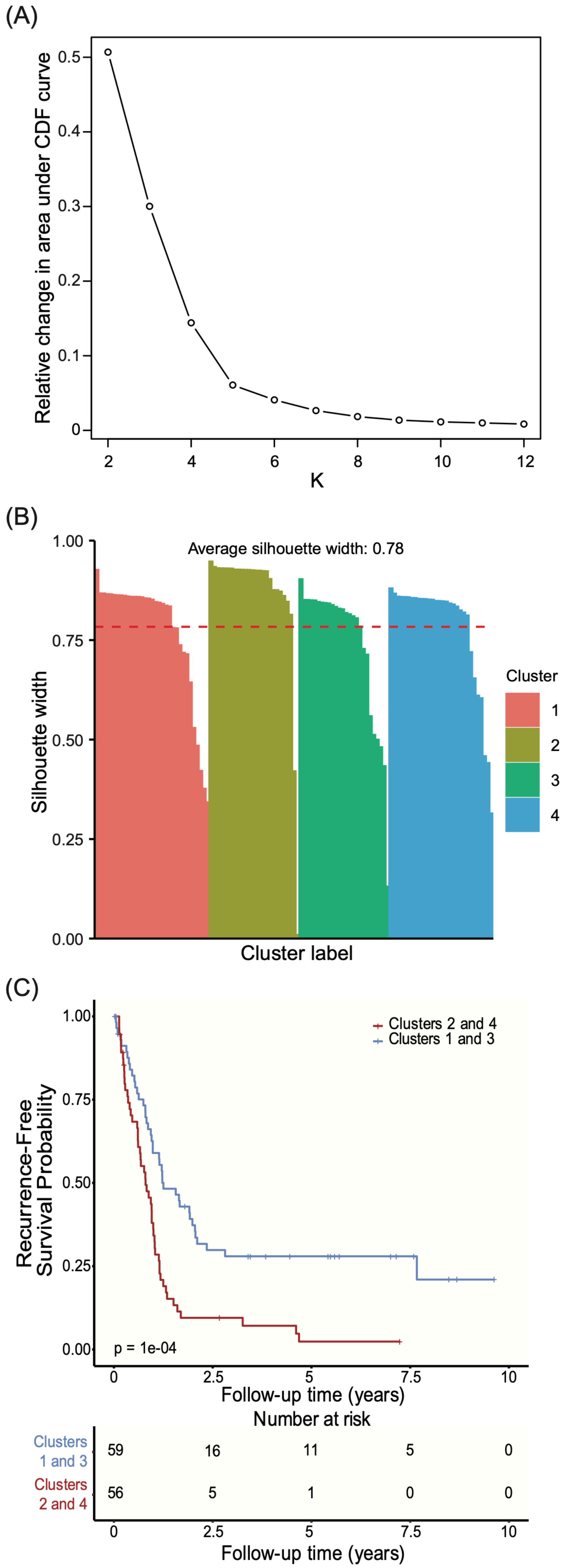

(A)
Relative change in area under CDF curve
0.5
0.4
0.3
0.2
0.1
0
2
4
6
8
10
12
K
(B)
Average silhouette width: 0.78
Silhouette width
1.00
0.75
0.50
0.25
0.00
Cluster label
Cluster
1
2
3
4
(C)
Clusters 2 and 4
Clusters 1 and 3
Recurrence-Free Survival Probability
1.00
0.75
0.50
0.25
0.00
p = 1e-04
0
2.5
5
7.5
10
Follow-up time (years)
Number at risk
Clusters 1 and 3
59
16
11
5
0
Clusters 2 and 4
56
5
1
0
0
0
2.5
5
7.5
10
Follow-up time (years)

Supplementary Figure 2 shows figures related to the Consensus clustering analyses. **(A)** A delta area plot displays the relative change in the cumulative distribution function (CDF) curve comparing k and k-1 clusters from our cohort. **(B)** The silhouette plot displays the silhouette width for the four clusters determined by consensus clustering within this cohort. **(C)** Kaplan-Meier plot of the recurrence-free survival rates of the combined clusters (Clusters 1 with 3 and Clusters 2 with 4).
