# Supplementary material for: Mapping the Proteomic Landscape of Pancreatic Cancer: Prognostic Insights and Subtype Stratification
Source: Cancer Res Commun. 2025 Oct 23;5(10):1879–93. doi: 10.1158/2767-9764.CRC-25-0229 (PMC12548992; doi:10.1158/2767-9764.CRC-25-0229)
Supplement: Supplementary Figure 3 — shows differential abundance and pathway enrichment analyses of clusters. (A) Volcano plot displaying the differentially expressed proteins between PDA samples classified between groups 1 and 3 against groups 2 and 4. (B) Pathways enriched in KEGG, Reactome, and Wikipathways for proteins upregulated in samples from clusters 2 and 4 compared to clusters 1 and 3. [file crc-25-0229_supplementary_figure_3_suppsf3.pdf]

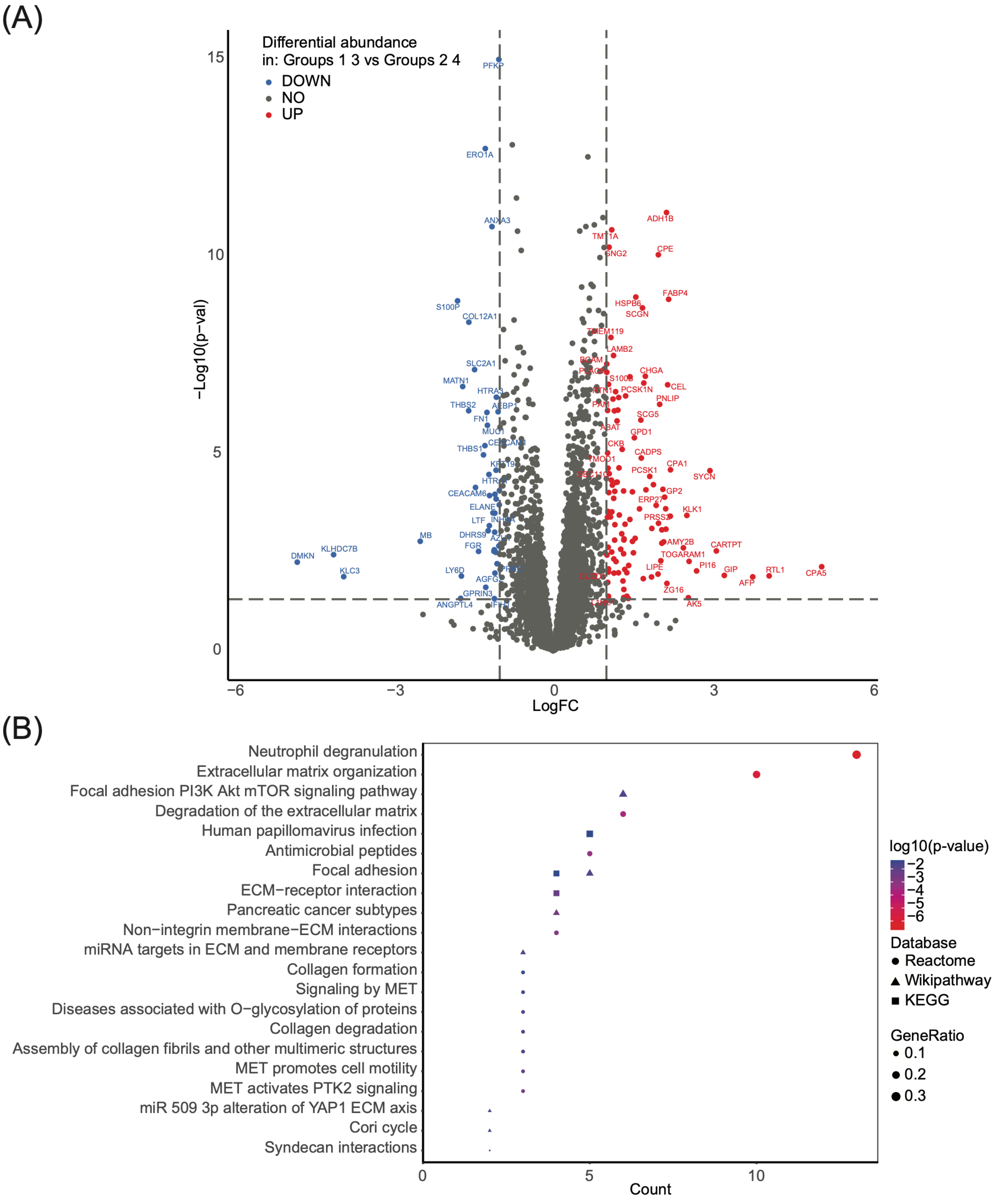
(A)
Differential abundance
in: Groups 1 3 vs Groups 2 4
DOWN
NO
UP
−Log10(p−val)
LogFC
PFKP
ERO1A
ANXA3
S100P
COL12A1
SLC2A1
MATN1
THBS2
THBS1
CEACAM6
DMKN
KLHDC7B
KLC3
MB
LY6D
ANGPTL4
ADH1B
TMT1A
GNG2
CPE
FABP4
HSPB6
SCGN
TMEM119
LAMB2
CHGA
CEL
PCSK1N
PNLIP
SCG5
GPD1
CKB
CADPS
CPA1
SYCN
PCSK1
GP2
KLK1
AMY2B
CARTPT
TOGARAM1
PI16
GIP
RTL1
AFP
CPA5
LIPE
ZG16
AK5
(B)
Neutrophil degranulation
Extracellular matrix organization
Focal adhesion PI3K Akt mTOR signaling pathway
Degradation of the extracellular matrix
Human papillomavirus infection
Antimicrobial peptides
Focal adhesion
ECM−receptor interaction
Pancreatic cancer subtypes
Non−integrin membrane−ECM interactions
miRNA targets in ECM and membrane receptors
Collagen formation
Signaling by MET
Diseases associated with O−glycosylation of proteins
Collagen degradation
Assembly of collagen fibrils and other multimeric structures
MET promotes cell motility
MET activates PTK2 signaling
miR 509 3p alteration of YAP1 ECM axis
Cori cycle
Syndecan interactions
Count
log10(p-value)
Database
Reactome
Wikipathway
KEGG
GeneRatio

Supplementary Figure 3 shows differential abundance and pathway enrichment analyses of clusters. **(A)** Volcano plot displaying the differentially expressed proteins between PDA samples classified between groups 1 and 3 against groups 2 and 4. **(B)** Pathways enriched in KEGG, Reactome, and Wikipathways for proteins upregulated in samples from clusters 2 and 4 compared to clusters 1 and 3.
