# Supplementary material for: Mapping the Proteomic Landscape of Pancreatic Cancer: Prognostic Insights and Subtype Stratification
Source: Cancer Res Commun. 2025 Oct 23;5(10):1879–93. doi: 10.1158/2767-9764.CRC-25-0229 (PMC12548992; doi:10.1158/2767-9764.CRC-25-0229)
Supplement: Supplementary Figure 4 — shows the Kaplan-Meier survival curves for each of the 18 proteins within the risk score. Median cut-off was used to dichotomize patients into two groups. [file crc-25-0229_supplementary_figure_4_suppsf4.pdf]

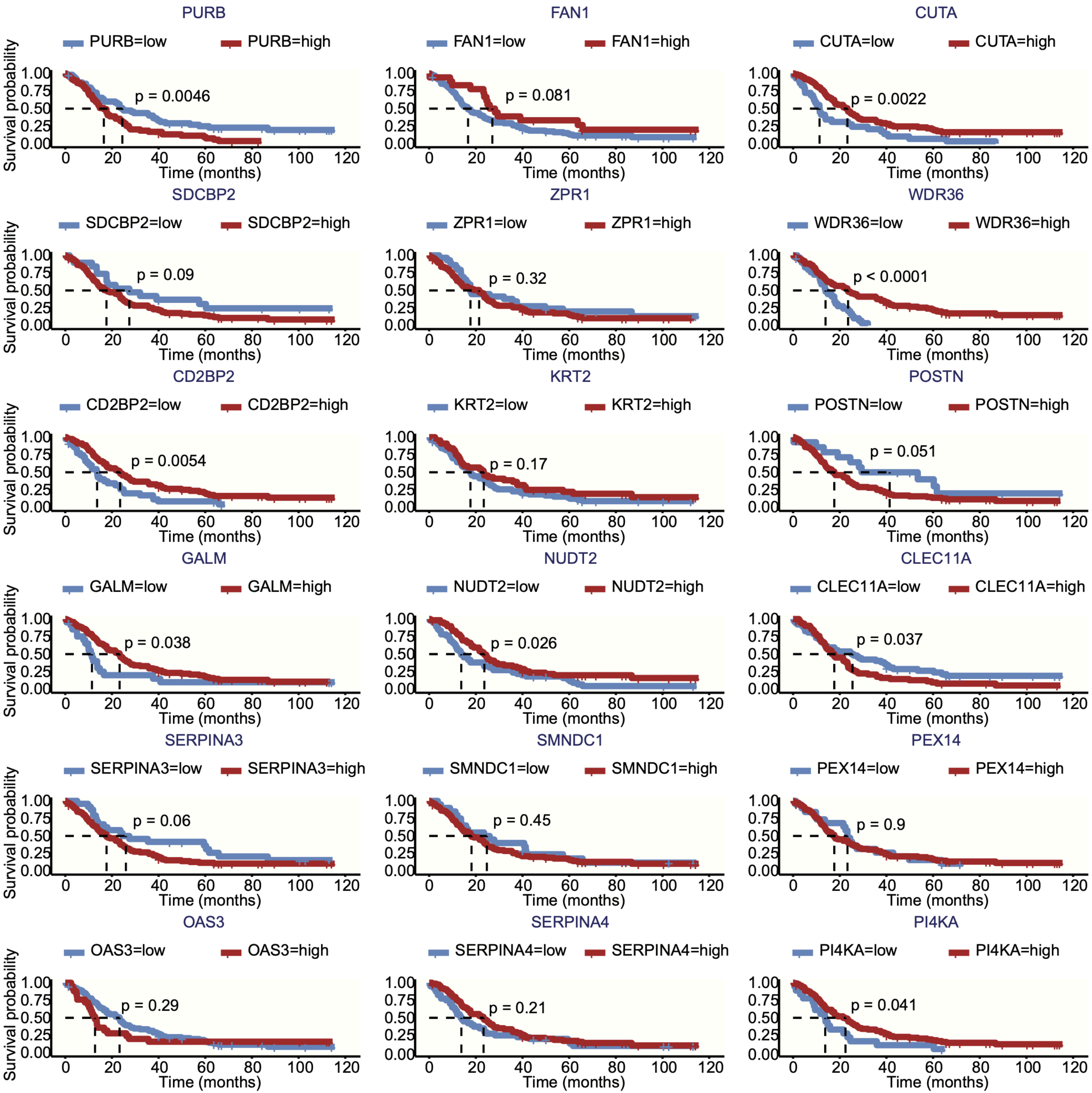
PURB
PURB=low
PURB=high
p = 0.0046
FAN1
FAN1=low
FAN1=high
p = 0.081
CUTA
CUTA=low
CUTA=high
p = 0.0022
SDCBP2
SDCBP2=low
SDCBP2=high
p = 0.09
ZPR1
ZPR1=low
ZPR1=high
p = 0.32
WDR36
WDR36=low
WDR36=high
p < 0.0001
CD2BP2
CD2BP2=low
CD2BP2=high
p = 0.0054
KRT2
KRT2=low
KRT2=high
p = 0.17
POSTN
POSTN=low
POSTN=high
p = 0.051
GALM
GALM=low
GALM=high
p = 0.038
NUDT2
NUDT2=low
NUDT2=high
p = 0.026
CLEC11A
CLEC11A=low
CLEC11A=high
p = 0.037
SERPINA3
SERPINA3=low
SERPINA3=high
p = 0.06
SMNDC1
SMNDC1=low
SMNDC1=high
p = 0.45
PEX14
PEX14=low
PEX14=high
p = 0.9
OAS3
OAS3=low
OAS3=high
p = 0.29
SERPINA4
SERPINA4=low
SERPINA4=high
p = 0.21
PI4KA
PI4KA=low
PI4KA=high
p = 0.041
Survival probability
1.00
0.75
0.50
0.25
0.00
Time (months)
0
20
40
60
80
100
120

Supplementary Figure 4 shows the Kaplan-Meier survival curves for each of the 18 proteins within the risk score. Median cut-off was used to dichotomize patients into two groups.
