# Supplementary material for: Mapping the Proteomic Landscape of Pancreatic Cancer: Prognostic Insights and Subtype Stratification
Source: Cancer Res Commun. 2025 Oct 23;5(10):1879–93. doi: 10.1158/2767-9764.CRC-25-0229 (PMC12548992; doi:10.1158/2767-9764.CRC-25-0229)
Supplement: Supplementary Figure 5 — shows the proteomic information for the 18 risk-score proteins. (A) Peptide detection rate within the dataset. (B) Protein Missingness in our tumor data only. (C) Protein Missingness in the CPTAC Pancreatic Cancer Cohort. [file crc-25-0229_supplementary_figure_5_suppsf5.pdf]

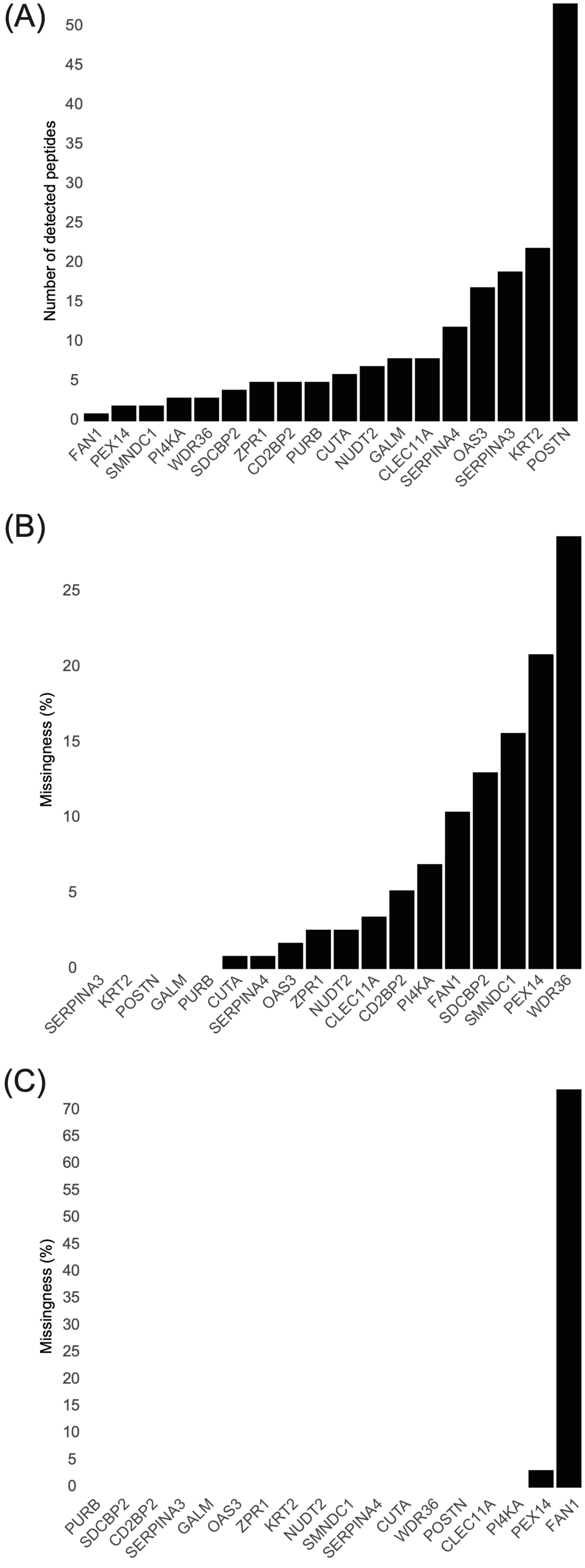
(A)
Number of detected peptides
0
5
10
15
20
25
30
35
40
45
50
FAN1
PEX14
SMNDC1
PI4KA
WDR36
SDCBP2
ZPR1
CD2BP2
PURB
CUTA
NUDT2
GALM
CLEC11A
SERPINA4
OAS3
SERPINA3
KRT2
POSTN
(B)
Missingness (%)
0
5
10
15
20
25
SERPINA3
KRT2
POSTN
GALM
PURB
CUTA
SERPINA4
OAS3
ZPR1
NUDT2
CLEC11A
CD2BP2
PI4KA
FAN1
SDCBP2
SMNDC1
PEX14
WDR36
(C)
Missingness (%)
0
5
10
15
20
25
30
35
40
45
50
55
60
65
70
PURB
SDCBP2
CD2BP2
SERPINA3
GALM
OAS3
ZPR1
KRT2
NUDT2
SMNDC1
SERPINA4
CUTA
WDR36
POSTN
CLEC11A
PI4KA
PEX14
FAN1

Supplementary Figure 5 shows the proteomic information for the 18 risk-score proteins. **(A)** Peptide detection rate within the dataset. **(B)** Protein Missingness in our tumor data only. **(C)** Protein Missingness in the CPTAC Pancreatic Cancer Cohort.
