# Supplementary material for: Mapping the Proteomic Landscape of Pancreatic Cancer: Prognostic Insights and Subtype Stratification
Source: Cancer Res Commun. 2025 Oct 23;5(10):1879–93. doi: 10.1158/2767-9764.CRC-25-0229 (PMC12548992; doi:10.1158/2767-9764.CRC-25-0229)
Supplement: Supplementary Figure 6 — shows a forest plot detailing the hazard ratio of the proteomic risk score and clinically relevant variables for PDA within our study cohort using multivariable Cox regression modeling. [file crc-25-0229_supplementary_figure_6_suppsf6.pdf]

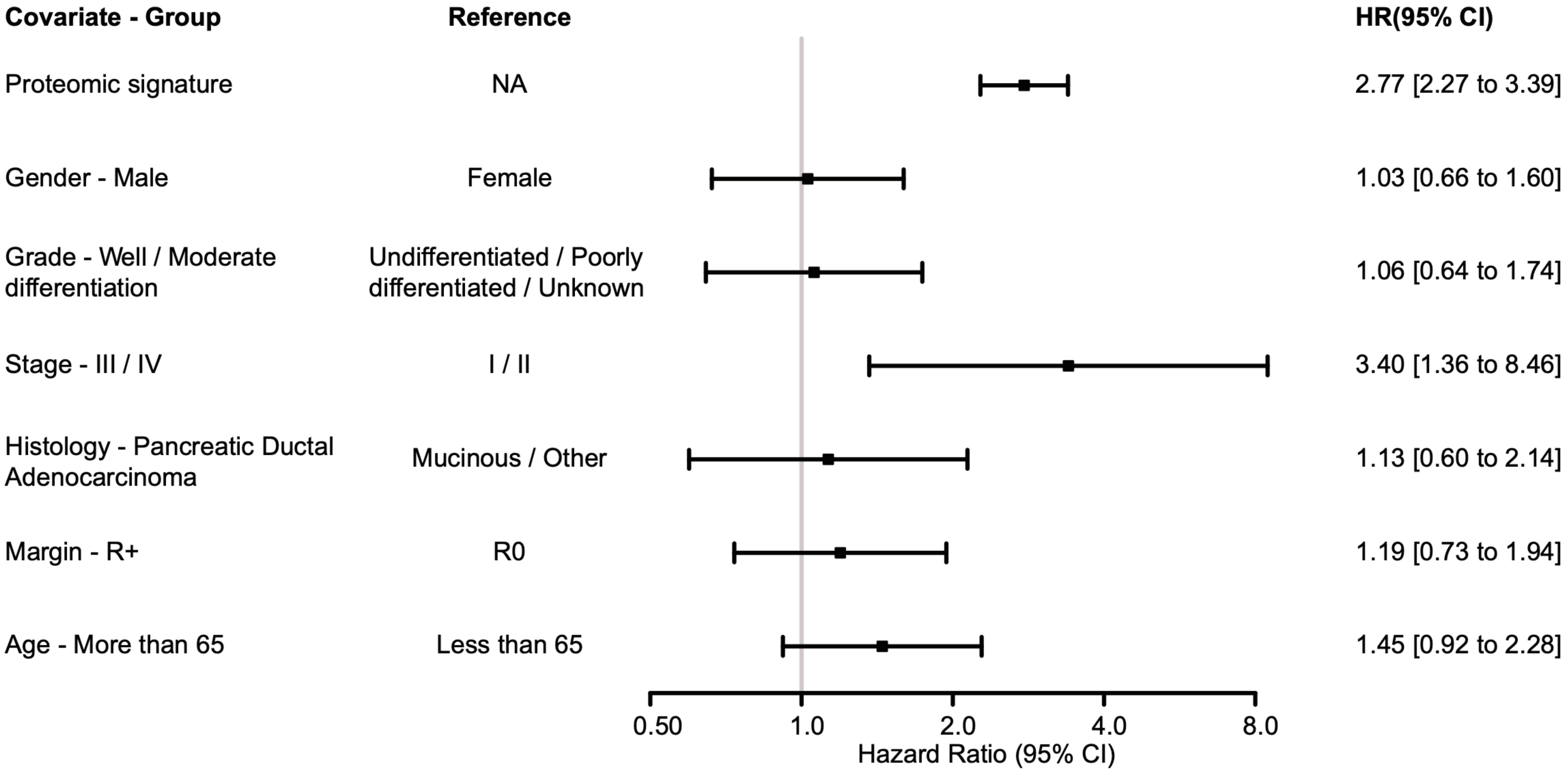
Covariate - Group
Reference
HR(95% CI)
Proteomic signature
NA
2.77 [2.27 to 3.39]
Gender - Male
Female
1.03 [0.66 to 1.60]
Grade - Well / Moderate differentiation
Undifferentiated / Poorly differentiated / Unknown
1.06 [0.64 to 1.74]
Stage - III / IV
I / II
3.40 [1.36 to 8.46]
Histology - Pancreatic Ductal Adenocarcinoma
Mucinous / Other
1.13 [0.60 to 2.14]
Margin - R+
R0
1.19 [0.73 to 1.94]
Age - More than 65
Less than 65
1.45 [0.92 to 2.28]
0.50
1.0
2.0
4.0
8.0
Hazard Ratio (95% CI)

Supplementary Figure 6 shows a forest plot detailing the hazard ratio of the proteomic risk score and clinically relevant variables for PDA within our study cohort using multivariable Cox regression modeling.
