# Supplementary material for: Mapping the Proteomic Landscape of Pancreatic Cancer: Prognostic Insights and Subtype Stratification
Source: Cancer Res Commun. 2025 Oct 23;5(10):1879–93. doi: 10.1158/2767-9764.CRC-25-0229 (PMC12548992; doi:10.1158/2767-9764.CRC-25-0229)
Supplement: Supplementary Figure 7 — shows the proteomic signature performance based on the Receiver operator characteristic curve analysis. (A) Receiver operator characteristic curve (ROC) at 1 year of follow-up for the proteomic signature (purple) and other clinically relevant variables for PDA within our cohort. (B) An Area Under the Curve (AUC) plot displays the AUC as a function of time for the proteomic risk score (purple) and other clinically relevant variables for PDA within our cohort. [file crc-25-0229_supplementary_figure_7_suppsf7.pdf]

(A)

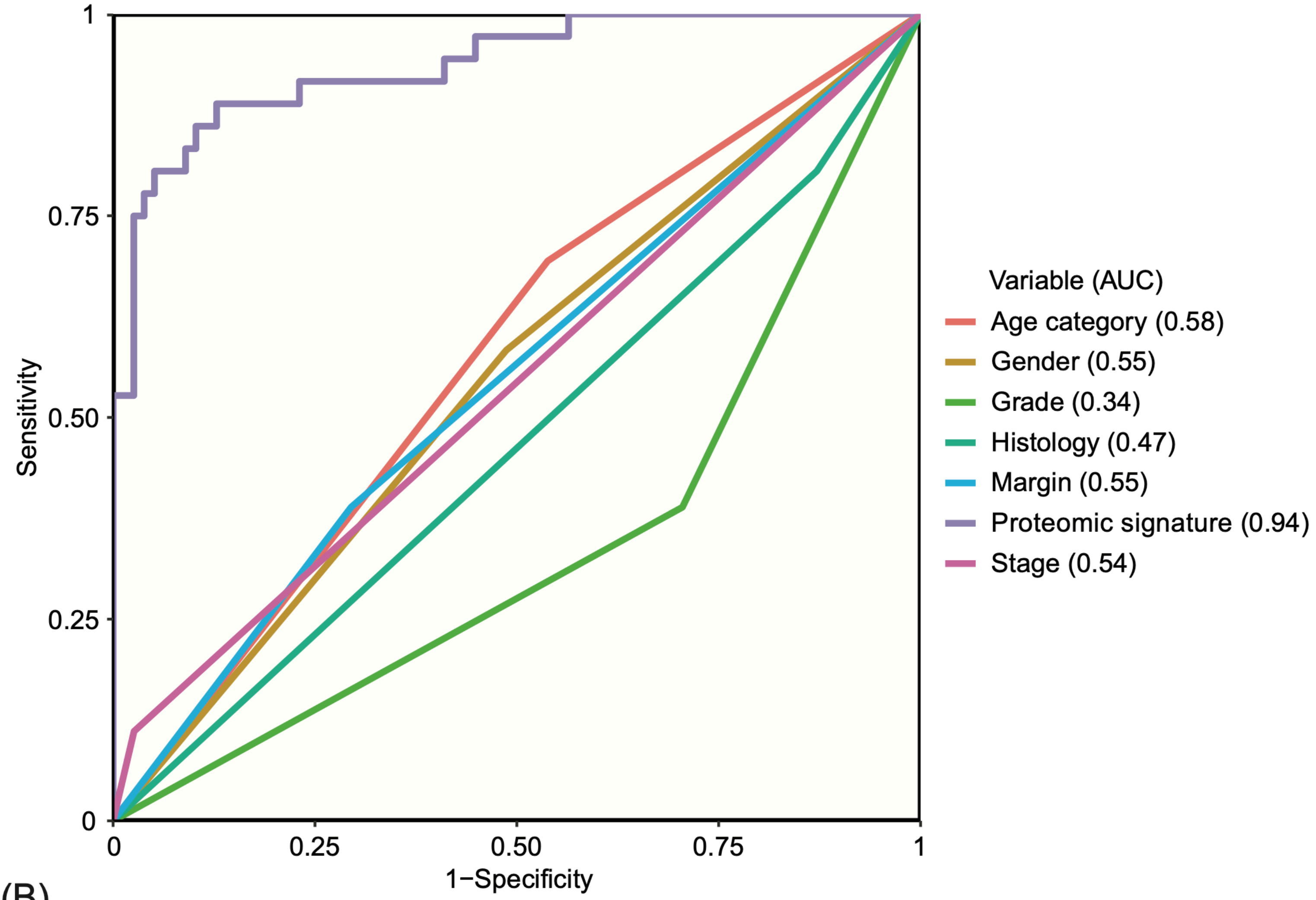
Sensitivity
1-Specificity
0
0.25
0.50
0.75
1
Variable (AUC)
Age category (0.58)
Gender (0.55)
Grade (0.34)
Histology (0.47)
Margin (0.55)
Proteomic signature (0.94)
Stage (0.54)

(B)

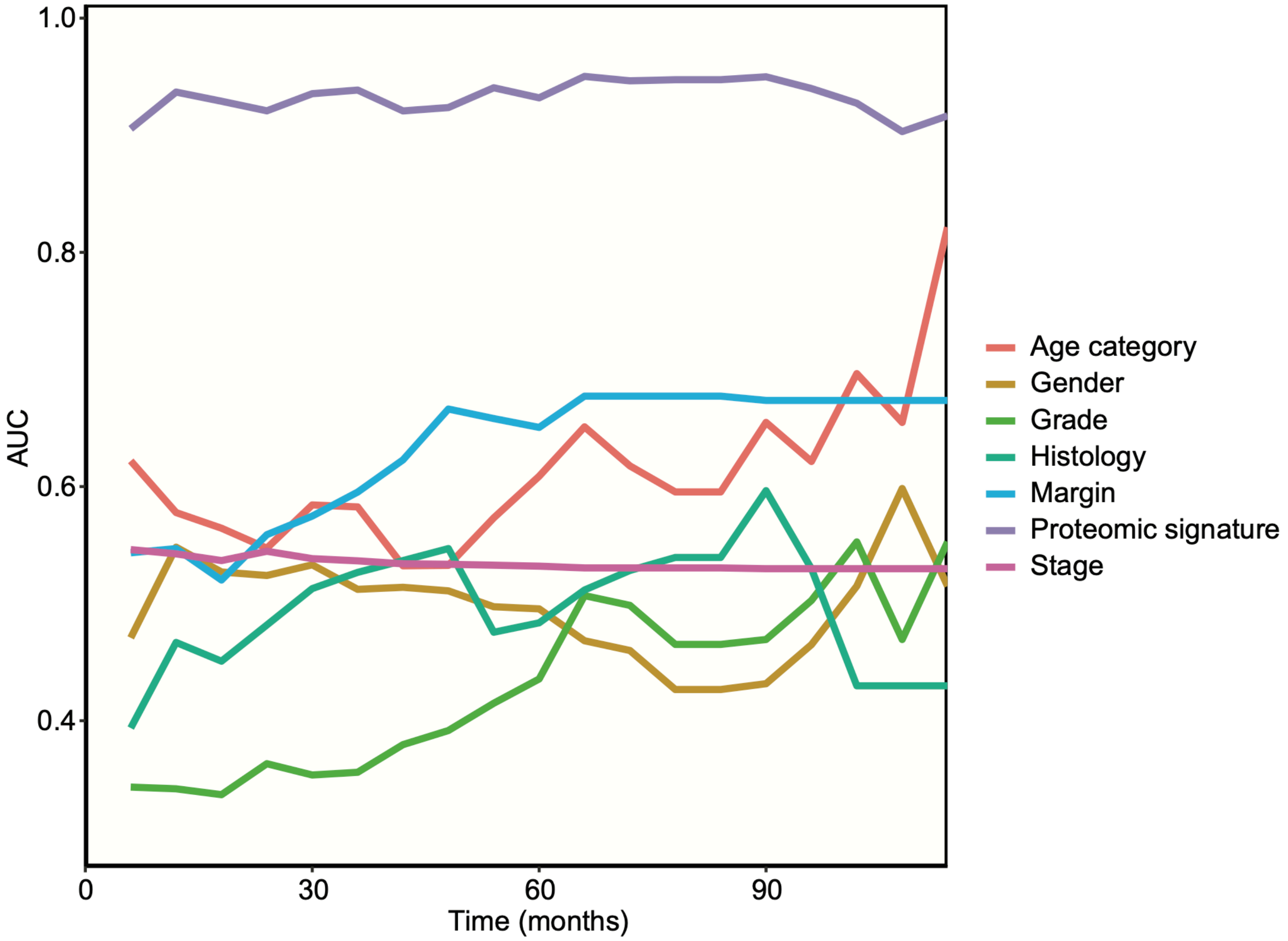
AUC
Time (months)
0.4
0.6
0.8
1.0
0
30
60
90
Age category
Gender
Grade
Histology
Margin
Proteomic signature
Stage

Supplementary Figure 7 shows the proteomic signature performance based on the Receiver operator characteristic curve analysis. **(A)** Receiver operator characteristic curve (ROC) at 1 year of follow-up for the proteomic signature (purple) and other clinically relevant variables for PDA within our cohort. **(B)** An Area Under the Curve (AUC) plot displays the AUC as a function of time for the proteomic risk score (purple) and other clinically relevant variables for PDA within our cohort.
