# Supplementary material for: Mapping the Proteomic Landscape of Pancreatic Cancer: Prognostic Insights and Subtype Stratification
Source: Cancer Res Commun. 2025 Oct 23;5(10):1879–93. doi: 10.1158/2767-9764.CRC-25-0229 (PMC12548992; doi:10.1158/2767-9764.CRC-25-0229)
Supplement: Supplementary Figure 8 — shows Kaplan-Meier curves displaying (A) the three-year survival for groups dichotomised by proteomic risk score in ProCan data. (B) The recurrence-free survival for groups dichotomized by proteomic risk score in ProCan data. [file crc-25-0229_supplementary_figure_8_suppsf8.pdf]

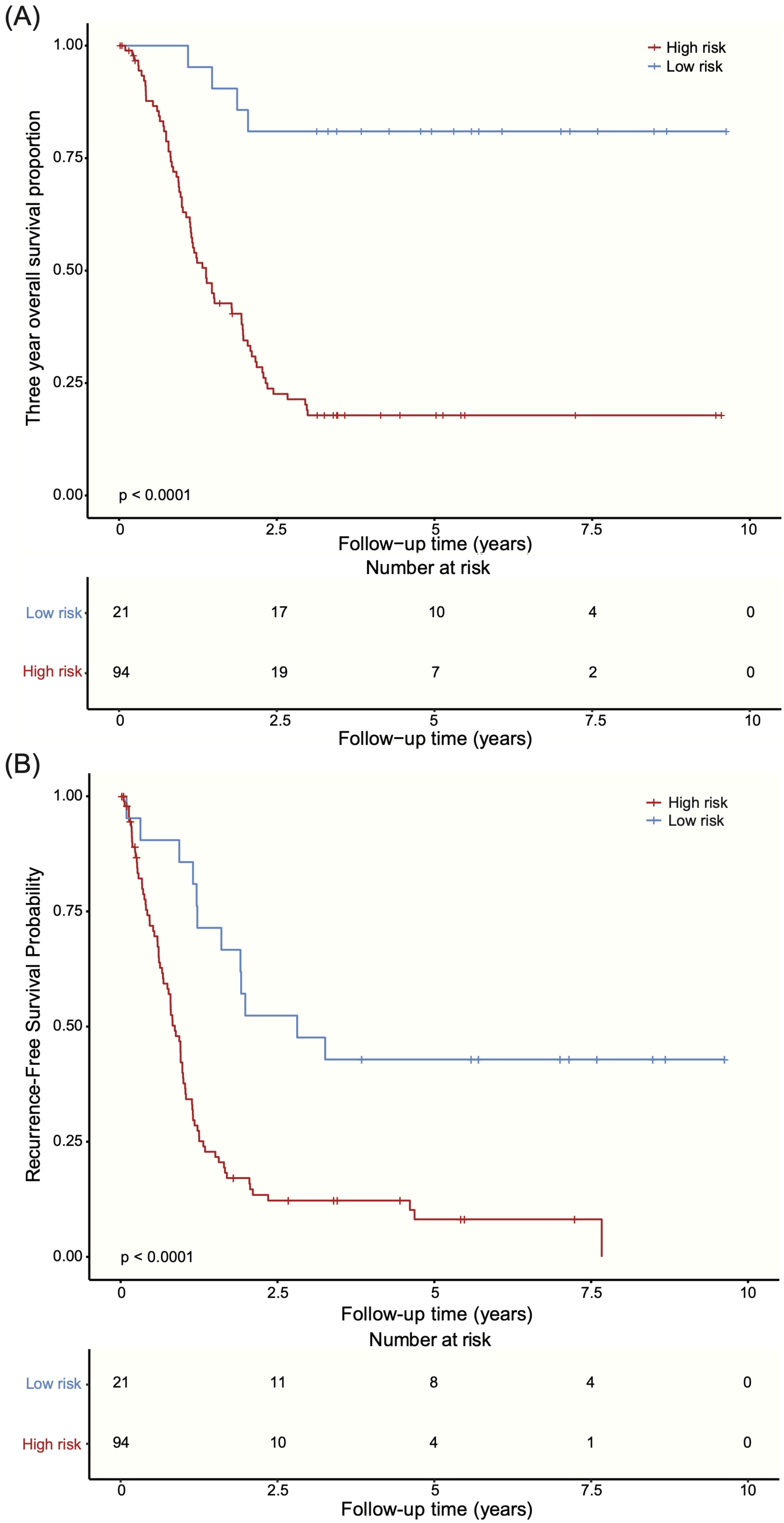
(A)
1.00
0.75
0.50
0.25
0.00
Three year overall survival proportion
High risk
Low risk
p < 0.0001
0
2.5
5
7.5
10
Follow−up time (years)
Number at risk
Low risk
21
17
10
4
0
High risk
94
19
7
2
0
0
2.5
5
7.5
10
Follow−up time (years)
(B)
1.00
0.75
0.50
0.25
0.00
Recurrence-Free Survival Probability
High risk
Low risk
p < 0.0001
0
2.5
5
7.5
10
Follow−up time (years)
Number at risk
Low risk
21
11
8
4
0
High risk
94
10
4
1
0
0
2.5
5
7.5
10
Follow−up time (years)

Supplementary Figure 8 shows Kaplan-Meier curves displaying **(A)** the three-year survival for groups dichotomised by proteomic risk score in ProCan data. **(B)** The recurrence-free survival for groups dichotomized by proteomic risk score in ProCan data.
