# Supplementary material for: Mapping the Proteomic Landscape of Pancreatic Cancer: Prognostic Insights and Subtype Stratification
Source: Cancer Res Commun. 2025 Oct 23;5(10):1879–93. doi: 10.1158/2767-9764.CRC-25-0229 (PMC12548992; doi:10.1158/2767-9764.CRC-25-0229)
Supplement: Supplementary Figure 9 — Kaplan-Meier plot for patients dichotomized by a proteomic risk score that uses only the ten proteins detected in blood by mass spectrometry (PURB, GALM, SERPINA3, OAS3, KRT2, NUDT2, SERPINA4, CUTA, POSTN, CLEC11A). [file crc-25-0229_supplementary_figure_9_suppsf9.pdf]

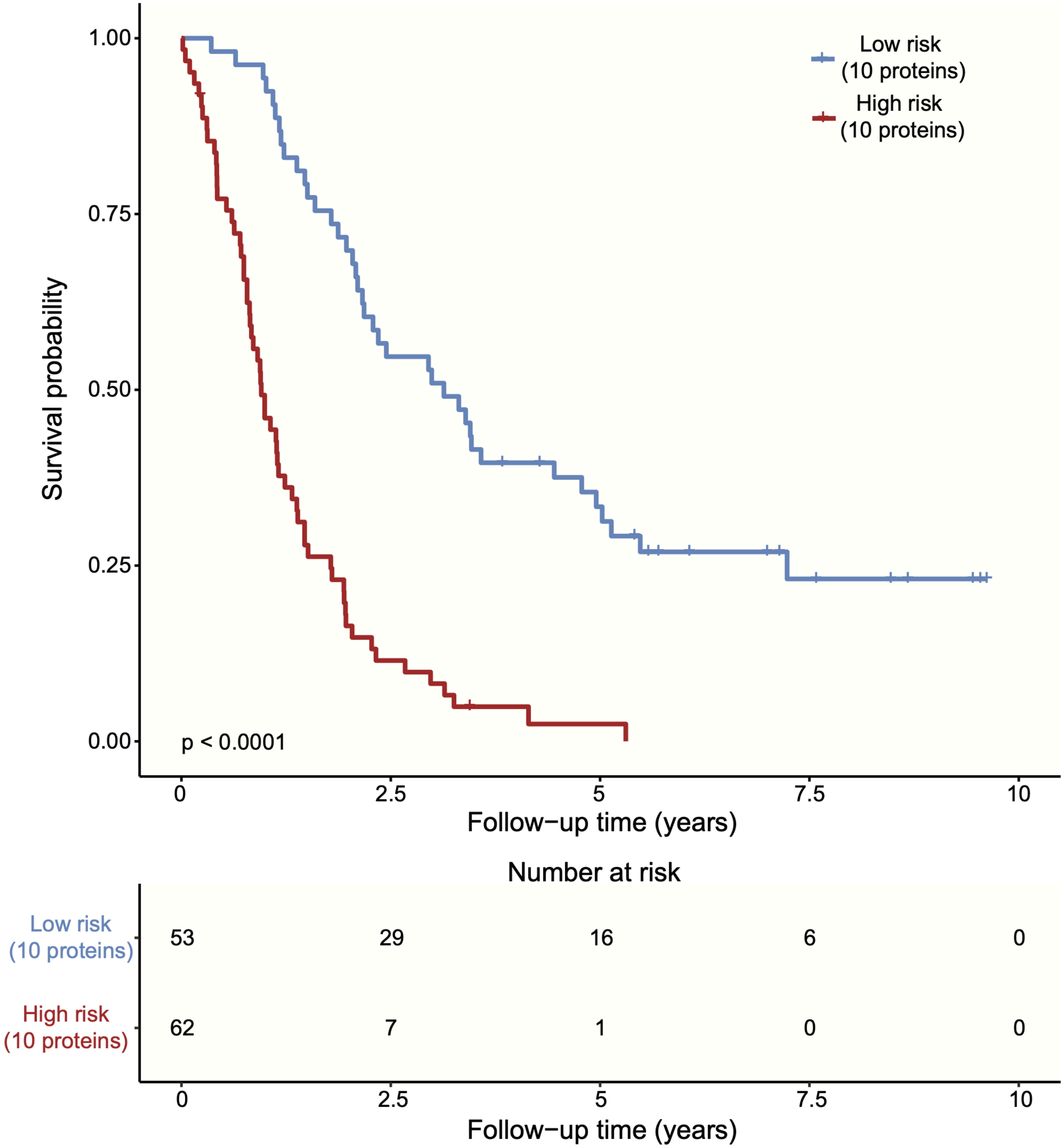

Survival probability
1.00
0.75
0.50
0.25
0.00
Low risk (10 proteins)
High risk (10 proteins)
p < 0.0001
0
2.5
5
7.5
10
Follow−up time (years)
Number at risk
Low risk (10 proteins)
53
29
16
6
0
High risk (10 proteins)
62
7
1
0
0
0
2.5
5
7.5
10
Follow−up time (years)

Supplementary Figure 9: Kaplan-Meier plot for patients dichotomized by a proteomic risk score that uses only the ten proteins detected in blood by mass spectrometry (PURB, GALM, SERPINA3, OAS3, KRT2, NUDT2, SERPINA4, CUTA, POSTN, CLEC11A).
