# Supplementary material for: Mapping the Proteomic Landscape of Pancreatic Cancer: Prognostic Insights and Subtype Stratification
Source: Cancer Res Commun. 2025 Oct 23;5(10):1879–93. doi: 10.1158/2767-9764.CRC-25-0229 (PMC12548992; doi:10.1158/2767-9764.CRC-25-0229)
Supplement: Supplementary Figure 10 — shows the differential abundance and pathway enrichment analyses based on KRAS mutations. (A) Volcano plot displaying the differentially abundant proteins between KRAS mutant PDA and KRAS wild-type PDA. (B) Pathways enriched in Gene Ontology molecular function database from upregulated proteins in tumors that harbored KRAS mutations. [file crc-25-0229_supplementary_figure_10_suppsf10.pdf]

(A)

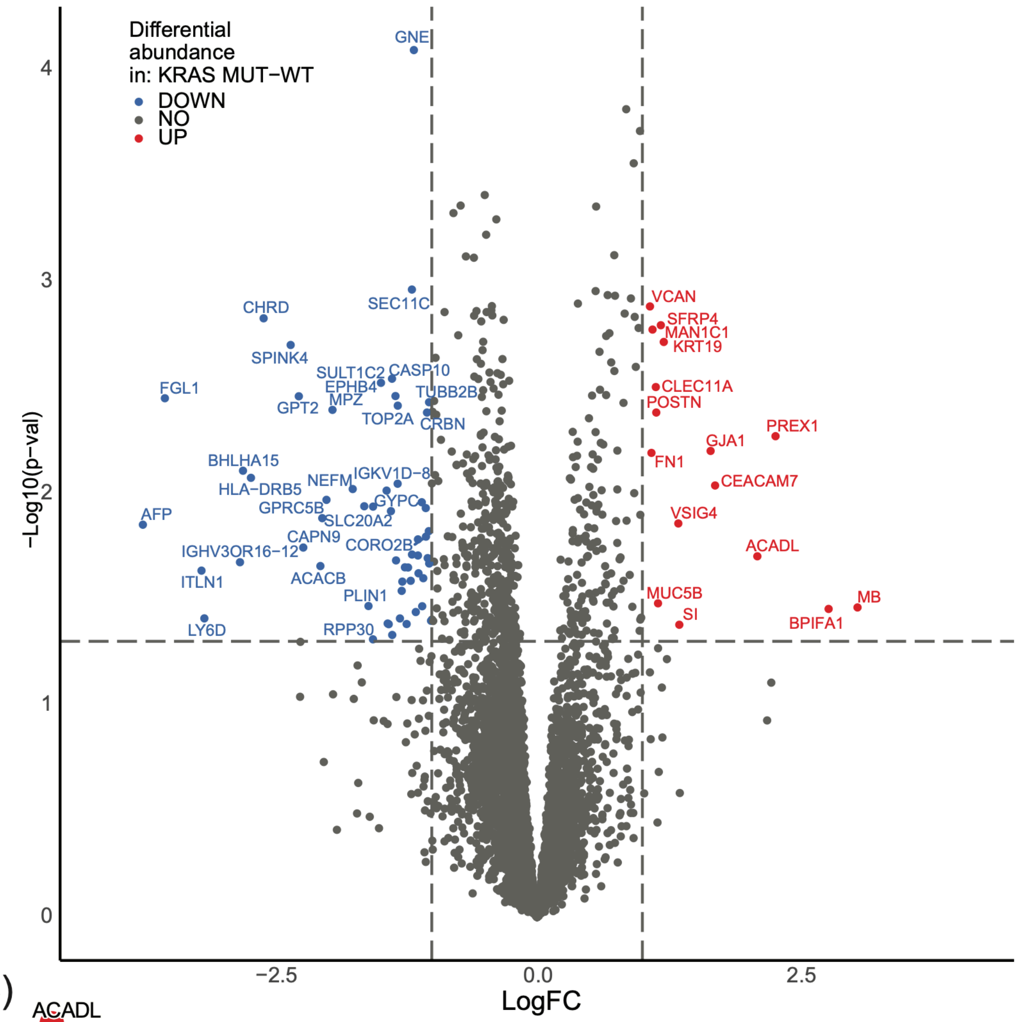
Differential abundance in: KRAS MUT−WT
DOWN
NO
UP
−Log10(p−val)
LogFC
GNE
SEC11C
CHRD
SPINK4
FGL1
SULT1C2
CASP10
EPHB4
MPZ
GPT2
TUBB2B
TOP2A
CRBN
BHLHA15
HLA−DRB5
NEFM
IGKV1D−8
GPRC5B
GYPC
SLC20A2
AFP
CAPN9
CORO2B
IGHV3OR16−12
ACACB
ITLN1
PLIN1
LY6D
RPP30
VCAN
SFRP4
MAN1C1
KRT19
CLEC11A
POSTN
PREX1
GJA1
FN1
CEACAM7
VSIG4
ACADL
MUC5B
SI
MB
BPIFA1

(B)

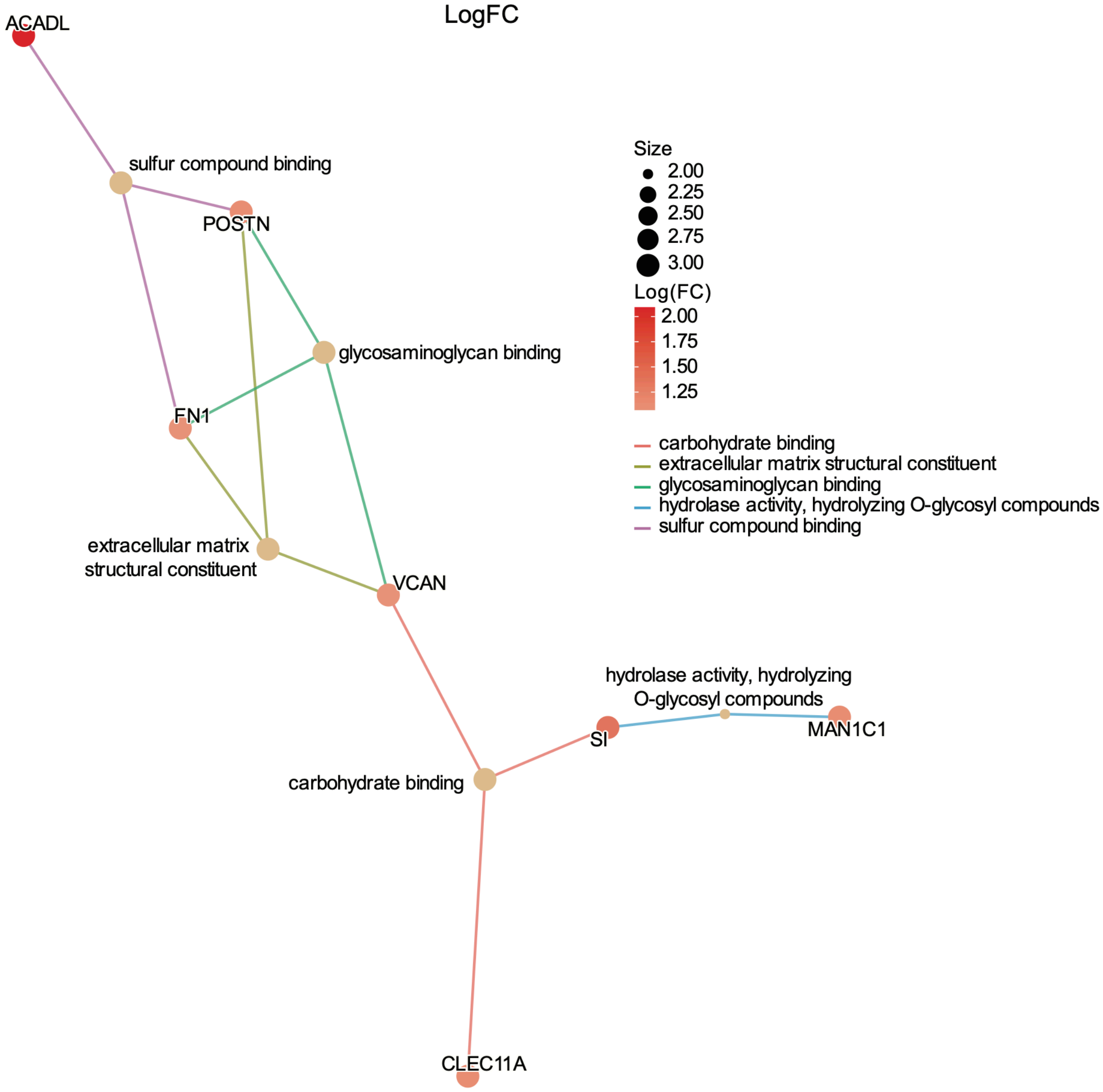
ACADL
sulfur compound binding
POSTN
glycosaminoglycan binding
FN1
extracellular matrix structural constituent
VCAN
hydrolase activity, hydrolyzing O-glycosyl compounds
SI
MAN1C1
carbohydrate binding
CLEC11A
Size
Log(FC)
carbohydrate binding
extracellular matrix structural constituent
glycosaminoglycan binding
hydrolase activity, hydrolyzing O-glycosyl compounds
sulfur compound binding

Supplementary Figure 10 shows the differential abundance and pathway enrichment analyses based on KRAS mutations. **(A)** Volcano plot displaying the differentially abundant proteins between KRAS mutant PDA and KRAS wild-type PDA. **(B)** Pathways enriched in Gene Ontology molecular function database from upregulated proteins in tumors that harbored KRAS mutations.
