# Supplementary material for: Mapping the Proteomic Landscape of Pancreatic Cancer: Prognostic Insights and Subtype Stratification
Source: Cancer Res Commun. 2025 Oct 23;5(10):1879–93. doi: 10.1158/2767-9764.CRC-25-0229 (PMC12548992; doi:10.1158/2767-9764.CRC-25-0229)
Supplement: Supplementary Figure 11 — shows the differential abundance and pathway enrichment analyses based on the KRAS-G12C status. (A) Volcano plot displaying the differentially abundant proteins between tumors harboring the KRAS-G12C mutation versus any other KRAS-G12 mutation. (B) Pathways enriched in the KEGG, Reactome, and WikiPathways databases based on the upregulated proteins from tumors that harbored KRAS-G12C mutations when compared to tumors with other KRAS12 mutations. [file crc-25-0229_supplementary_figure_11_suppsf11.pdf]

(A)

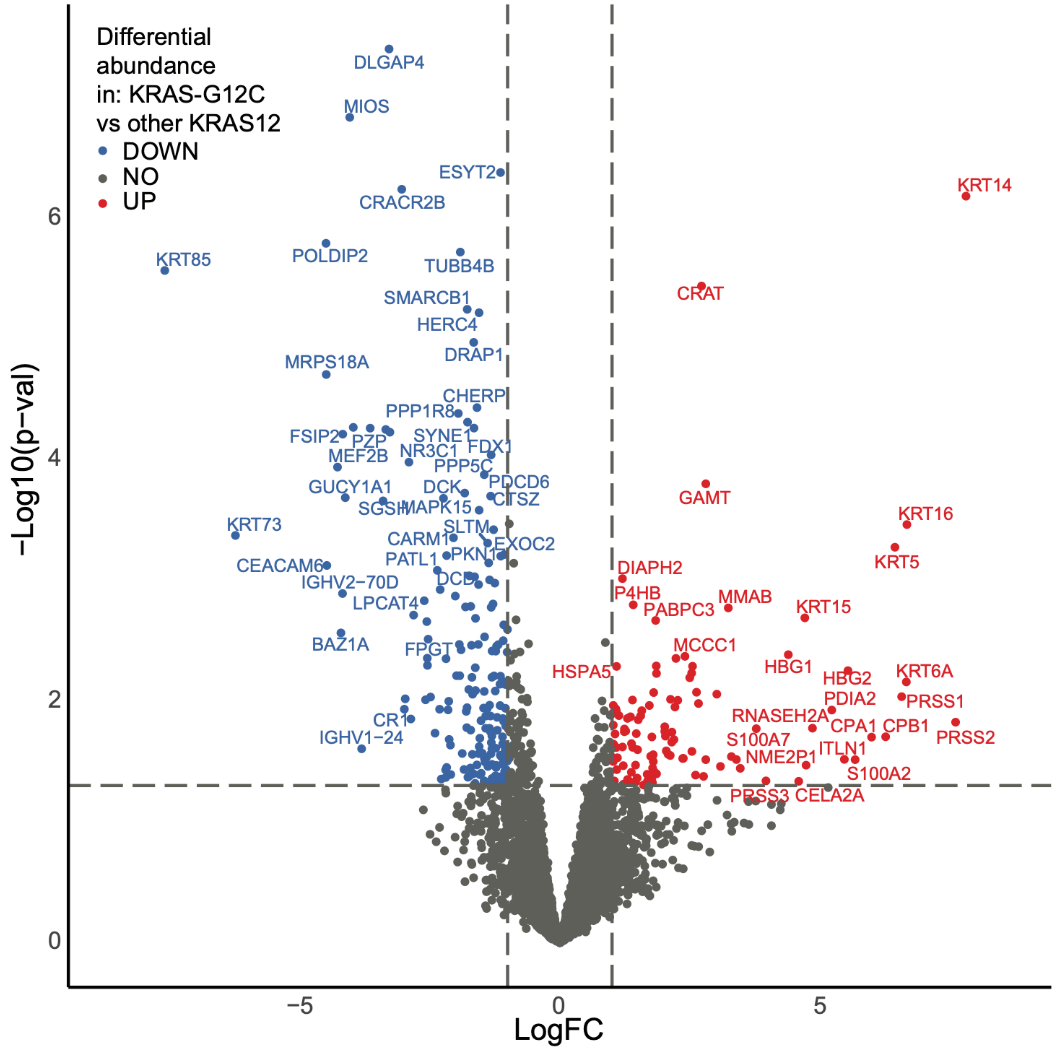
Differential abundance in: KRAS-G12C vs other KRAS12
DOWN
NO
UP
−Log10(p−val)
LogFC
0
2
4
6
−5
0
5
DLGAP4
MIOS
ESYT2
CRACR2B
KRT85
POLDIP2
TUBB4B
SMARCB1
HERC4
DRAP1
MRPS18A
CHERP
PPP1R8
FSIP2
PZP
SYNE1
MEF2B
NR3C1
FDX1
PPP5C
PDCD6
GUCY1A1
DCK
CTSZ
SGSH
MAPK15
KRT73
SLTM
CARM1
EXOC2
PATL1
PKN1
CEACAM6
IGHV2−70D
DCD
LPCAT4
BAZ1A
FPGT
CR1
IGHV1−24
KRT14
CRAT
GAMT
KRT16
KRT5
DIAPH2
P4HB
PABPC3
MMAB
KRT15
MCCC1
HSPA5
HBG1
HBG2
KRT6A
PDIA2
PRSS1
RNASEH2A
CPA1
CPB1
PRSS2
S100A7
NME2P1
ITLN1
S100A2
PRSS3
CELA2A

(B)

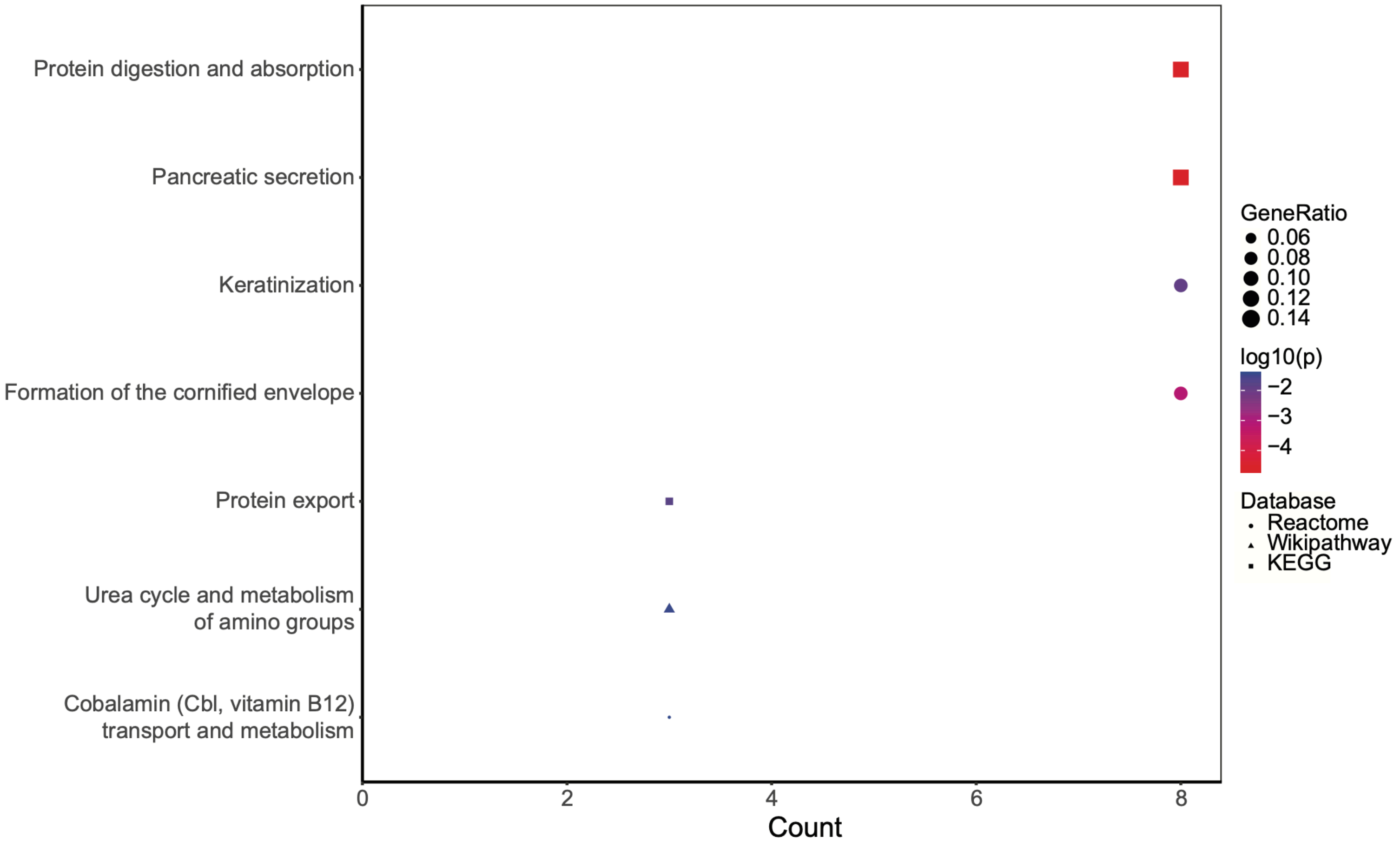
Protein digestion and absorption
Pancreatic secretion
Keratinization
Formation of the cornified envelope
Protein export
Urea cycle and metabolism of amino groups
Cobalamin (Cbl, vitamin B12) transport and metabolism
0
2
4
6
8
Count
GeneRatio
0.06
0.08
0.10
0.12
0.14
log10(p)
−2
−3
−4
Database
Reactome
Wikipathway
KEGG

Supplementary Figure 11 shows the differential abundance and pathway enrichment analyses based on the KRAS-G12C status. **(A)** Volcano plot displaying the differentially abundant proteins between tumors harboring the KRAS-G12C mutation versus any other KRAS-G12 mutation. **(B)** Pathways enriched in the KEGG, Reactome, and WikiPathways databases based on the upregulated proteins from tumors that harbored KRAS-G12C mutations when compared to tumors with other KRAS12 mutations.
