# Supplementary material for: Mapping the Proteomic Landscape of Pancreatic Cancer: Prognostic Insights and Subtype Stratification
Source: Cancer Res Commun. 2025 Oct 23;5(10):1879–93. doi: 10.1158/2767-9764.CRC-25-0229 (PMC12548992; doi:10.1158/2767-9764.CRC-25-0229)
Supplement: Supplementary Figure 12 — shows a volcano plot displaying the differentially abundant proteins between tumors harboring a KRAS-G12D mutation versus those with any other KRAS-G12 mutation [file crc-25-0229_supplementary_figure_12_suppsf12.pdf]

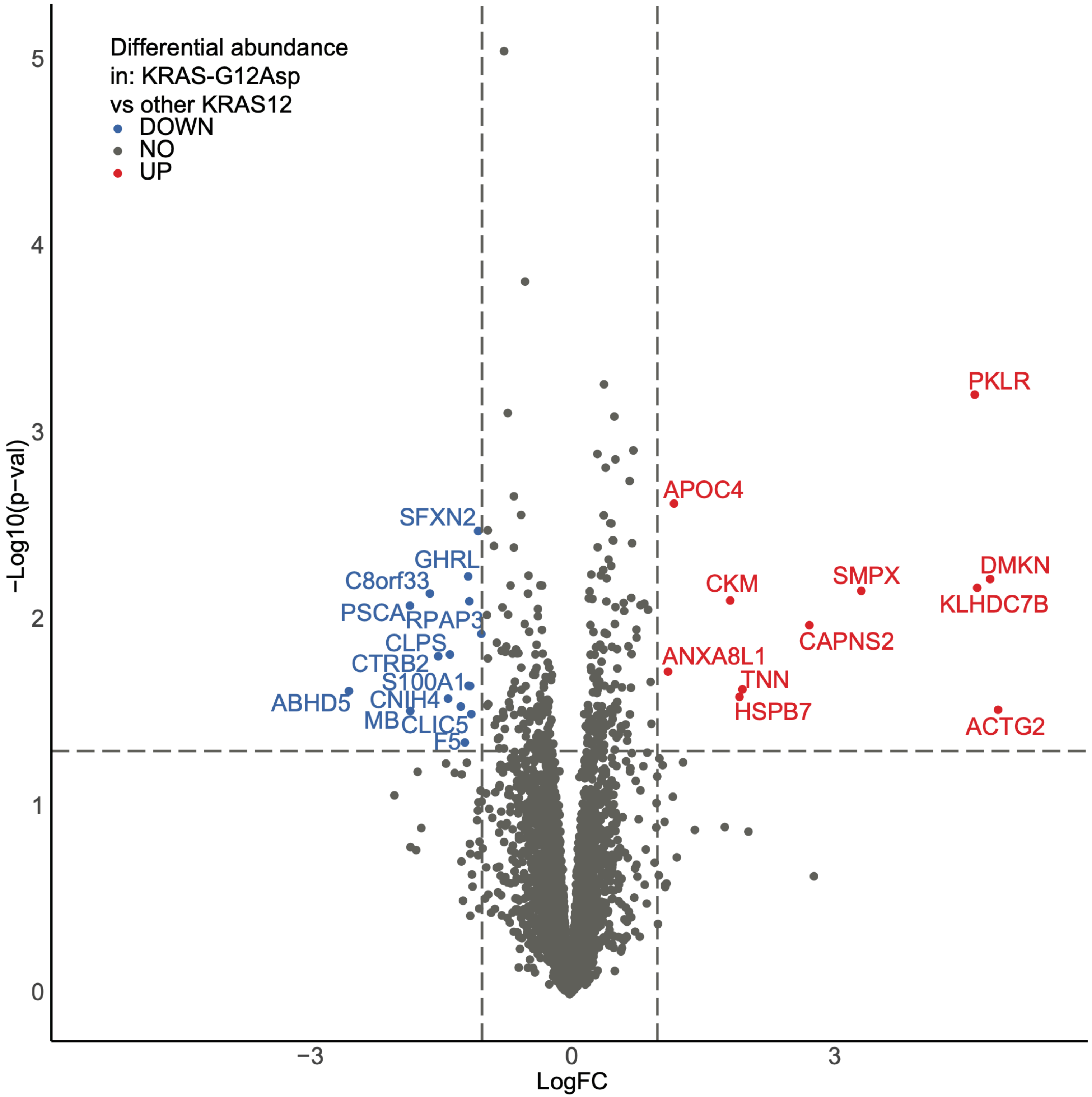
Differential abundance
in: KRAS-G12Asp
vs other KRAS12
DOWN
NO
UP
−Log10(p−val)
LogFC
5
4
3
2
1
0
−3
0
3
PKLR
APOC4
SFXN2
GHRL
C8orf33
PSCA
RPAP3
CLPS
CTRB2
S100A1
ABHD5
CNIH4
MB
CLIC5
F5
CKM
SMPX
DMKN
KLHDC7B
CAPNS2
ANXA8L1
TNN
HSPB7
ACTG2

Supplementary Figure 12 shows a volcano plot displaying the differentially abundant proteins between tumors harboring a KRAS-G12D mutation versus those with any other KRAS-G12 mutation.
