# Supplementary material for: Mapping the Proteomic Landscape of Pancreatic Cancer: Prognostic Insights and Subtype Stratification
Source: Cancer Res Commun. 2025 Oct 23;5(10):1879–93. doi: 10.1158/2767-9764.CRC-25-0229 (PMC12548992; doi:10.1158/2767-9764.CRC-25-0229)
Supplement: Supplementary Figure 13 — shows the differential abundance and pathway enrichment analyses based on HRD status. (A) Volcano plot displaying the differentially abundant proteins between tumor samples with and without HRD. (B) Pathways enriched in KEGG, Reactome, and Wikipathways databases based on the upregulated proteins in tumors from patients with HRD-positive status. [file crc-25-0229_supplementary_figure_13_suppsf13.pdf]

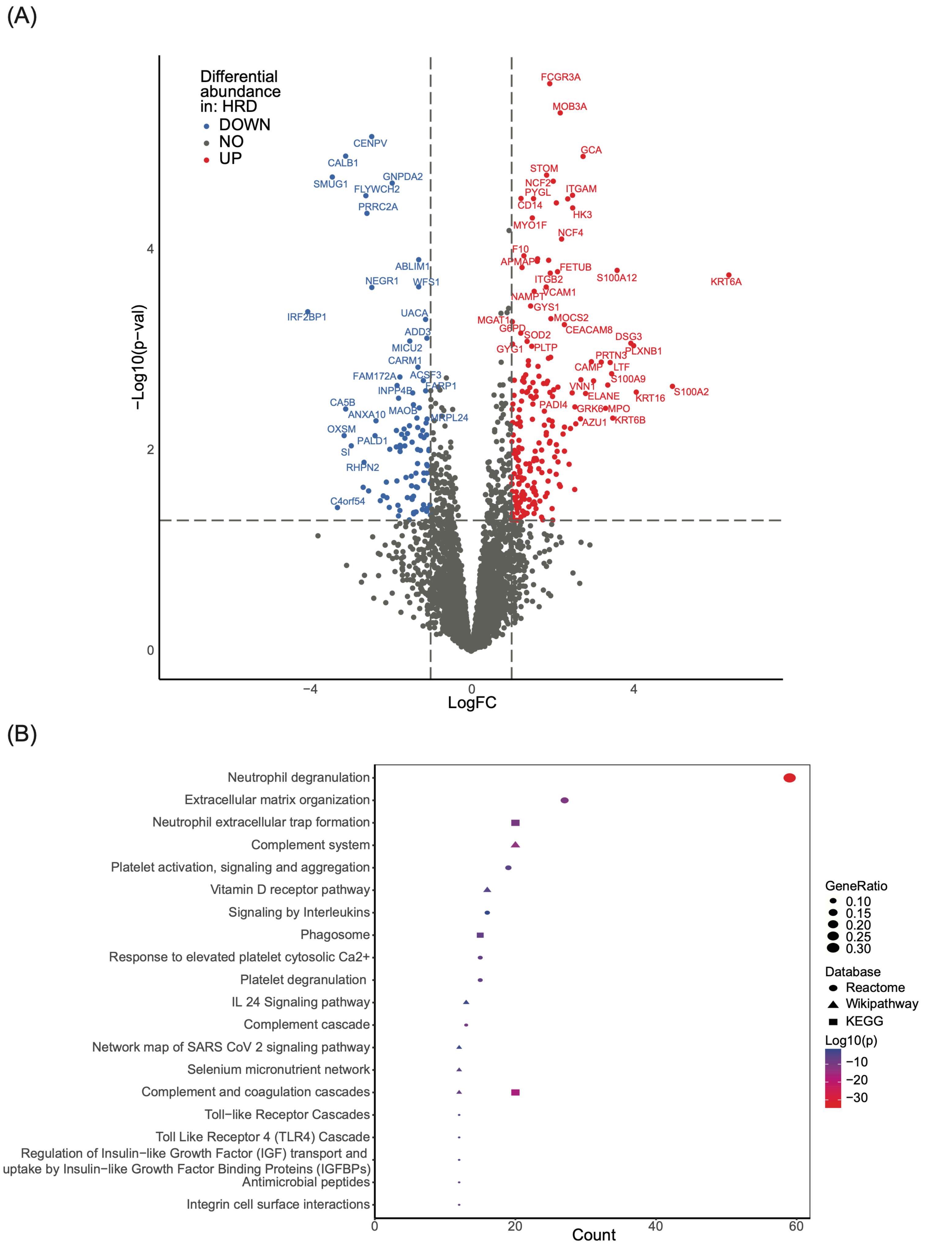
(A)
Differential abundance in: HRD
DOWN
NO
UP
−Log10(p−val)
LogFC
FCGR3A
MOB3A
GCA
STOM
NCF2
ITGAM
PYGL
CD14
HK3
MYO1F
NCF4
F10
APMAP
FETUB
S100A12
KRT6A
ITGB2
NAMPT
VCAM1
GYS1
MOCS2
MGAT1
G6PD
CEACAM8
SOD2
DSG3
GYG1
PLTP
PLXNB1
PRTN3
CAMP
LTF
S100A9
VNN1
ELANE
KRT16
S100A2
PADI4
GRK6
MPO
AZU1
KRT6B
CENPV
CALB1
GNPDA2
SMUG1
FLYWCH2
PRRC2A
ABLIM1
NEGR1
WFS1
IRF2BP1
UACA
ADD3
MICU2
CARM1
FAM172A
ACSF3
FARP1
INPP4B
CA5B
ANXA10
MAOB
MRPL24
OXSM
PALD1
SI
RHPN2
C4orf54
(B)
Neutrophil degranulation
Extracellular matrix organization
Neutrophil extracellular trap formation
Complement system
Platelet activation, signaling and aggregation
Vitamin D receptor pathway
Signaling by Interleukins
Phagosome
Response to elevated platelet cytosolic Ca2+
Platelet degranulation
IL 24 Signaling pathway
Complement cascade
Network map of SARS CoV 2 signaling pathway
Selenium micronutrient network
Complement and coagulation cascades
Toll−like Receptor Cascades
Toll Like Receptor 4 (TLR4) Cascade
Regulation of Insulin−like Growth Factor (IGF) transport and uptake by Insulin−like Growth Factor Binding Proteins (IGFBPs)
Antimicrobial peptides
Integrin cell surface interactions
Count
GeneRatio
0.10
0.15
0.20
0.25
0.30
Database
Reactome
Wikipathway
KEGG
Log10(p)
−10
−20
−30

Supplementary Figure 13 shows the differential abundance and pathway enrichment analyses based on HRD status. **(A)** Volcano plot displaying the differentially abundant proteins between tumor samples with and without HRD. **(B)** Pathways enriched in KEGG, Reactome, and Wikipathways databases based on the upregulated proteins in tumors from patients with HRD-positive status.
