# Supplementary material for: Mapping the Proteomic Landscape of Pancreatic Cancer: Prognostic Insights and Subtype Stratification
Source: Cancer Res Commun. 2025 Oct 23;5(10):1879–93. doi: 10.1158/2767-9764.CRC-25-0229 (PMC12548992; doi:10.1158/2767-9764.CRC-25-0229)
Supplement: Supplementary Figure 14 — shows the differential abundance and pathway enrichment analyses based on COSMIC Signature-3 status. (A) Volcano plot displaying the differentially abundant proteins between tumors with and without somatic mutations consistent with COSMIC Signature-3. (B) Pathways enriched in Reactome from upregulated proteins in tumors that displayed mutations corresponding to COSMIC Signature-3. [file crc-25-0229_supplementary_figure_14_suppsf14.pdf]

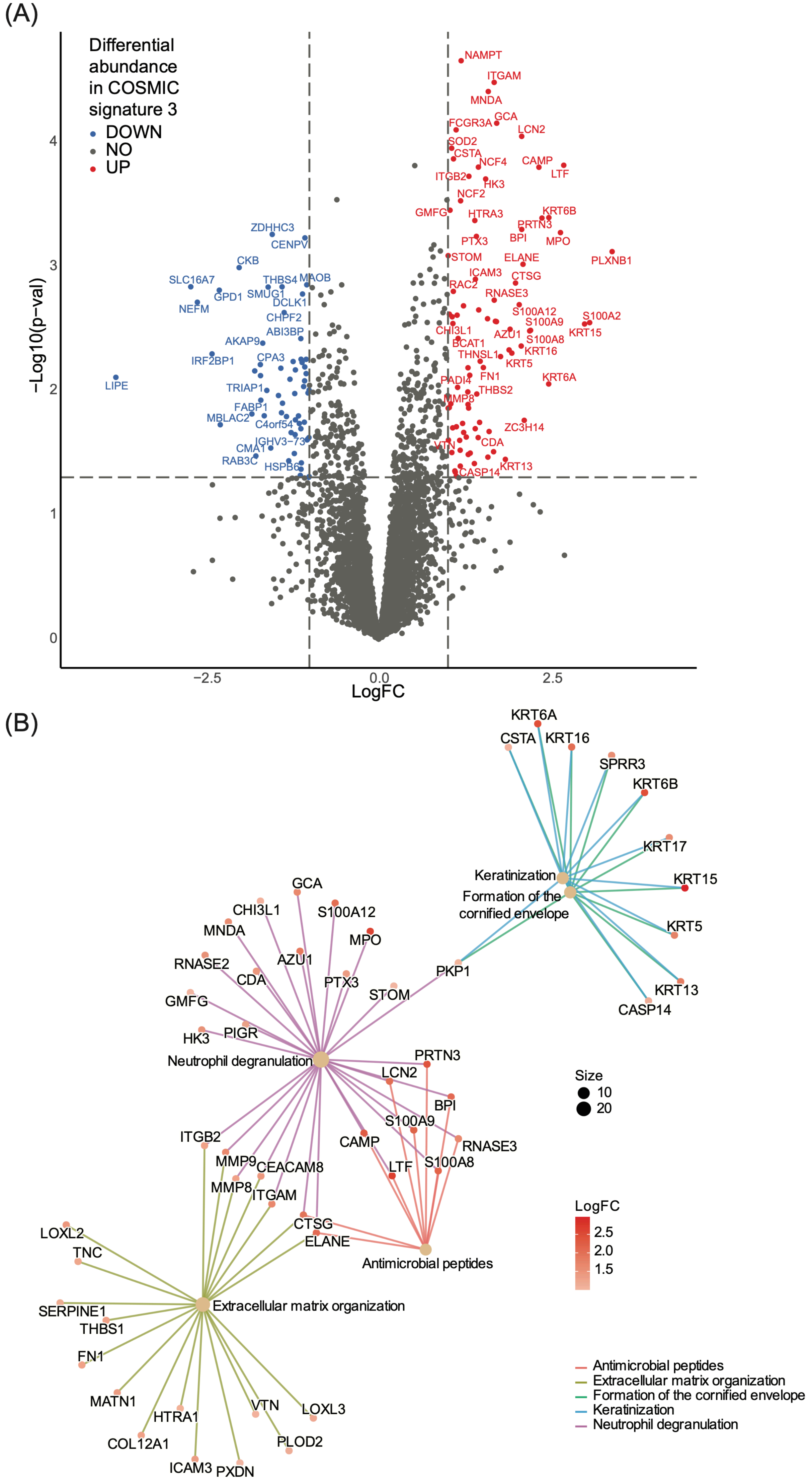
(A)
Differential abundance in COSMIC signature 3
DOWN
NO
UP
−Log10(p−val)
LogFC
−2.5
0.0
2.5
0
1
2
3
4
NAMPT
ITGAM
MNDA
GCA
FCGR3A
LCN2
SOD2
CSTA
NCF4
CAMP
LTF
ITGB2
HK3
NCF2
GMFG
HTRA3
KRT6B
PRTN3
PTX3
BPI
MPO
PLXNB1
STOM
ELANE
ICAM3
CTSG
RAC2
RNASE3
S100A12
S100A9
S100A2
KRT15
AZU1
S100A8
CHI3L1
BCAT1
KRT16
THNSL1
KRT5
FN1
KRT6A
PADI4
THBS2
MMP8
ZC3H14
VTN
CDA
KRT13
CASP14
ZDHHC3
CENPV
CKB
SLC16A7
THBS4
MAOB
GPD1
SMUG1
DCLK1
NEFM
CHPF2
ABI3BP
AKAP9
IRF2BP1
CPA3
LIPE
TRIAP1
FABP1
MBLAC2
C4orf54
IGHV3−73
CMA1
RAB3C
HSPB6
(B)
KRT6A
CSTA
KRT16
SPRR3
KRT6B
KRT17
KRT15
KRT5
KRT13
CASP14
Keratinization
Formation of the cornified envelope
PKP1
GCA
CHI3L1
S100A12
MNDA
MPO
AZU1
RNASE2
CDA
PTX3
STOM
GMFG
PIGR
HK3
Neutrophil degranulation
PRTN3
LCN2
BPI
S100A9
RNASE3
CAMP
LTF
S100A8
ITGB2
MMP9
CEACAM8
MMP8
ITGAM
CTSG
ELANE
Antimicrobial peptides
LOXL2
TNC
SERPINE1
THBS1
FN1
MATN1
HTRA1
COL12A1
ICAM3
PXDN
VTN
LOXL3
PLOD2
Extracellular matrix organization
Size
10
20
LogFC
2.5
2.0
1.5
Antimicrobial peptides
Extracellular matrix organization
Formation of the cornified envelope
Keratinization
Neutrophil degranulation

Supplementary Figure 14 shows the differential abundance and pathway enrichment analyses based on COSMIC Signature-3 status. **(A)** Volcano plot displaying the differentially abundant proteins between tumors with and without somatic mutations consistent with COSMIC Signature-3. **(B)** Pathways enriched in Reactome from upregulated proteins in tumors that displayed mutations corresponding to COSMIC Signature-3.
