# Supplementary material for: Mapping the Proteomic Landscape of Pancreatic Cancer: Prognostic Insights and Subtype Stratification
Source: Cancer Res Commun. 2025 Oct 23;5(10):1879–93. doi: 10.1158/2767-9764.CRC-25-0229 (PMC12548992; doi:10.1158/2767-9764.CRC-25-0229)
Supplement: Supplementary Figure 15 — shows a Forest plot presentation of the hazard ratios for each S100 protein detected in the tumor samples from the study cohort, derived from a univariate Cox regression analysis with overall survival as the outcome of interest. [file crc-25-0229_supplementary_figure_15_suppsf15.pdf]

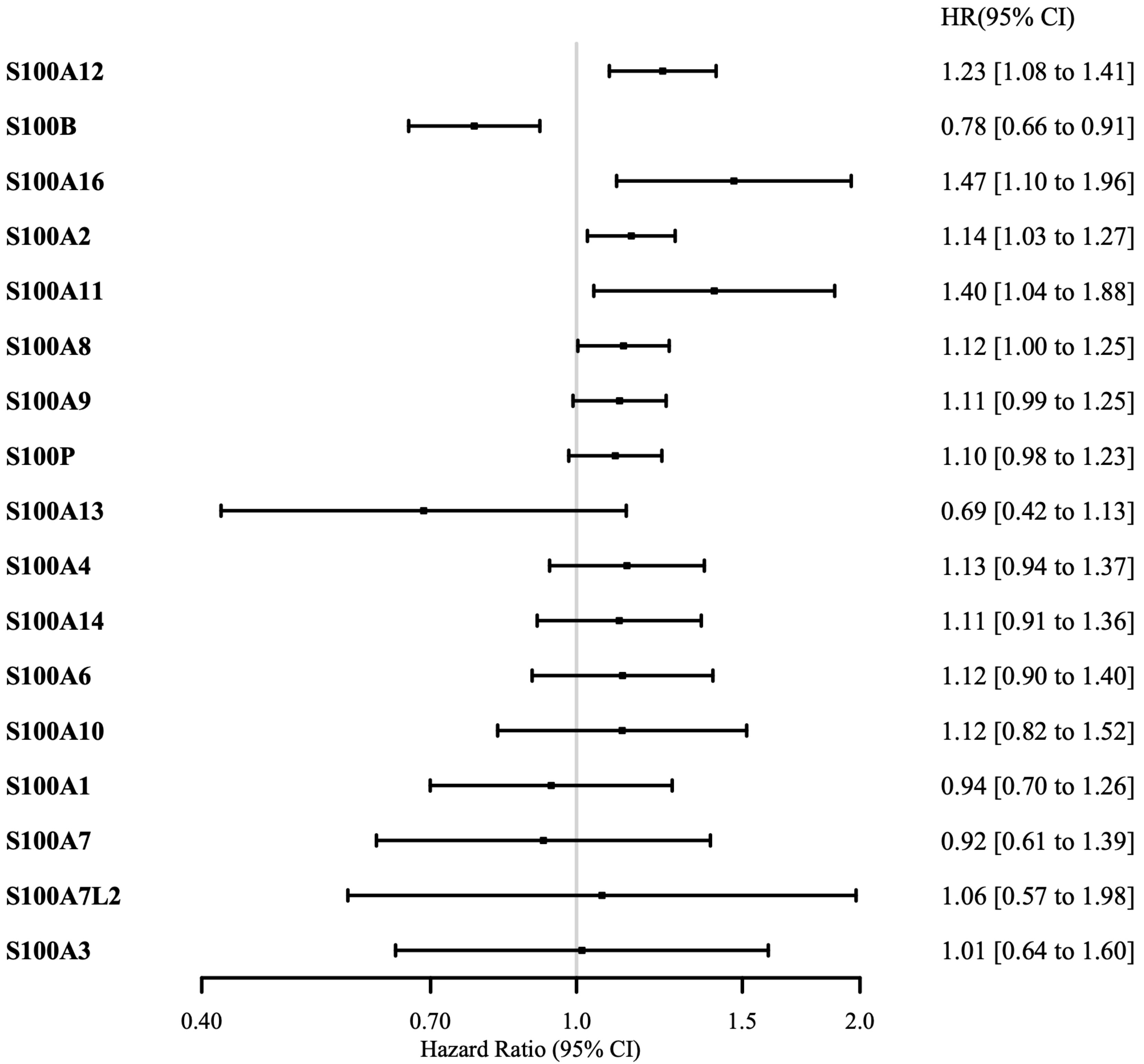

HR(95% CI)
S100A12
1.23 [1.08 to 1.41]
S100B
0.78 [0.66 to 0.91]
S100A16
1.47 [1.10 to 1.96]
S100A2
1.14 [1.03 to 1.27]
S100A11
1.40 [1.04 to 1.88]
S100A8
1.12 [1.00 to 1.25]
S100A9
1.11 [0.99 to 1.25]
S100P
1.10 [0.98 to 1.23]
S100A13
0.69 [0.42 to 1.13]
S100A4
1.13 [0.94 to 1.37]
S100A14
1.11 [0.91 to 1.36]
S100A6
1.12 [0.90 to 1.40]
S100A10
1.12 [0.82 to 1.52]
S100A1
0.94 [0.70 to 1.26]
S100A7
0.92 [0.61 to 1.39]
S100A7L2
1.06 [0.57 to 1.98]
S100A3
1.01 [0.64 to 1.60]
0.40
0.70
1.0
1.5
2.0
Hazard Ratio (95% CI)

Supplementary Figure 15 shows a Forest plot presentation of the hazard ratios for each S100 protein detected in the tumor samples from the study cohort, derived from a univariate Cox regression analysis with overall survival as the outcome of interest.
