# Supplementary material for: Mapping the Proteomic Landscape of Pancreatic Cancer: Prognostic Insights and Subtype Stratification
Source: Cancer Res Commun. 2025 Oct 23;5(10):1879–93. doi: 10.1158/2767-9764.CRC-25-0229 (PMC12548992; doi:10.1158/2767-9764.CRC-25-0229)
Supplement: Supplementary Figure 16 — shows a Forest plot presentation of the hazard ratios for each KRT protein detected in the tumor samples from the study cohort, derived from a univariate Cox regression analysis with overall survival as the outcome of interest. Note that this figure excludes KRT75, which was detected in only two samples. [file crc-25-0229_supplementary_figure_16_suppsf16.pdf]

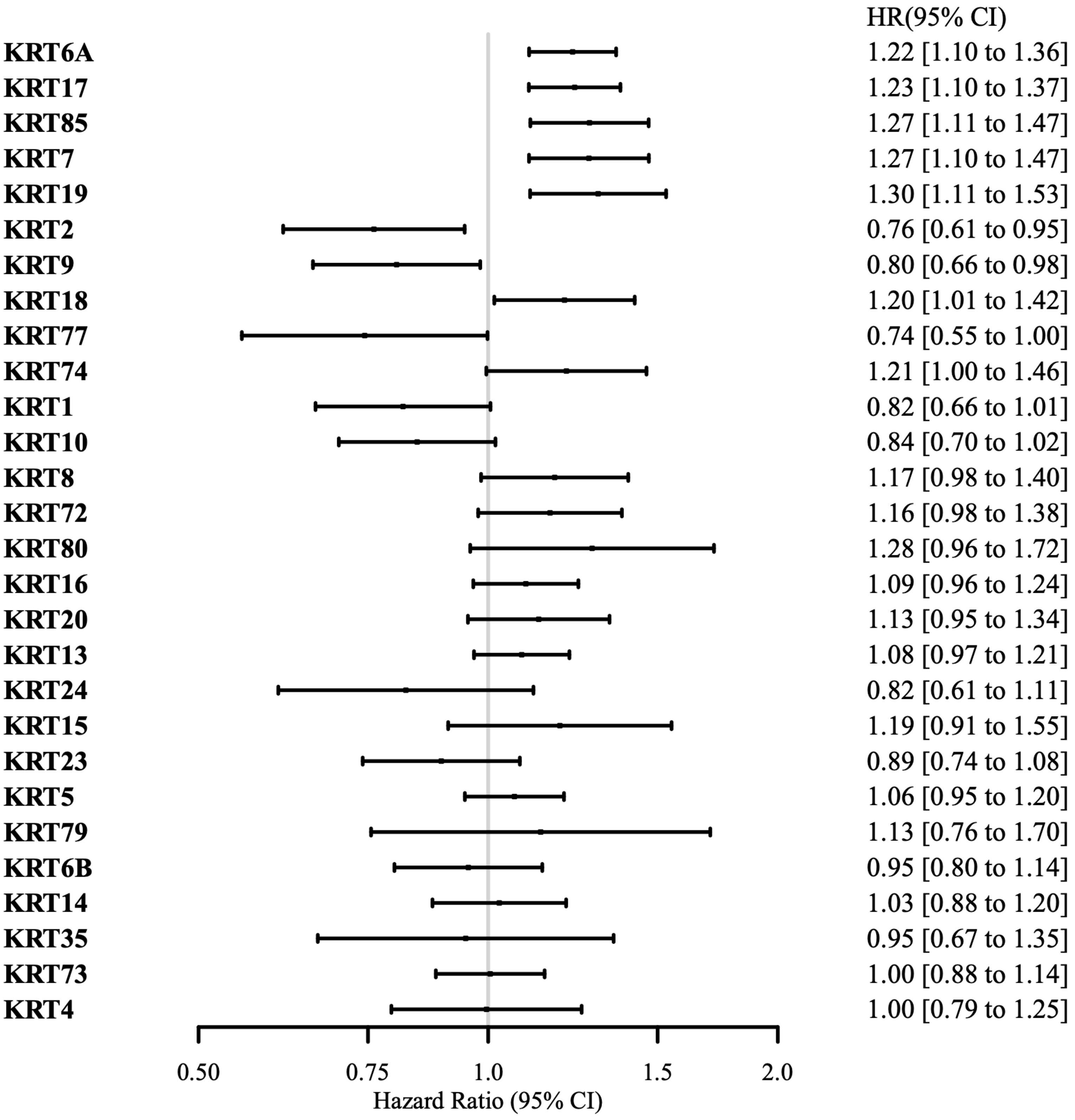
HR(95% CI)
KRT6A
KRT17
KRT85
KRT7
KRT19
KRT2
KRT9
KRT18
KRT77
KRT74
KRT1
KRT10
KRT8
KRT72
KRT80
KRT16
KRT20
KRT13
KRT24
KRT15
KRT23
KRT5
KRT79
KRT6B
KRT14
KRT35
KRT73
KRT4
1.22 [1.10 to 1.36]
1.23 [1.10 to 1.37]
1.27 [1.11 to 1.47]
1.27 [1.10 to 1.47]
1.30 [1.11 to 1.53]
0.76 [0.61 to 0.95]
0.80 [0.66 to 0.98]
1.20 [1.01 to 1.42]
0.74 [0.55 to 1.00]
1.21 [1.00 to 1.46]
0.82 [0.66 to 1.01]
0.84 [0.70 to 1.02]
1.17 [0.98 to 1.40]
1.16 [0.98 to 1.38]
1.28 [0.96 to 1.72]
1.09 [0.96 to 1.24]
1.13 [0.95 to 1.34]
1.08 [0.97 to 1.21]
0.82 [0.61 to 1.11]
1.19 [0.91 to 1.55]
0.89 [0.74 to 1.08]
1.06 [0.95 to 1.20]
1.13 [0.76 to 1.70]
0.95 [0.80 to 1.14]
1.03 [0.88 to 1.20]
0.95 [0.67 to 1.35]
1.00 [0.88 to 1.14]
1.00 [0.79 to 1.25]
0.50
0.75
1.0
1.5
2.0
Hazard Ratio (95% CI)

Supplementary Figure 16 shows a Forest plot presentation of the hazard ratios for each KRT protein detected in the tumor samples from the study cohort, derived from a univariate Cox regression analysis with overall survival as the outcome of interest. Note that this figure excludes KRT75, which was detected in only two samples.
